# A ligandable PNT domain establishes ERG as a directly targetable oncogenic driver in prostate cancer

**DOI:** 10.1073/pnas.2537437123

**Published:** 2026-07-07

**Authors:** Xiaoju Wang, Wenyan Liu, Jiehao Yang, Jean Ching-Yi Tien, Yu Chang, Rahul Mannan, Somnath Mahapatra, Yang Zhou, Lihao Gan, Xuhong Cao, Jiayi Zhou, Yuping Zhang, Sharpkate Shaker, Yichao Huang, Hang Qiao, Rudana Hamadeh, Grafton Ervine, Cynthia Wang, Fengyun Su, Rui Wang, Lanbo Xiao, Raghunath Ranga Sudharshan, Arvind Rao, Zaneta Nikolovska-Coleska, Cole Stephens, Lifeng Pan, James J. Chou, Debashish Sahu, Jeanne Stuckey, Zhen Wang, Ke Ding, Arul M. Chinnaiyan

**Affiliations:** ^a^ Michigan Center for Translational Pathology, University of Michigan, Ann Arbor, MI 48109; ^b^ Department of Pathology, University of Michigan, Ann Arbor, MI 48109; ^c^ Rogel Cancer Center, University of Michigan, Ann Arbor, MI 48109; ^d^ State Key Laboratory of Chemical Biology, Shanghai Institute of Organic Chemistry, Chinese Academy of Sciences, Shanghai 200032, People’s Republic of China; ^e^International Cooperative Laboratory of Traditional Chinese Medicine Modernization and Innovative Drug Discovery of Chinese Ministry of Education, Guangzhou City Key Laboratory of Precision Chemical Drug Development, College of Pharmacy, Jinan University, Guangzhou 511400, People’s Republic of China; ^f^ Department of Computational Medicine and Bioinformatics, University of Michigan, Ann Arbor, MI 48109; ^g^ Department of Radiation Oncology, University of Michigan, Ann Arbor, MI 48109; ^h^ Medicinal Chemistry Graduate Program, College of Pharmacy, University of Michigan, Ann Arbor, MI 48109; ^i^ BioNMR Core Facility, Life Sciences Institute, University of Michigan, Ann Arbor, MI 48109; ^j^ Life Sciences Institute, University of Michigan, Ann Arbor, MI 48109; ^k^ HHMI, University of Michigan, Ann Arbor, MI 48109; ^l^ Department of Urology, University of Michigan, Ann Arbor, MI 48109

**Keywords:** ERG transcription factor, gene fusion, PNT domain, small-molecule inhibitor, prostate cancer

## Abstract

The *TMPRSS2:ERG* gene fusion, present in approximately 50% of prostate cancers in patients of European ancestry, drives oncogenesis through aberrant overexpression of the ERG transcription factor. Despite its role as a truncal oncogenic driver, ERG has been considered undruggable due to the absence of enzymatic activity and apparent lack of ligandable pockets. Here, we demonstrate continued dependency on ERG in metastatic prostate cancer and identify a druggable pocket within its N-terminal Pointed (PNT) domain. Using an inducible shRNA system in *TMPRSS2:ERG*-positive VCaP cells, we show that ERG depletion causes profound growth inhibition. To therapeutically exploit this vulnerability, we conducted a domain-focused differential scanning fluorimetry screen targeting the ERG PNT domain, followed by structure–activity relationship optimization. This approach yielded PBITE-1 (PNT-Binding Inhibitor of the Transcription factor ERG), a small molecule that selectively binds the ERG PNT domain. NMR chemical-shift perturbation mapping and molecular docking revealed that PBITE-1 engages a discrete, solvent-exposed surface comprising two α-helices and an adjacent flexible loop, defining a ligand-binding pocket within the PNT domain. In cellular models, PBITE-1 directly engaged ERG, selectively inhibited proliferation and invasion, and induced apoptosis in ERG-driven prostate and hematologic malignancies. PBITE-1 potently suppressed growth of ERG-positive mouse and human-derived prostate cancer organoids. Furthermore, PBITE-1 treatment significantly induced tumor cell apoptosis in VCaP xenograft models. These findings establish the ERG PNT domain as ligandable and provide preclinical evidence that ERG is directly targetable by small molecules, enabling future development of ERG-directed inhibitors and targeted protein degraders.

## Significance

The *TMPRSS2:ERG* gene fusion is the most common oncogenic driver in prostate cancer, yet the ERG transcription factor has long been considered undruggable due to the absence of known ligand-binding sites. This study overturns that paradigm by identifying a structurally defined, ligandable pocket within the ERG N-terminal Pointed (PNT) domain. We describe PBITE-1, a molecular probe compound that directly engages this pocket, disrupts ERG function, and suppresses tumor growth across multiple preclinical prostate cancer models. These findings establish ERG as a tractable therapeutic target and provide a structural and chemical foundation for the future development of ERG-directed inhibitors and targeted protein degraders in prostate cancer and other ERG-driven malignancies.

Prostate cancer remains a leading cause of cancer-related morbidity and mortality in men, with approximately 313,780 new cases and 35,770 deaths annually in the United States (1). The majority of prostate cancers harbor recurrent gene rearrangements involving ETS family transcription factors (2, 3). The most prevalent of these rearrangements–observed in approximately 50% of prostate cancers of European ancestry–fuses the androgen-responsive *TMPRSS2* gene to the coding region of *ERG* (2, 4). Driven by the androgen receptor (AR), this rearrangement leads to pathogenic overexpression of a *TMPRSS2:ERG* chimeric transcript that encodes a nearly full-length ERG protein. Functionally, ERG interacts with AR and reprograms the AR cistrome by facilitating AR binding to an expanded range of AR response elements (5), thereby activating oncogenic gene networks. ERG further alters global transcriptional programs by promoting stemness through interactions with chromatin-remodeling complexes such as the SWI/SNF complex (6, 7). This rewiring of AR signaling and chromatin-modifying machinery enhances proliferation and migration of tumor cells (8, 9).

As a truncal oncogenic driver, ERG overexpression induces prostate tumorigenesis in genetically engineered mouse models, an effect markedly accelerated by concomitant *PTEN* loss (4, 10, 11). Conversely, genetic silencing of ERG using small interfering RNA (siRNA) or antisense oligonucleotides (ASOs) suppresses the growth of ERG fusion–positive prostate cancer in vitro, establishing ERG as a bona fide oncogenic driver and therapeutic vulnerability (12–14). Accordingly, our group previously developed peptidomimetics that bind the ETS domain of ERG, promote protein degradation, and impair ERG-driven prostate tumor growth (15).

Standard therapy for advanced prostate cancer includes androgen deprivation, which initially reduces *TMPRSS2:ERG* expression in fusion-positive tumors. However, resistance inevitably emerges through diverse mechanisms that restore AR signaling and, consequently, *TMPRSS2:ERG* expression (16). Although next-generation AR inhibitors and androgen biosynthesis blockers have improved outcomes in castration-resistant prostate cancer (CRPC) (17, 18), adaptive signaling and lineage plasticity ultimately limit therapeutic durability. Because ERG acts as a persistent oncogenic driver in both hormone-naïve and castration-resistant disease (15), direct pharmacological targeting of ERG represents a compelling therapeutic opportunity.

Despite its critical roles in driving oncogenesis, ERG has long been considered a challenging drug target (19). Many transcription factors lack enzymatic pockets or canonical ligand-binding cavities and instead function via protein–protein and protein–DNA interfaces that are typically incompatible with high-affinity small molecules (20). These features have led to difficulties in developing inhibitors that directly target transcription factors. An emerging approach that addresses these challenges is the development of proteolysis-targeting chimeras (PROTACs) (21). Unlike traditional inhibitors, PROTACs can induce target degradation even when binding occurs via weak, nonfunctional sites, enabling exploitation of previously inaccessible surface pockets. This approach has successfully targeted transcription factors and other classically “undruggable” proteins (22–24).

The *TMPRSS2:ERG* fusion gene encodes a nearly full-length ERG protein lacking only the first 32 N-terminal residues (25). Like other ETS family members, ERG contains a conserved C-terminal ETS DNA binding domain (26–28) and an N-terminal Pointed (PNT) domain, a conserved, structured four alpha-helix bundle related to Sterile Alpha Motif (SAM) domains. The PNT domain mediates key protein–protein interactions and serves as a docking site for signaling molecules, thereby influencing ERG’s transcriptional activity and function in cancer (26, 29).

In this study, we performed a domain-focused differential scanning fluorimetry (DSF) screen to identify small molecules that bind the structured PNT domain. We identified a PNT-domain ligand, PBITE-1 (PNT-Binding Inhibitor of the Transcription factor ERG), that selectively binds and inhibits ERG function in prostate cancer cells. PBITE-1 induces apoptosis, reduces invasion of ERG-positive cell lines, and suppresses transcription of ERG target genes. Using both cell lines and organoid models, we show that small molecule inhibition of ERG substantially impairs growth of ERG-positive cells and organoids. Interestingly, PBITE-1 treatment significantly induced apoptosis in VCaP xenograft models. These findings establish ERG as a druggable transcription factor and provide the foundation for developing next-generation ERG-targeted inhibitors or PROTAC degraders.

## Results

### ERG Dependency in the VCaP Metastatic Prostate Cancer Cell Line.

To establish the biological rationale for developing ERG-directed therapeutic strategies, we first evaluated the requirement of ERG for prostate cancer cell survival. We performed an unbiased analysis using the Cancer Dependency Map (DepMap; https://depmap.org/portal). Gene essentiality in CRISPR-based screens (Project Achilles) is quantified using the Chronos dependency score, where negative values indicate reduced proliferation following gene knockout (30).

**Fig. 1.**
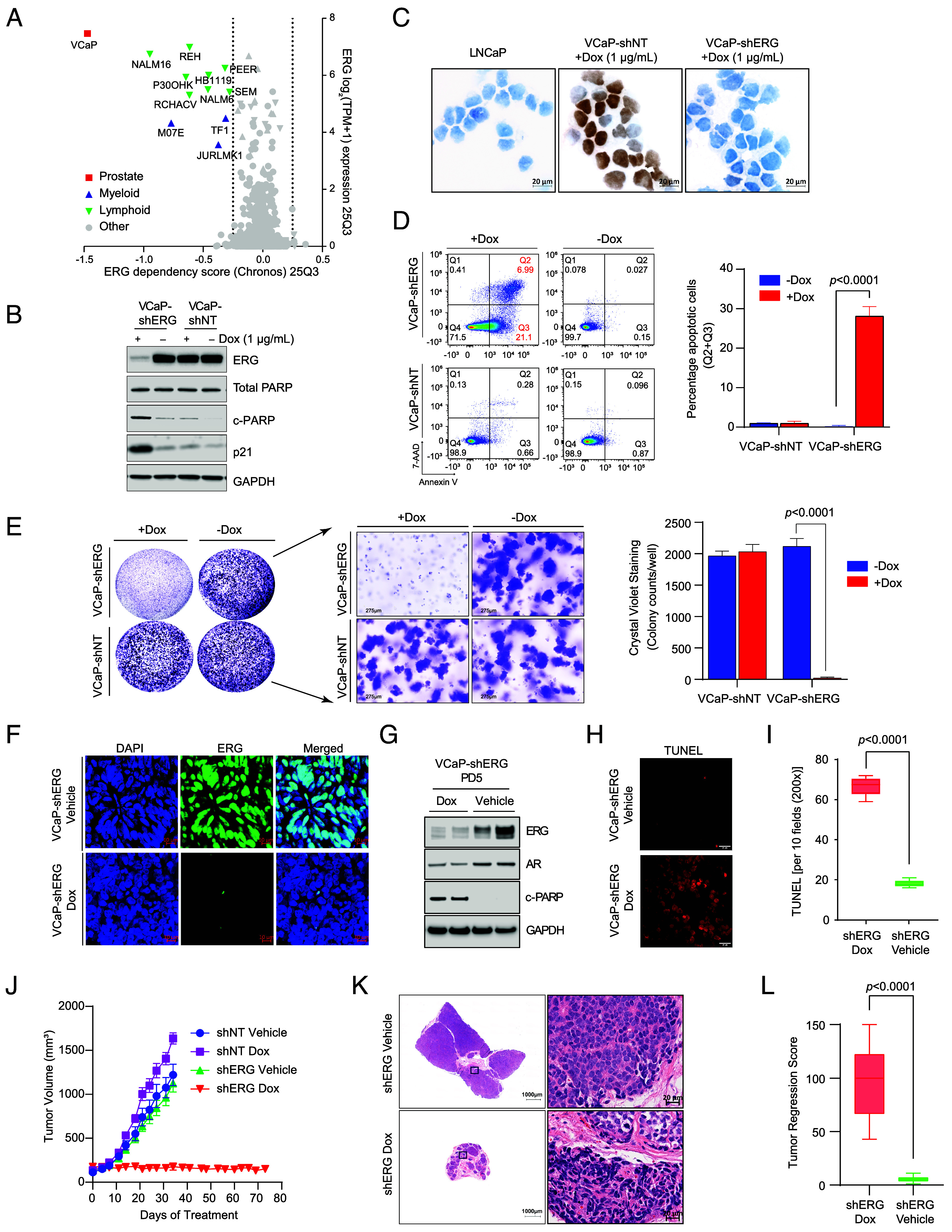
ERG dependency in the VCaP metastatic prostate cancer cell line. (*A*) DepMap analysis reveals dependency of ERG in prostate cancer and hematological malignancies. Dot plot of cancer cell lines shows *ERG* mRNA expression (*y*-axis) versus ERG dependency score (*x*-axis); lower Chronos scores indicate reduced cell proliferation upon gene knockout. (*B*) Immunoblot of ERG protein levels in the inducible VCaP-shRNA model. VCaP cells expressing nontargeting (shNT) or ERG-targeting (shERG) shRNA were treated with 1 µg/mL doxycycline for 3 d. (*C*) Immunocytochemistry (ICC) staining of ERG in doxycycline-induced VCaP-shERG cells. LNCaP cells served as a negative control (Scale bar, 20 μm.) (*D*) Apoptosis analysis by Annexin V/7-AAD flow cytometry after 3 d of ERG knockdown in the VCaP-shERG model. The histogram summarizes percentage of apoptotic cells (Q2 + Q3). (*E*) Colony formation assay of VCaP-shERG cells treated with doxycycline for 5 wk. Histogram shows colony counts for each condition (triplicates). (*F*) Immunofluorescence staining of ERG in VCaP-shRNA xenograft tumors after 5 d of doxycycline treatment (Scale bar, 10 μm.) (*G*) Immunoblot analysis of ERG and the indicated proteins in VCaP-shRNA xenograft tumors collected in the PD5 study, with GAPDH as a loading control. (*H* and *I*) Representative images of TUNEL staining in tumor sections from the PD5 study (*H*) and quantification data (*I*). (*J*) Average tumor volume for VCaP-shRNA xenografts with or without doxycycline chow in SCID mice, measured twice weekly using calipers. Data were analyzed by the two-tailed unpaired *t* test and are presented as mean ± SEM (n = 10). (*K*) Representative H&E staining for VCaP-shERG tumors from (*J*) (scale, 1,000 μm); full images shown in *SI Appendix*, Fig. S1*E*. Black boxes indicate the regions shown at higher magnification (scale, 50 μm). (*L*) Tumor regression score in VCaP-shERG tumors with or without doxycycline treatment in SCID mice.

Using the publicly available DepMap dataset (Public 25Q3), we examined ERG dependency across more than 1,770 cancer cell lines. Plotting *ERG* mRNA expression against ERG Chronos scores revealed that VCaP—the only AR-positive (AR^+^) prostate cancer cell line harboring the *TMPRSS2:ERG* fusion—and a subset of hematologic cancer cell lines with high ERG expression, exhibited strong negative dependency scores (Fig. 1*A*). These findings suggested that ERG is essential for proliferation in these contexts. On this basis, we selected VCaP cells and the ERG-dependent acute lymphoblastic leukemia cell lines REH and NALM16 for experimental validation.

We first generated a doxycycline-inducible shRNA system, where VCaP cells were engineered to stably express either nontargeting (shNT) or ERG-targeting (shERG) shRNAs under the control of the pLKO-Tet-on-puro system. Upon doxycycline induction (1 μg/mL), ERG protein levels were markedly reduced in VCaP-shERG cells, while they were unchanged in shNT controls (Fig. 1*B*). Immunoblot analyses showed a pronounced increase in cleaved PARP (c-PARP) upon ERG knockdown. This was accompanied by robust upregulation of p21, indicative of cell cycle arrest (Fig. 1*B*). Consistently, flow cytometry analysis showed a decreased S-phase population and cell cycle arrest following 48 h of doxycycline treatment (*SI Appendix*, Fig. S1*A*). These effects were not observed in shNT control cells under identical conditions. ERG loss was further confirmed by immunocytochemistry (ICC) (Fig. 1*C*). In agreement with elevated c-PARP, the loss of ERG significantly increased the level of apoptotic cells as measured by flow cytometry (Fig. 1*D*). Intriguingly, ERG knockdown also severely impaired clonogenic potential, as evidenced by a near complete loss of colony formation (Fig. 1*E*).

We next assessed ERG dependency in vivo by implanting VCaP-shNT or VCaP-shERG cells in CB17SCID male mice and analyzing whether doxycycline treatment attenuated VCaP-shERG xenografted tumor growth. Mice with palpable tumors were randomized to receive either 625 mg/kg doxycycline-containing diet or vehicle control diet. After 5 d of doxycycline treatment (PD5), immunofluorescence staining (Fig. 1*F*), and immunoblotting (Fig. 1*G*) confirmed robust ERG suppression specifically in VCaP-shERG tumors. ERG suppression was accompanied by marked induction of apoptosis, as evidenced by increased cleaved PARP levels and enhanced TUNEL staining (Fig. 1 *G*–*I*). Strikingly, doxycycline treatment resulted in complete suppression of VCaP-shERG tumor growth by day 30 relative to vehicle group, with no recurrence through day 73 under continued doxycycline exposure (Fig. 1*J* and *SI Appendix*, Fig. S1*B*). Tumors harvested at endpoint exhibited sustained ERG suppression and elevated p21 expression (*SI Appendix*, Fig. S1 *C* and *D*), while histopathological analyses revealed significantly increased tumor regression scores compared to vehicle-treated controls (Fig. 1 *K* and *L* and *SI Appendix*, Fig. S1*E*). Importantly, doxycycline treatment had no measurable effect on VCaP-shNT xenografts, confirming that tumor suppression was specifically attributable to ERG silencing.

To further characterize downstream molecular consequences of ERG loss in vivo, tumor tissues from PD5 and endpoint studies were analyzed using sequential multiplex immunofluorescence (Seq-mIF) on the Lunaphore COMET platform (*SI Appendix*, Fig. S1*F*). Quantitative analyses demonstrated significant induction of p21, marked suppression of c-Myc, and substantial reduction in Ki67 expression following ERG knockdown, consistent with broad inhibition of proliferative and oncogenic signaling pathways (*SI Appendix*, Fig. S1*F*).

Collectively, these findings establish that ERG is essential for the survival, proliferation, and tumor maintenance of *TMPRSS2:ERG*-positive prostate cancer cells. The profound apoptotic and antitumor effects observed upon ERG suppression strongly support ERG as an oncogenic driver and validate the *TMPRSS2:ERG* fusion as a compelling therapeutic vulnerability in metastatic prostate cancer.

### Identification of F0341, a Small-Molecule Binder of the ERG PNT Domain.

We carried out a domain-focused DSF screen to identify small molecules capable of binding the structured PNT domain (amino acid 109–200, PDB: 1SXE) of ERG. We initiated a screen of a focused library of 1,655 compounds from the Center for Chemical Genomics (CCG) at the University of Michigan, each tested at 200 µM in a single-replicate format. This screening led to the nomination of 72 preliminary hits (*SI Appendix*, Fig. S2*A*) with average melting temperature shifts (ΔT_m_) > 1 °C.

**Fig. 2.**
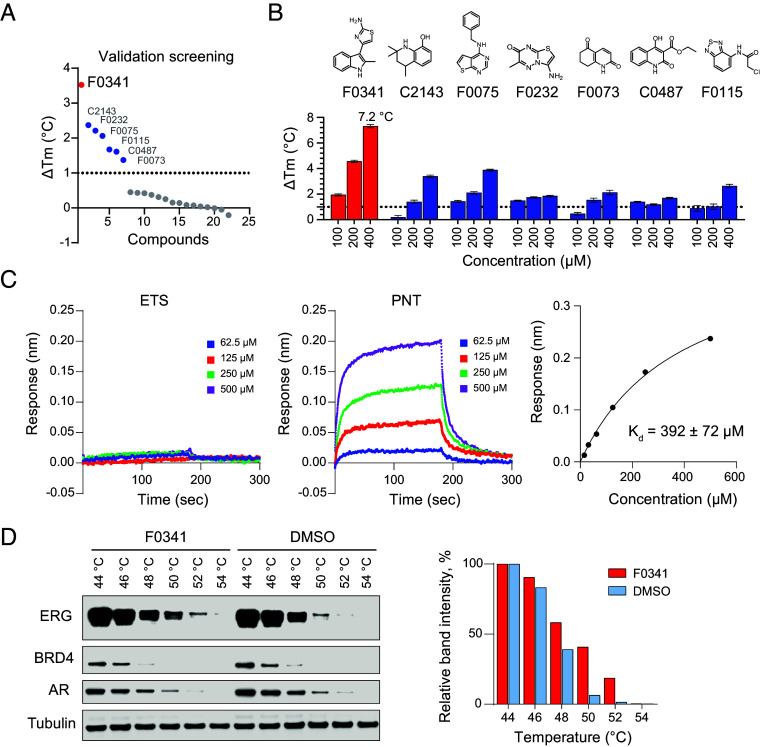
Identification of F0341, a small-molecule binder of the ERG PNT domain. (*A*) DSF validation of 22 commercially purchased hit compounds at 200 µM, seven of which produced a ΔTm > 1 °C (highlighted in blue and red). (*B*) Dose-dependent DSF analysis of the top seven candidate compounds using the ERG PNT domain. Chemical structures of each compound are shown. (*C*) Binding affinity of F0341 for the PNT domain measured by BLI. Biotinylated PNT protein was immobilized on streptavidin biosensors and incubated with the indicated concentrations of F0341; the ETS domain served as a negative control. (*D*) Cellular target engagement of F0341 assessed by CETSA in VCaP cells treated with 400 µM F0341 for 2 h. Soluble protein fractions were analyzed by western blot. BRD4, AR, and tubulin served as negative controls. The bar graph shows ERG band intensities normalized to tubulin.

We then reevaluated the 72 initial hits in quadruplicate using DSF, identifying 22 compounds that produced a reproducible and significant ΔTm > 1 °C (*SI Appendix*, Fig. S2 *B* and *C*). These 22 candidates were subsequently purchased and subjected to dose-dependent DSF assays to assess direct binding to the ERG PNT domain. Seven compounds exhibited robust, concentration-dependent thermal stabilization of the PNT protein (Fig. 2 *A* and *B*). Among them, compound F0341 emerged as the strongest binder, displaying a maximal ΔTm of 7.2 °C at 400 µM (Fig. 2*B*). Based on these properties, F0341 was selected as the hit compound for further study.

To assess the binding specificity of F0341 for the ERG PNT domain, we measured its binding kinetics and affinity using biolayer interferometry. Biotinylated PNT protein was immobilized on streptavidin biosensor tips and exposed to increasing concentrations of F0341 to monitor real-time changes in optical interference. To evaluate potential off-target interactions, the biotinylated ETS domain was used as a negative control. F0341 exhibited dose-dependent binding to the PNT domain with a calculated dissociation constant (*Kd*) of 392 ± 72 µM but showed no detectable binding to the ETS domain (Fig. 2*C*). We also evaluated the binding affinities of the remaining six DSF-validated compounds. Among them, F0341 demonstrated the strongest and most specific interaction with the PNT domain (*SI Appendix*, Fig. S2*D*), supporting its designation as a selective PNT-domain ligand.

To further characterize the interaction between F0341 and the ERG PNT domain, we performed ^1^H-^15^N Heteronuclear Single Quantum Coherence (HSQC) NMR assay of ^15^N-labeled PNT protein titrated with increasing molar ratio of F0341. Overlay of the relevant ^1^H-^15^N spectra revealed robust, dose-dependent chemical shift perturbations (CSPs) in NMR peaks corresponding to residues—R124, V125, I126, and V127 (*SI Appendix*, Fig. S2*E*)—demonstrating that F0341 directly engages the PNT domain.

Finally, we employed the cellular thermal shift assay (CETSA) to assess target engagement and binding specificity in cells (31). In VCaP cells, treatment with F0341 markedly increased the thermal stability of endogenous ERG protein, with soluble ERG detectable at temperatures as high as 52 °C, whereas ERG largely precipitated at 50 °C in DMSO-treated controls (Fig. 2*D*). BRD4, AR, and tubulin served as negative controls, showing no temperature-dependent stabilization. These results validated F0341 as a bona fide small-molecule binder of ERG suitable for further optimization and structure–activity relationship (SAR) studies.

### SAR-Guided Optimization Yields PBITE-1.

**Fig. 3.**
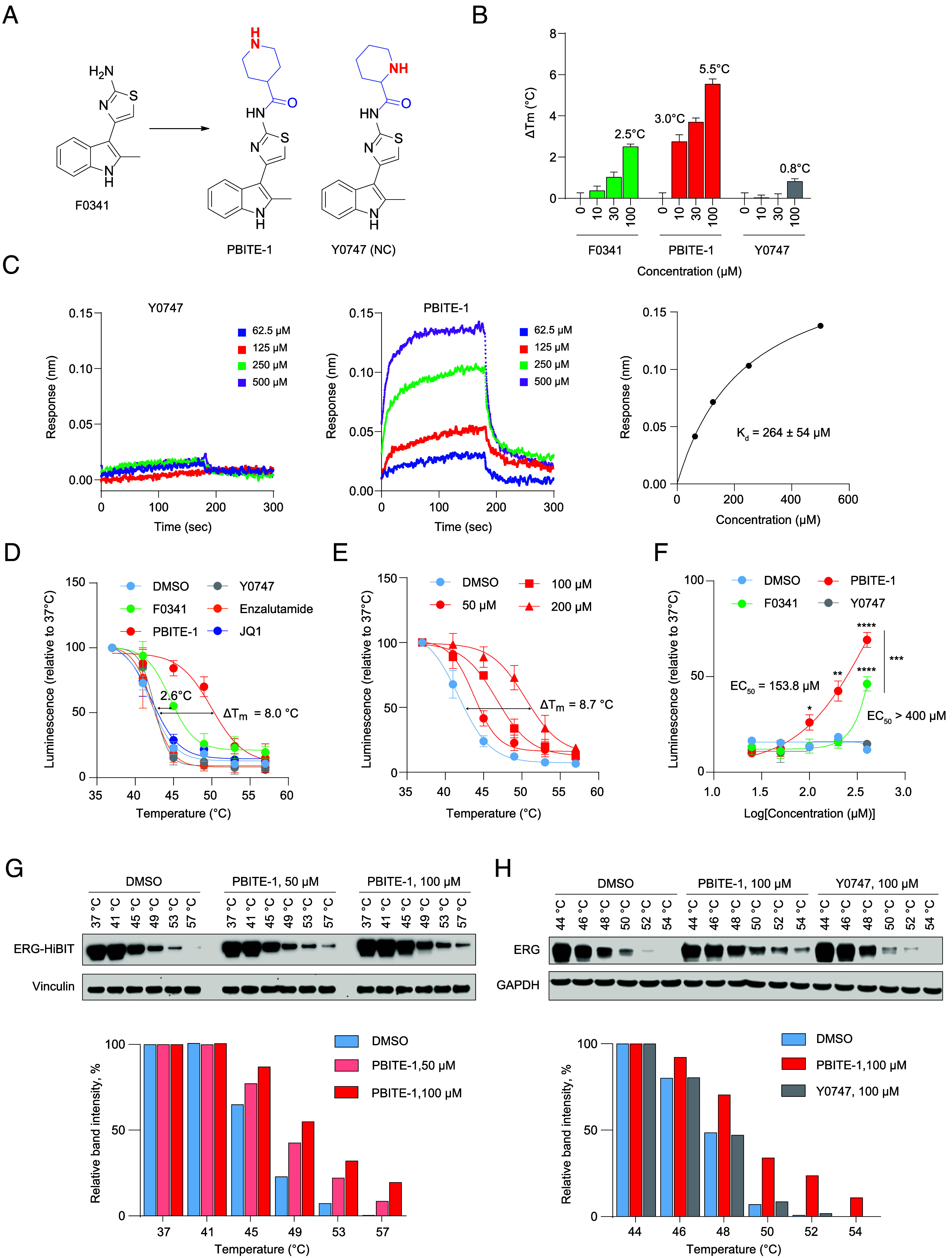
Biochemical and in vitro evaluation of the lead compound PBITE-1. (*A*) Chemical structures of PBITE-1 and Y0747 (negative control, NC), both derived from the hit compound F0341 through SAR optimization. (*B*) Dose-dependent DSF assay of F0341 and PBITE-1; Y0747 was included as a negative control. (*C*) Binding affinity of PBITE-1 for the ERG PNT domain measured by BLI assay, with Y0747 as a negative control. (*D*) Thermal melt profile of full-length ERG protein measured by HiBiT-CETSA. HEK293-ERG-HiBiT cells were treated with DMSO or the indicated compounds (200 µM) for 1 h and heated from 37 to 57 °C. Data represent luminescence intensity as a percentage of the 37 °C treatment ± SD (n = 8 technical replicates). (*E*) Dose–response melt curves of ERG protein treated with PBITE-1 measured by HiBiT-CETSA. (*F*) Isothermal HiBiT-CETSA at 53 °C evaluating ERG stabilization by F0341, PBITE-1, and Y0747 across the indicated concentrations. Data represent mean ± SD (n = 4 technical replicates). Statistical significance versus DMSO was determined by one-way ANOVA: *P < 0.05, **P < 0.01, ***P < 0.001, ****P < 0.0001. (*G* and *H*) CETSA-based target engagement of PBITE-1 in HEK293-ERG-HiBiT cells (*G*) and VCaP cells (*H*) at the indicated concentrations. Y0747 served as a negative control. Vinculin or GAPDH were used as loading controls. Bar graphs show ERG band intensities normalized to loading controls.

To improve binding potency and solubility, we designed and synthesized more than 100 structural analogs based on the F0341 chemical scaffold (*SI Appendix*, Fig. S3). SAR analysis of the piperidine-substituted compounds revealed that the position of the amine group within the piperidine ring was a critical determinant of binding while preserving the improved solubility. Specifically, only the 4′-amine–substituted analog (Y0636) maintained thermal stabilization of the PNT protein, whereas relocating the amine (e.g., Y0747) or modifying the ring size (Y0743, Y0752) abolished binding (*SI Appendix*, Fig. S3). Based on these observations, we selected Y0636, hereafter designated as PBITE-1 (PNT-Binding Inhibitors of Transcription factor ERG), as the lead compound (Fig. 3*A*) for subsequent evaluation.

### Biochemical and In Vitro Evaluation of Lead Compound PBITE-1.

To characterize the binding properties of PBITE-1 toward the ERG PNT domain, we compared its thermal stabilization effects with F0341 and the inactive analog Y0747 (Fig. 3*A*) using DSF assays. PBITE-1 exhibited markedly enhanced potency, stabilizing the PNT domain in a clear dose-dependent manner and achieving a ΔTm of 3 °C at concentrations as low as 10 µM (Fig. 3*B*). Biolayer interferometry further demonstrated that PBITE-1 bound the PNT domain of ERG with an improved *Kd* = 264 ± 54 µM compared to F0341, with no measurable binding detected for the negative control Y0747 (Fig. 3*C*).

To enable quantitative assessment of full-length ERG–ligand interactions in living cells, we developed a NanoLuc-based Cellular Thermal Shift Assay (HiBiT-CETSA) system in HEK293 cells (32–34). In this platform, full-length ERG is fused to an 11–amino acid HiBiT tag (*SI Appendix*, Fig. S4*A*). Upon heating, soluble HiBiT-tagged ERG complements exogenous LgBiT to reconstitute active NanoLuc luciferase, generating luminescence proportional to the amount of thermally stable protein. In contrast, denatured ERG aggregates and cannot undergo complementation, resulting in the loss of signal.

As an assay development control, we first established a HiBiT-tagged BRD4 system and confirmed that the bromodomain inhibitor JQ1 induced robust BRD4 thermal stabilization (ΔTm = 4.9 °C at 200 µM), whereas F0341 and enzalutamide had no effect (*SI Appendix*, Fig. S4*B*). These results validated the sensitivity and dynamic range of the assay. We next evaluated our ERG-binding compounds at 200 µM by generating thermal melt curves across a full temperature gradient in the HiBiT-tagged ERG system (Fig. 3*D*). Relative to DMSO, both PBITE-1 and F0341 increased reconstituted luciferase signal, whereas Y0474, JQ1, and enzalutamide showed no detectable stabilization. Notably, PBITE-1 produced a higher thermal melt curve than F0341, with a ΔT_m_ of 8.0 °C versus 2.6 °C, respectively, indicating stronger ERG binding by PBITE-1. Across a range of concentrations, PBITE-1 exhibited dose-dependent ERG stabilization with a maximal ΔTm of 8.7 °C at 200 µM (Fig. 3*E*).

Last, we determined the EC_50_ of PBITE-1 via isothermal dose response CETSA at 53 °C. Consistent with the melt curve data, PBITE-1 demonstrated a dose-dependent stability curve with EC_50_ = 153.8 µM, whereas F0341 showed a detectable ERG stabilization only at the highest concentration of 400 µM, further highlighting the superior binding capacity of PBITE-1 (Fig. 3*F*). Importantly, the thermal stabilization profiles of HiBiT-tagged ERG protein closely matched that of endogenous ERG protein in VCaP cells, as assessed by Western blot following PBITE-1 treatment (Fig. 3 *G* and *H*). Together, these data provide direct evidence that PBITE-1 binds and stabilizes ERG in cells and exhibits improved target engagement relative to the initial hit compound F0341.

### Mapping PBITE-1 Engagement to the ERG PNT Domain.

**Fig. 4.**
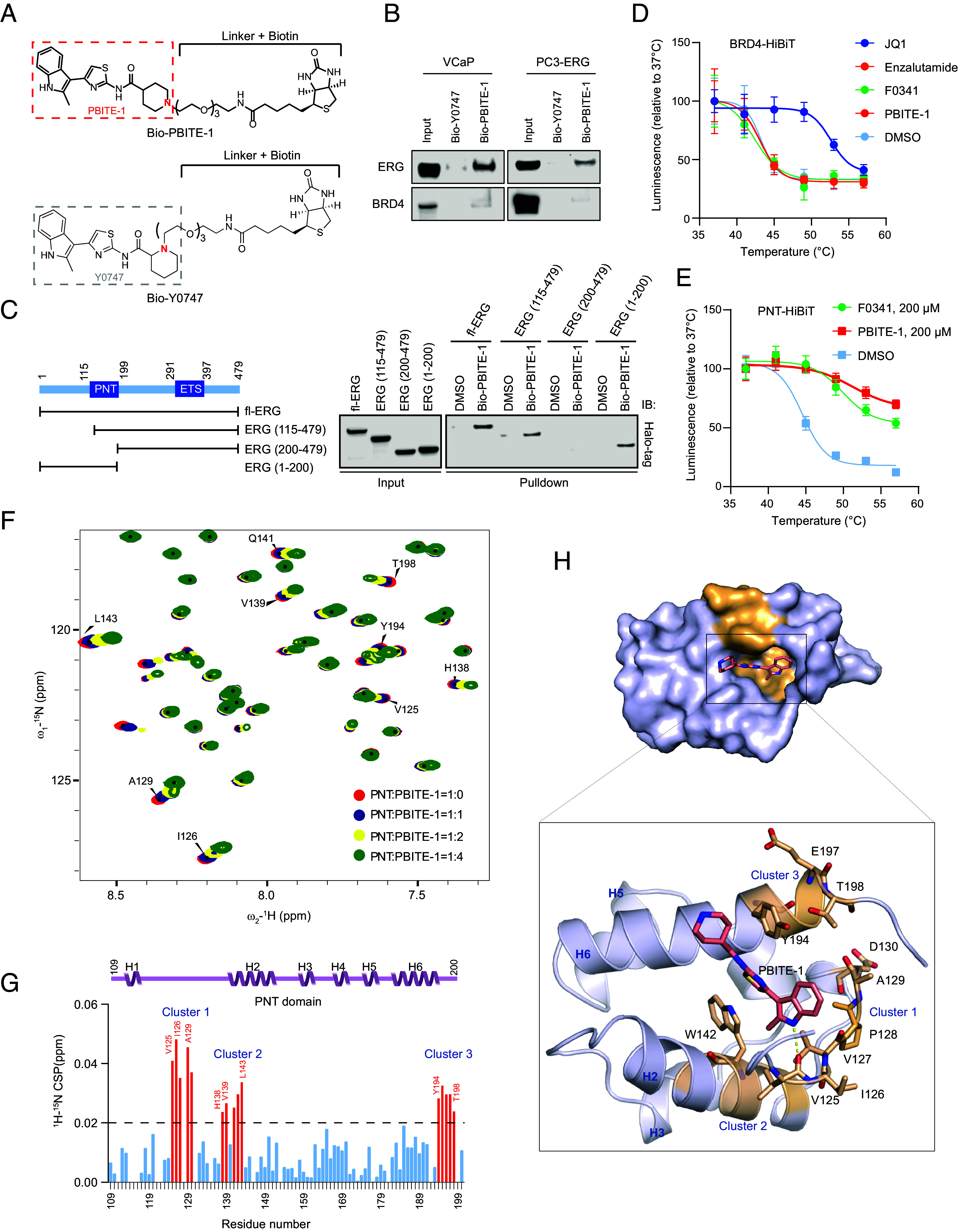
PBITE-1 interacts with a surface of the PNT domain encompassing two α-helices and an adjacent flexible loop. (*A*) Chemical structure of the biotinylated analogs Bio-PBITE-1 and Bio-Y0747. (*B*) Streptavidin pull-down followed by Western blot analysis of VCaP and PC3-ERG cells treated with 10 µM Bio-PBITE-1 and Bio-Y0747 for 2 h. BRD4 served as a negative control. (*C*) Domain mapping of PBITE-1 binding. (*Left*) Schematic of ERG protein fragments used for mapping. (*Right*) Streptavidin pull-down of wheat germ–expressed ERG domain proteins followed by Western blot. Input proteins were loaded as controls. (*D* and *E*) Thermal melt profiles measured by HiBiT-CETSA in HEK293-BRD4-HiBiT (*D*) and HEK293-PNT-HiBiT (*E*) cells. Data represent luminescence intensity as a percentage of the 37 °C treatment ± SD (n = 8 technical replicates). (*F*) ^1^H–^15^N BEST-TROSY spectra showing the backbone chemical shift changes of selected resonances of the PNT-domain protein upon titration of increasing amount of PBITE-1. Colors indicate different PNT:PBITE-1 molar ratios. Representative residues with pronounced CSPs are labeled. (*G*) Amide CSPs of the PNT domain upon addition of PBITE-1 at a 1:4 molar ratio (PNT:PBITE-1). Residues with CSP values exceeding one SD above the mean (dashed line) are highlighted. Significant residues (CSP > 0.02 ppm) cluster into three regions adjacent to helix H1, the intervening loop, and the C-terminal helix H6. The top schematic shows the PNT secondary structure (adapted from PDB 1SXE), comprising helices H1–H6. (*H*) Mapping of CSP clusters onto the PNT 3D structure (PDB 1SXE). The three clusters form a continuous surface that docking predicts to accommodate PBITE-1 (orange). The predicted binding pocket is shown in yellow. Residues with strong perturbations are labeled, and helices H1–H6 correspond to those depicted in (*G*).

To further validate PBITE-1 engagement with full-length ERG in cells, we synthesized Bio-PBITE-1 and Bio-Y0747, biotinylated analogs of PBITE-1 and its inactive counterpart Y0747, respectively (Fig. 4*A*). Pulldown assays followed by Western blot showed that Bio-PBITE-1 efficiently enriched endogenous ERG from VCaP lysates and ERG from PC3 cells overexpressing ERG, whereas BRD4 protein was not detected (Fig. 4*B*), confirming both the direct interaction and binding specificity. The inactive compound Bio-Y0747 did not pull down either ERG or BRD4 proteins (Fig. 4*B*).

To map the PBITE-1-binding region within ERG, we generated a panel of HaloTag-fused ERG constructs, including full-length ERG and three truncation variants (Fig. 4*C*). Pulldown analysis using Bio-PBITE-1 revealed robust binding to full-length ERG, ERG (115 to 479), and ERG (1 to 200), but no detectable interaction with ERG (200 to 479) (Fig. 4*C*).

To validate the specific binding of PBITE-1 to the ERG PNT domain, we employed HiBiT-CETSA assay by conjugating the HiBiT tag to the PNT-domain protein. While neither PBITE-1 nor F0341 showed detectable stabilization on BRD4 protein (Fig. 4*D*), both compounds exhibited a robust thermal stabilization profile on the PNT-domain protein at 200 µM (Fig. 4*E*). Interestingly, PBITE-1 produced a higher thermal melt curve than F0341, further highlighting the superior binding capacity of PBITE-1. Together, these findings demonstrate that the compound-binding activity resides within the N-terminal PNT domain and establishes this domain as the critical region through which PBITE-1 directly engages ERG.

### NMR and Molecule Docking Analyses Defines the PBITE-1 Binding Interface on the ERG PNT Domain.

To define the PBITE-1 binding interface on the ERG PNT domain, we performed ^1^H–^15^N BEST-TROSY NMR titration assays. While the negative analog Y0747 failed to elicit detectable CSPs (*SI Appendix*, Fig. S4*C*), PBITE-1 induced clear, dose-dependent CSPs in a discrete set of amide NMR resonances (Fig. 4*F* and *SI Appendix*, Fig. S4*D*), confirming the direct and specific binding of PBITE-1 for the ERG PNT domain. Further mapping of the significantly perturbed residues onto the secondary structure of PNT showed that the affected sites cluster within three regions: the flexible loop (residues 125–127, 129, 130), helix H2 (residues 138, 139, 141–143), and C-terminal helix H6 (residues 194–198) (Fig. 4*G*). These PBITE-1–responsive clusters form a continuous surface on the PNT 3D structure (PDB ID: 1SXE), suggesting the presence of a coherent binding pocket (Fig. 4*H*). Notably, this pattern mirrors our observations with the parent compound F0341, which also induced prominent CSPs in the consecutive residues 124–127 (*SI Appendix*, Fig. S2*E*).

We next modeled the interaction between PBITE-1 and the PNT domain using the significantly perturbed residues to define the putative binding interface. The complex structure model indicated that the 2-methyl-1*H*-indole moiety of PBITE-1 inserted into a pocket formed by V125, I126, V127, A129, and D130 residues of PNT (cluster 1), while the piperidine moiety projected toward the solvent, positioned adjacent to cluster 2 and cluster 3 (Fig. 4*H*). These features correlate with the high tolerance for chemical modification observed during SAR optimization (*SI Appendix*, Fig. S3). Together, these results demonstrate that PBITE-1 directly binds the ERG PNT domain through a structurally coherent interface spanning helix H2, H6, and the adjacent flexible regions of PNT.

### PBITE-1 Inhibits ERG-Mediated Oncogenic Activities.

**Fig. 5.**
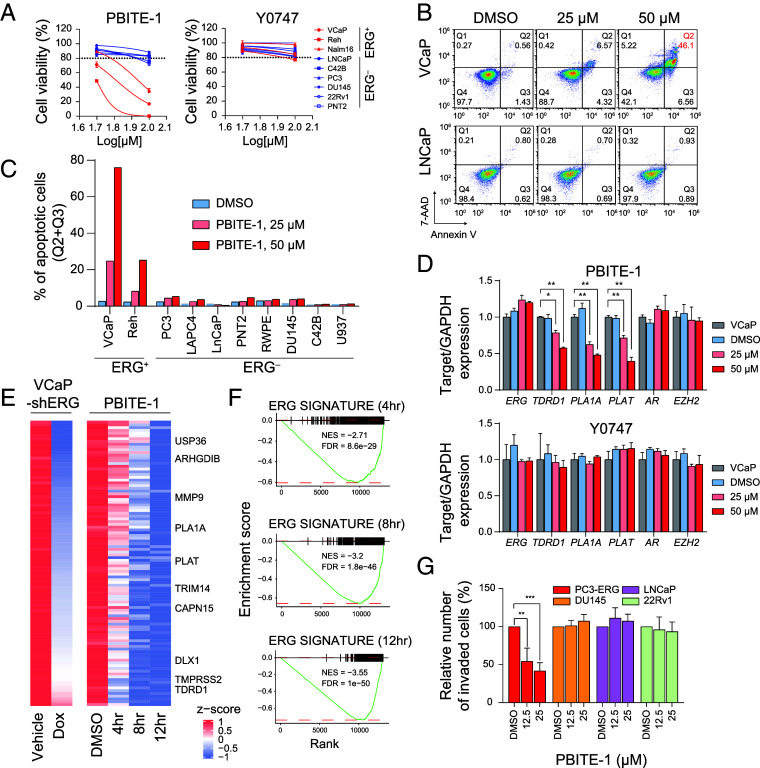
PBITE-1 inhibits ERG-mediated oncogenic activities. (*A*) Cell viability of ERG^+^ and ERG^−^ cells after 24-h treatment with PBITE-1 or Y0747 at the indicated concentrations. Data shown as mean ± SD from three independent experiments. (*B*) Representative apoptosis analysis in VCaP (ERG^+^) and LNCaP (ERG^−^) cells by flow cytometry using Annexin V/7-AAD staining after 24-h treatment with PBITE-1. (*C*) Quantification of apoptotic cells from (*B*). Apoptotic percentages were calculated as the sum of Q2 and Q3 quadrants. (*D*) Relative expression of ERG target genes in VCaP cells after 8-h treatment with PBITE-1 or Y0747, measured by qPCR. Data represent mean ± SD of three technical replicates. (*E*) Comparative heatmap from RNA-seq analysis of ERG-regulated genes identified by shERG knockdown in VCaP cells, comparing gene expression changes induced by ERG knockdown and PBITE-1 treatment at 4, 8, and 12 h. (*F*) GSEA of fold change rank-ordered genes from PBITE-1-treated vs. vehicle-treated VCaP cells at the indicated time points. The gene set was defined by genes significantly downregulated following ERG knockdown by shERG. NES, normalized enrichment score; FDR, false discovery rate; n = 3 biological replicates. Statistical significance was assessed using the GSEA enrichment test. (*G*) Quantification of invaded cells from Boyden chamber transwell invasion assays. Data represent mean ± SD of three technical replicates. Statistical significance was determined by one-way ANOVA: *P < 0.05, **P < 0.01, ***P < 0.001, ****P < 0.0001.

We previously demonstrated that genetic knockdown of ERG suppresses the growth of ERG-positive prostate cancer and ERG-driven hematological malignancies (Fig. 1). To determine whether PBITE-1 functionally phenocopies these effects through direct pharmacologic inhibition of ERG, we first evaluated its impact on cell viability across a panel of ERG-positive (ERG^+^) and ERG-negative (ERG^−^) cell lines. PBITE-1 selectively inhibited proliferation in all ERG^+^ prostate cancer and leukemia cell lines, whereas ERG^−^ cell lines remained largely unaffected (Fig. 5*A*). The inactive analog Y0747 showed no activity in any tested model.

Because *TMPRSS2:ERG* expression is modulated by AR signaling, we compared PBITE-1 with AR antagonists in VCaP cells. PBITE-1 potently suppressed VCaP viability with an IC_50_ of 80.1 µM, whereas enzalutamide and bicalutamide showed minimal effects under the same conditions (*SI Appendix*, Fig. S5*A*).

Consistent with its antiproliferative effects, PBITE-1 induced dose-dependent apoptosis selectively in ERG^+^ cells, as assessed by flow cytometry and PARP cleavage, while ERG^−^ models showed minimal response (Fig. 5 *B* and *C* and *SI Appendix*, Fig. S5 *B*, *C*, and *D*). Y0747 again showed no activity.

Given ERG’s role as a transcription factor, we next examined ERG-mediated transcriptional programs. PBITE-1 significantly suppressed ERG target genes, including *TDRD1*, *PLAT*, and *PLA1A*, in ERG^+^ VCaP cells but not in ERG^−^ models (Fig. 5*D* and *SI Appendix*, Fig. S5*E*). RNA-seq analysis demonstrated substantial overlap between genes downregulated by PBITE-1 treatment and ERG knockdown, with increasing similarity over time (*SI Appendix*, Fig. S5*F*). GSEA further showed suppression of ERG-dependent transcriptional signatures and pathways associated with MYC targets and cell-cycle progression (Fig. 5 *E* and *F* and *SI Appendix*, Fig. S5 *G* and *H*).

We next examined whether PBITE-1 could inhibit ERG-driven cellular invasion using Boyden chamber assays. We previously developed an isogenic PC3 model stably overexpressing ERG that is highly invasive compared to parental PC3 cells (15). PBITE-1 significantly reduced ERG-induced invasion of PC3-ERG cells in a dose-dependent manner, while having no measurable effect on ERG^−^ cell lines. Y0747 showed no inhibitory activity in either model (Fig. 5*G* and *SI Appendix*, Fig. S5 *I* and *J*). Collectively, these findings demonstrate that PBITE-1 selectively suppresses ERG-dependent oncogenic phenotypes across multiple cellular contexts.

### PBITE-1 Selectively Suppresses the Growth of ERG-Positive Prostate Organoids.

**Fig. 6.**
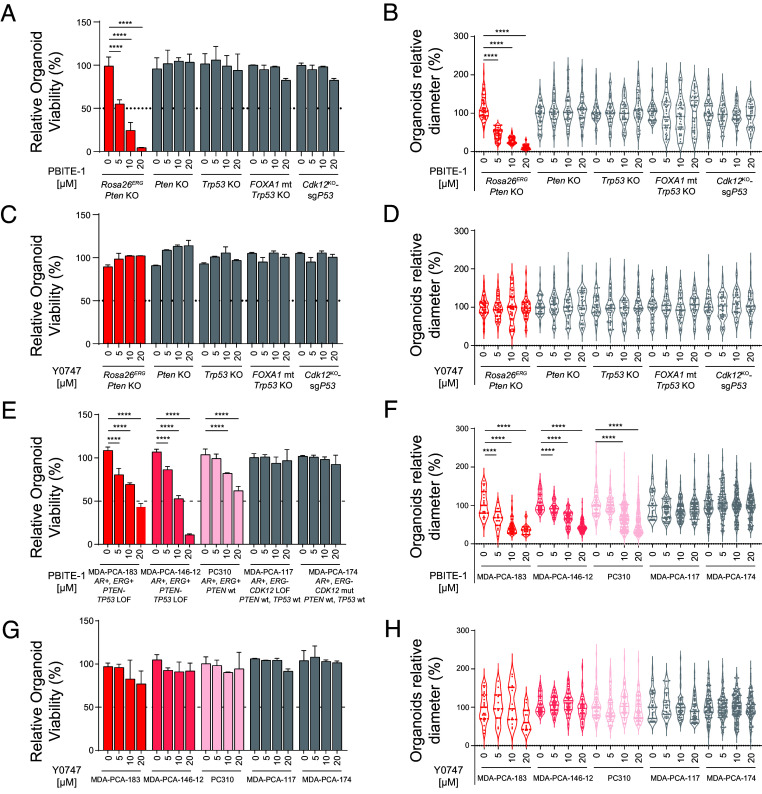
PBITE-1 suppresses growth of ERG-positive organoids. (*A*–*D*) Cell viability of mouse organoids measured by CellTiter Glo 3D (*A* and *C*) and quantification of organoid diameters (*B* and *D*) following treatment with PBITE-1 and Y0747 at the indicated doses. (*E*–*H*) Cell viability of human organoids measure by CellTiter Glo 3D (*E* and *G*) and quantification of organoid diameters (*F* and *H*) after treatment with PBITE-1 and Y0747 at the indicated doses. Statistical significance was assessed by one-way ANOVA: *P < 0.05, **P < 0.01, ***P < 0.001, ****P < 0.0001.

To evaluate the biological consequences of ERG engagement by PBITE-1 in physiologically relevant systems, we leveraged a panel of well-characterized mouse (35–37) and human prostate cancer organoid models. In mouse-derived organoids, PBITE-1 selectively inhibited the proliferation of ERG^+^ organoids in a dose-dependent manner, while ERG^−^ organoids were unaffected (Fig. 6*A*). Quantification of the diameters of organoid spheres revealed a significant, dose-dependent reduction in the *Rosa26^ERG^/Pten* KO model, whereas no changes in all ERG^−^ organoids were observed (Fig. 6*B* and *SI Appendix*, Fig. S6*A*). The negative control compound Y0747 showed no activity across all models (Fig. 6 *C* and *D*).

To extend these observations to human disease models, we examined the effects of PBITE-1 in a panel of patient-derived human prostate cancer organoids (38, 39). PBITE-1 treatment resulted in a dose-dependent reduction in viability and organoid growth in ERG^+^ human lines. In contrast, ERG^−^ human organoids exhibited minimal sensitivity across the same concentration range (Fig. 6*E*). Quantification of organoid diameter further confirmed selective growth inhibition in ERG^+^ contexts (Fig. 6*F* and *SI Appendix*, Fig. S6*B*), while treatment with Y0747 again produced no significant effects across all models (Fig. 6 *G* and *H*).

Altogether, these data demonstrate that PBITE-1 selectively suppresses the growth and survival of ERG-driven prostate cancer organoids across both mouse and human systems. Rather than reflecting broad cytotoxicity, these effects are tightly linked to ERG status, reinforcing the concept of ERG dependency in three-dimensional models that recapitulate key features of tumor architecture and heterogeneity.

### PBITE-1 Induces Cellular Apoptosis in the VCaP Xenograft Model In Vivo.

**Fig. 7.**
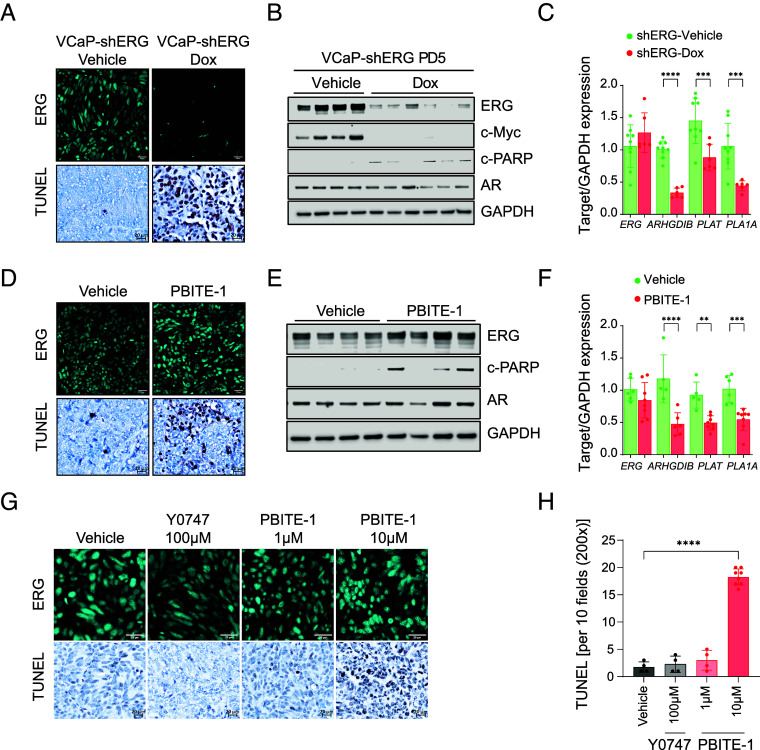
PBITE-1 treatment induces cellular apoptosis in VCaP xenograft models. (*A*) Representative immunofluorescence staining of ERG and TUNEL in tumor sections from the PD5 VCaP-shERG xenograft model. (*B*) Immunoblot analysis of ERG, c-Myc, cleaved PARP, and AR protein levels in xenograft tumors from (*A*). GAPDH is the loading control. (*C*) Relative expression of ERG target genes in VCaP-shERG xenograft tumors in (*A*), assessed by quantitative PCR. Data represent mean ± SD of three technical replicates. (*D*) Representative immunofluorescence of ERG and TUNEL in tumor sections from the PD5 VCaP xenograft model following daily intratumoral treatment with 100 µM PBITE-1. (*E*) Immunoblot analysis of ERG, cleaved PARP, and AR protein levels in xenograft tumors from (*D*). GAPDH is the loading control. (*F*) Relative expression of ERG target genes in PBITE-1 treated VCaP xenograft tumors in (*D*), assessed by quantitative PCR. Data represent mean ± SD of three technical replicates. (*G*) Representative immunofluorescence staining of ERG and TUNEL in tumor sections from VCaP xenograft tumors treated with the indicated doses of PBITE-1 in the PD5 studies. (*H*) Quantification of TUNEL-positive apoptotic cells from (*G*) across treatment groups. Data are presented as mean ± SD. Statistical significance was assessed by one-way ANOVA: *P < 0.05, **P < 0.01, ***P < 0.001, ****P < 0.0001. (Scale bars, 20 µm.)

To evaluate the in vivo pharmacodynamic effects of PBITE-1, VCaP xenograft-bearing mice were treated daily with PBITE-1 for five consecutive days, and tumors were analyzed for molecular and histologic changes. Consistent with prior studies, ERG knockdown reduced ERG protein expression and induced apoptosis, as demonstrated by cleaved PARP and TUNEL staining (Fig. 7 *A* and *B*); ERG-target genes were also significantly downregulated with ERG knockdown (Fig. 7*C*). PBITE-1 treatment did not alter ERG protein abundance, consistent with its role as a functional inhibitor rather than a degrader, but strongly induced tumor cell apoptosis and suppressed ERG-regulated transcriptional targets (Fig. 7 *D*, *E*, and *F*). Histologic analyses further supported effective ERG pathway inhibition (*SI Appendix*, Fig. S7 *A* and *B*).

To determine whether lower pharmacologic doses of PBITE-1 could similarly inhibit ERG function in vivo, we conducted additional VCaP xenograft studies using a dose range of 1 µM, 10 µM, and 50 µM. Notably, significant induction of apoptosis was observed at 10 µM, indicating effective in vivo target engagement at substantially reduced concentrations (Fig. 7 *G* and *H*). RNA-ISH analyses further showed suppression of ERG target genes without changes in *ERG* or *AR* transcript levels (*SI Appendix*, Fig. S7 *C* and *D*). Collectively, these findings demonstrate that PBITE-1 achieves functional ERG inhibition in vivo through suppression of ERG-dependent transcriptional programs and induction of tumor-selective apoptosis.

## Discussion

The ERG transcription factor functions as an oncogenic driver in prostate cancer and other malignancies (40–43). Although several strategies have been explored to modulate ERG levels or activity in prostate cancer (44–49), effective pharmacological approaches that directly target the ERG protein have remained limited, in part due to the longstanding perception that ERG lacks tractable ligand-binding surfaces.

In this study, we described a previously unrecognized ligandable pocket within the ERG PNT domain and presented a proof-of-concept strategy for its pharmacologic targeting. Through a domain-focused small molecule screen, we identified an initial hit, F0341, which selectively stabilizes the structured PNT domain. Subsequent SAR optimization yielded PBITE-1, a more soluble and slightly more potent analog that consistently engaged the PNT domain and serves as a chemical probe to interrogate ERG function across biochemical, cellular, and organoid systems.

Functionally, PBITE-1 selectively impaired ERG-dependent phenotypes in prostate cancer and leukemia models. ERG-positive cells treated with PBITE-1 underwent apoptosis, exhibited reduced invasion, and showed downregulation of ERG transcriptional targets, whereas ERG-negative cells were largely unaffected. PBITE-1 also suppressed the growth of ERG-positive organoids, reinforcing ERG dependency in a physiologically relevant system. These results collectively indicate that PBITE-1 engagement of the PNT domain is sufficient to disrupt ERG-driven transcriptional programs, thereby revealing a biologically actionable vulnerability rather than establishing a fully optimized therapeutic agent.

Targeting the androgen receptor (AR) signaling axis has long been the cornerstone of treatment for metastatic prostate cancer (50). However, this study and other prior work have shown that enzalutamide has limited efficacy in VCaP models both in vitro and in vivo (7, 51–57), reflecting the ERG-driven biology of *TMPRSS2:ERG*–positive prostate cancer. This limited response is attributable, at least in part, to the marked AR amplification in VCaP cells (52, 58–60), a common mechanism of castration resistance observed in patient tumors and a known contributor to resistance to AR-directed therapies.

In contrast, ERG functions as a truncal oncogene in a major subset of prostate cancers, establishing a state of oncogene addiction in which tumor cell survival becomes critically dependent on sustained ERG activity. Accordingly, genetic or pharmacologic suppression of ERG—through shERG or PBITE-1 in this study—induces apoptotic cell death in ERG+ models. This contrasts with AR inhibition, which more commonly induces cell-cycle arrest because AR signaling also plays essential roles in normal prostate epithelial physiology (61). These biological distinctions help contextualize the therapeutic relevance of ERG inhibition relative to established AR-directed strategies.

A key finding of this work is that the ERG PNT domain contains a structurally coherent and pharmacologically accessible ligand-binding interface. NMR CSP mapping revealed that PBITE-1 engages a continuous surface spanning the flexible loop, helix H2, and C-terminal helix H6, collectively defining a coherent ligand-binding pocket within the PNT domain. Docking studies further supported the existence of an integrated binding surface capable of accommodating PBITE-1. Binding is selective to PNT domain-containing ETS family members, including ERG and FLI1, and is unlikely to extend to ETS proteins lacking a PNT domain, such as ETV1, ETV4, and ETV5. Collectively, these results provide direct evidence that the ERG PNT domain harbors a defined ligandable pocket, addressing a longstanding barrier to drugging ERG and, more broadly, other ETS transcription factors. Recent work by Parabilis Medicines independently supports the druggability of the ERG PNT domain. Their stapled-peptide Helicon degrader reportedly binds the PNT domain and induces ERG degradation in vitro and in vivo (https://parabilismed.com/news-events/), reinforcing the concept that the PNT domain represents a therapeutically relevant binding site.

The identification of a discrete PNT-domain binding pocket has important implications for therapeutic development. Because PBITE-1 binds a solvent-exposed surface and stabilizes the domain, it may provide a suitable scaffold for future degrader strategies and medicinal chemistry optimization. More broadly, this work supports a generalizable approach for targeting structurally compact domains within transcription factors. By integrating focused screening with structural and mechanistic validation, we demonstrate that transcription factors long considered undruggable may harbor druggable pockets capable of perturbing oncogenic function. This framework may be extended to other ETS family members as well as to additional transcription factor networks implicated in cancer.

In summary, we define the ERG PNT domain as a ligandable interface and present PBITE-1 as a first-in-class small molecule that directly binds this domain and disrupts ERG-dependent oncogenic activity. Rather than presenting a fully optimized inhibitor, this work defines ERG ligandability and reveals a tractable biological vulnerability. These results lay the foundation for development of ERG-targeted inhibitors and degraders and advance efforts to therapeutically target transcription factor–driven malignancies.

## Methods

### Differential Scanning Fluorimetry.

Thermal stability assays were performed as previously described (62). Each reaction (10 µL) contained 4 µg of purified PNT protein and 10× SYPRO Orange dye (Thermo Fisher Scientific; catalog #: S6650) in a buffer consisting of 20 mM HEPES (pH 7.5), 150 mM NaCl, 1 mM DTT, and 5% glycerol. Proteins were incubated with test compounds or vehicle controls at the indicated concentrations prior to fluorescence measurement on a QuantStudio 6 Pro Real-Time PCR instrument (Applied Biosystems). The temperature was increased from 25 °C to 95 °C at a rate of 0.02 °C/s, and fluorescence signals were continuously recorded. Melting temperatures (T_m_) were determined from the inflection point of the fitted Boltzmann curve using Protein Thermal Shift Software (Thermo Fisher Scientific), and ΔT_m_ values were reported as mean ± SD from seven biological replicates.

### NanoLuc-CETSA.

HEK293-ERG-HiBiT cells were harvested and resuspended in Opti-MEM medium (Gibco; catalog #:31985062) at a density of 0.2 × 10^6^ cells/mL. Cells were treated with test compounds at the indicated concentrations or vehicle control, aliquoted into PCR tubes (50 µL per tube) and incubated for 1 h at 37 °C with 5% CO_2_. The tubes were then heated for 3 min at a series of designated temperatures using a Veriti Thermal Cycler (Applied Biosystems). After cooling to room temperature, cells were equally transferred into four replicate wells of a 384-well plate. Luminescence signals were measured using the Nano-Glo® HiBiT Lytic Detection System Kit (Promega; catalog #: N3040) according to the manufacturer’s instructions on a Tecan plate reader. Data were analyzed using GraphPad Prism 10 software.

### Organoid Growth and Viability Study.

Prostate cancer patient-derived xenograft organoids were generated previously and generously provided by Nora M Navone (63). Both mouse prostate organoids and patient-derived xenograft organoids were established and maintained following previously described protocols (37). Prior to plating for viability study, organoids were digested with TrypLE for 10 min. A total of 500 cells were plated in 10 µL Matrigel in a 96-well plate. Cell viability was measured after 7 d of treatment according to the CellTiter-Glo 3D Cell Viability Assay Kit (Promega G9683). Organoid sphere diameters were quantified with ImageJ software.

### In Vivo VCaP Experiment.

All animal procedures described were approved by the Institutional Animal Care and Use Committee at the University of Michigan. Mice were housed in a specific pathogen-free animal facility. Eight-week-old CB17SCID male mice were implanted subcutaneously with 3 × 10^6^ VCaP-shERG and VCaP-shNT cells. When tumors reached approximately 150 to 250 mm^3^, animals were randomized to receive either a 625 mg/kg doxycycline-containing diet (Envigo TD.09651) or a control diet. Tumor size was measured twice weekly for 4 to 5 wk. Tumors were harvested at the end of the study for biochemical analysis.

### Statistical Analysis.

Data points were acquired with biological replicate samples. Data were analyzed and plotted using GraphPad Prism 10. Data were presented as means ± SD or ±SEM, as stated in the figure legends. Statistical significance was determined with a *P*-value less than 0.05, unless otherwise stated.

## Supplementary Material

Appendix 01 (PDF)

## Data Availability

All sequencing data generated in this manuscript have been deposited in GEO (GSE331048) (64). Other data are included in the article and/or *SI Appendix*.
