# Supplementary material for: A ligandable PNT domain establishes ERG as a directly targetable oncogenic driver in prostate cancer
Source: Proc Natl Acad Sci U S A. 2026 Jul 7;123(28):e2537437123. doi: 10.1073/pnas.2537437123 (PMC13367891; doi:10.1073/pnas.2537437123)
Supplement: Supplementary file 1 — Appendix 01 (PDF) [file pnas.2537437123.sapp.pdf]

PNAS

Supporting Information for

A ligandable PNT domain establishes ERG as a directly targetable oncogenic driver in prostate cancer

Xiaoju Wang[a,b,c,1], Wenyan Liu[a,b,1], Jiehao Yang[d,1], Jean Ching-Yi Tien[a,b], Yu Chang[a], Rahul Mannan[a,b], Somnath Mahapatra[a,b], Yang Zhou[e], Lihao Gan[d], Xuhong Cao[a,b,c], Jiayi Zhou[a,b], Yuping Zhang[a,b], Sharpkate Shaker[a,b], Yichao Huang[d], Hang Qiao[d], Rudana Hamadeh[a], Grafton Ervine[a], Cynthia Wang[a], Fengyun Su[a,b], Rui Wang[a,b], Lanbo Xiao[a,b], Raghunath Ranga Sudharshan[f], Arvind Rao[a,f,g], Zaneta Nikolovska-Coleska[b,c,h], Cole Stephens[h], Lifeng Pan[d], James J. Chou[d], Debashish Sahu[i], Jeanne Stuckey[c,j], Zhen Wang[d], Ke Ding[d,2], and Arul M. Chinnaiyan[a,b,c,k,l,2]

[a] Michigan Center for Translational Pathology, University of Michigan, Ann Arbor, MI 48109
[b] Department of Pathology, University of Michigan, Ann Arbor, MI 48109
[c] Rogel Cancer Center, University of Michigan, Ann Arbor, MI 48109
[d] State Key Laboratory of Chemical Biology, Shanghai Institute of Organic Chemistry, Chinese Academy of Sciences, Shanghai 200032, People's Republic of China
[e] International Cooperative Laboratory of Traditional Chinese Medicine Modernization and Innovative Drug Discovery of Chinese Ministry of Education, Guangzhou City Key Laboratory of Precision Chemical Drug Development, College of Pharmacy, Jinan University, Guangzhou 511400, People's Republic of China
[f] Department of Computational Medicine and Bioinformatics, University of Michigan, Ann Arbor, MI 48109
[g] Department of Radiation Oncology, University of Michigan, Ann Arbor, MI 48109
[h] Medicinal Chemistry Graduate Program, College of Pharmacy, University of Michigan, Ann Arbor, MI 48109
[i] BioNMR Core Facility, Life Sciences Institute, University of Michigan, Ann Arbor, MI 48109
[j] Life Sciences Institute, University of Michigan, Ann Arbor, MI 48109
[k] HHMI, University of Michigan, Ann Arbor, MI 48109
[l] Department of Urology, University of Michigan, Ann Arbor, MI 48109

[1] X.W., W.L. and J.Y. contributed equally to this work.
[2] To whom correspondence may be addressed.
Ke Ding, Ph.D.
dingk@sioc.ac.cn
Arul M. Chinnaiyan, M.D., Ph.D. (primary contact)
arul@med.umich.edu

This PDF file includes:

Supplementary Methods
Supplementary Tables 1-2
Figures S1 to S7
Compounds synthesis and characterization
References

Supplementary Methods

Cell lines

Cell lines were obtained from the American Type Culture Collection (ATCC). All lines were authenticated by genotyping analysis performed at the University of Michigan Sequencing Core and were routinely tested for Mycoplasma contamination. VCaP cells were cultured in DMEM supplemented with GlutaMAX (Gibco), 10% heat-inactivated fetal bovine serum (FBS; Hyclone) and 1% penicillin–streptomycin (P/S). PC3 and PC3-ERG overexpression (OE) cells were maintained in RPMI 1640 medium (Gibco) supplemented with 10% FBS and 1% P/S. 22Rv1 cells were maintained in RPMI 1640 medium (ATCC modification; Gibco) with 10% FBS and 1% P/S. HEK293 and HEK293-ERG-HiBIT cells were cultured in EMEM (ATCC) supplemented with 10% FBS and 1% P/S.

Cell viability

Cells were seeded in 96-well plates and incubated at 37 ℃ with 5% CO_2 overnight. A serial dilution of test compounds was added to the cells. After 24 hours of incubation, cell viability was assessed using the CellTiter-Glo Luminescent Cell Viability Assay (Promega; catalog #: G7572) according to the manufacturer’s instructions. Luminescence signals were measured using a plate reader (Tecan), and

data were analyzed with GraphPad Prism 10 software.

Western blot

Cells were seeded in 6-well plates and incubated at 37 ℃ with 5% CO_2 overnight. Cells were then treated with the indicated concentrations of test compounds for 4–24 hours and lysed in Pierce RIPA buffer (Thermo Fisher Scientific; catalog #: 89900) supplemented with Halt™ protease and phosphatase inhibitor cocktail (Thermo Fisher Scientific; catalog #: 78440). After incubation on ice for 30 minutes, cell lysates were sonicated and centrifuged at 20,000 × g for 20 minutes at 4 ℃ to collect the supernatant. Protein concentrations were determined using the DC Protein Assay Kit (BIO-RAD; catalog #: 5000112). Equal amounts of total protein were loaded on NuPAGE 4–12% Bis-Tris gels (Thermo Fisher Scientific; catalog #:WG1402BOX, WG1403BOX) and transferred onto PVDF (Merck; catalog #: IPVH20200) membranes. Membranes were blocked with 5% nonfat milk for 1 hour at room temperature and incubated overnight at 4 ℃ with primary antibodies. After three washes with TBST buffer (0.1% Tween-20 in TBS), membranes were incubated with HRP-conjugated rabbit or mouse secondary antibodies and visualized using the Odyssey Fc Imager (LI-COR). Antibodies used in this study include anti-ERG (Abcam; catalog #: ab92513), anti-BRD4 (Cell Signaling Technology; catalog #: 13440S), anti-AR (Abcam; catalog # ab133273), anti-HiBIT (Promega; catalog #: N7200), anti-HaloTag (Promega; catalog #: G9211), anti-cleaved PARP (Cell Signaling Technology; catalog #: 9544S), anti-tubulin (Abcam; catalog #: ab184577), anti-GAPDH (Proteintech; catalog #: 10494-1-AP), and anti-vinculin (Cell Signaling Technology; catalog #: 4650S).

RNA extraction and quantitative real-time polymerase chain reaction

VCaP cells were seeded in poly-D-lysine–coated 6-well plates and treated with test compounds for 4 hours. Total RNA was extracted using the miRNeasy Mini Kit (Qiagen; catalog #: 217004) following the manufacturer's instructions, and RNA concentration was determined with a NanoDrop spectrophotometer. cDNA was generated from 1 µg of total RNA using SuperScript III Reverse Transcriptase (Thermo Fisher Scientific; catalog #: 18080044). Quantitative real-time PCR was performed in triplicate using Fast SYBR Green Master Mix (Thermo Fisher Scientific; catalog #: 4385612) on a QuantStudio 6 Pro Real-Time PCR System (Applied Biosystems). mRNA levels were normalized to *ACTB* as an internal control using the ΔCt method and quantified relative to the DMSO control group. Primer sequences were designed and synthesized as the reference (1, 2).

RNA-seq and data analysis

VCaP cells were treated with 50 µM PBITE-1 for 4, 8, and 12 hours, each in three biological replicates. The cells were then subjected to RNA sequencing as previously described (3). Sequencing reads were quantified with Kallisto (v0.46.1), normalized with edgeR (v3.39.6), and analyzed for differential expression using limma-voom (v3.53.10). Gene set enrichment analysis was performed using fgsea (v1.24.0), and data visualization employed tidyverse, gplots, ggplot2, and EnhancedVolcano (v1.15.0) packages in R.

Biolayer interferometry

Super Streptavidin (SSA) biosensors (Sartorius; catalog #: 18-5057) were pre-equilibrated in PBS buffer containing 0.02% Tween-20 and 0.1% BSA. Biotinylated PNT protein was immobilized onto the sensors at room temperature until the binding signal reached a plateau. After immobilization, the sensors were washed in buffer to remove nonspecific binding and subsequently incubated with test compounds at varying concentrations to monitor the association phase, followed by transferring into buffer for dissociation. The binding data were analyzed using Octet Analysis Studio 13.0 (Sartorius). All sensorgrams were reference-subtracted and globally fitted to a 1:1 binding model to calculate the binding affinity (Kd).

Cancer dependency map (DepMap) database analysis

DepMap online tool was used to visualize the data from the Cancer Cell Line Encyclopedia (CCLE) database. DepMap (https://depmap.org/portal/) combines Clustered Regularly Interspaced Short Palindromic Repeats (CRISPR) and RNA interference (RNAi) data and is applied to determine gene dependencies of human genes in numerous cancer cell lines. The gene effect score of individual genes is obtained from screening experiments. The scores appraise the effect size of knocking down or knocking out human genes. A negative score indicates that the cell lines grow slower after knocking down or knocking out of a gene, while a positive score indicates that the cell lines grow faster (4).

Cellular thermal shift assay

Cells were seeded in 10-cm dishes and allowed to attach overnight. Cells were then treated with test compounds or DMSO for 2 hours at 37 ℃, followed by harvesting and washing with PBS. The cell suspension was aliquoted into PCR tubes (50 μL per tube) and heated for 3 minutes at a series of designated temperatures in a Veriti Thermal Cycler (Applied Biosystems). After cooling to room temperature, cells were lysed by three cycles of freeze–thaw using liquid nitrogen. Aggregated proteins were removed by centrifugation at 20,000 × g for 20 minutes at 4 ℃, and the soluble fractions were collected for subsequent Western blot analysis.

Cell invasion

Cells were harvested and washed twice with serum-free medium. After counting, the cell density was adjusted to the desired concentration. Corning BioCoat Matrigel Invasion Chambers (Corning; catalog #: 354480) were pre-hydrated with PBS for 2 hours prior to use. Cells suspended in serum-free medium were seeded into the upper chambers, while complete medium containing 10% FBS was added to the lower chambers as a chemoattractant. Test compounds at the indicated concentrations were added to both the upper and lower chambers. After incubation for 24 hours at 37 ℃ in a humidified 5% CO_2 incubator, cells were washed with PBS and fixed in methanol. Non-invaded cells remaining on the upper membrane surface were carefully removed with a cotton swab. Invaded cells on the underside of the membrane were stained with 0.2% crystal violet (Thermo Fisher Scientific; catalog #: B21932-22) at room temperature for 15 minutes. Images were captured using an inverted microscope, and quantification was performed using ImageJ software.

Cell apoptosis by flow cytometry

Cells were seeded in 6-well plates and incubated at 37 ℃ with 5% CO_2 overnight. Cells were treated with test compounds at the indicated concentrations for 24 hours, harvested, and washed twice with cold Cell Staining Buffer (BioLegend; catalog #: 420201). Cells were then resuspended using the FITC Annexin V Apoptosis Detection Kit with 7-AAD (BioLegend; catalog #: 640922) according to the manufacturer's instructions. Singlet cells were analyzed by flow cytometry (Sony Biotechnology; SH800), and apoptosis rates were quantified using FlowJo software.

NMR spectroscopy

All NMR experiments were performed on an 800-MHz Bruker Avance spectrometer (Bruker BioSpin, Billerica, MA, USA) equipped with a triple-resonance TCI cryogenic probe or an Agilent 800 MHz spectrometer equipped with an actively z gradient shielded triple resonance probe at 298 K. Uniformly ^{15}N-labeled ERG PNT domain was prepared at 400 μM in NMR buffer containing 50 mM sodium phosphate, 50 mM NaCl, 1 mM DTT, pH 6.5, supplemented with 10% D_2O (v/v) for the lock signal. Stock solutions of PBITE-1 and F0341 were prepared in DMSO and titrated into the protein sample at molar ratios of 0:1, 0.5:1, 1:1, 2:1, 4:1, and 8:1 (ligand:protein), keeping the final DMSO concentration below 5**%** to minimize solvent effects.

Two-dimensional ^{1}H–^{15}N BEST-TROSY spectra were acquired at each titration point using 64 scans, 256 complex points in the indirect (^{15}N) dimension, and spectral widths of 15.6 ppm (^{1}H) and 35 ppm

(^{15}N). The interscan delay was set to 0.2s. Spectra were processed with topspin software using sine-bell window functions and zero-filled to double the real points prior to Fourier transformation and converted to sparky format for analysis. Resonance assignments and spectral analysis were performed in NMRFAM-Sparky (5).

Combined chemical shift perturbations (CSPs) were calculated using the equation:

$$\Delta\delta = \sqrt{[(\Delta\delta_H)^2 + (\alpha \cdot \Delta\delta_N)^2]}$$

where $\Delta\delta_H$ and $\Delta\delta_N$ are the chemical shift changes in the ^{1}H and ^{15}N dimensions, respectively, and $\alpha = 0.1$ accounts for the gyromagnetic ratio of the two nuclei (6). Residues exhibiting CSPs greater than the mean plus one standard deviation were considered significantly perturbed and used to define the ligand-binding interface.

Computational modeling

The structure of the ERG PNT domain (PDB ID: 1SXE) was used as the receptor for docking studies. The ligand PBITE-1 and protein were prepared using LigPrep and Protein Preparation Wizard, respectively (Schrödinger, LLC, New York, NY, 2025). The potential binding pocket was defined based on residues exhibiting significant chemical shift perturbations in HSQC experiments. Initial docking was conducted with the Glide module. Docking poses were scored with GlideScore, and top-ranking models consistent with the NMR perturbation pattern were selected as representative binding modes.

Wheat germ cell-free translation

Protein synthesis was performed using a wheat germ cell-free translation system (e.g., WEPRO or TNT Wheat Germ Extract; vendor as appropriate). DNA templates encoding the ERG PNT domain were prepared by in vitro transcription or PCR amplification with T7 promoter sequences according to the manufacturer’s instructions.

Translation reactions were assembled on ice in a total volume of 20–50 µL containing wheat germ extract, amino acid mixture, energy regeneration system, RNase inhibitor, and 50–200 ng of DNA template. Reactions were incubated at 25–26 °C for 1–3 hours. For large-scale synthesis, reaction volumes were increased proportionally, and fed-batch conditions were maintained by periodic supplementation of substrates following the manufacturer’s protocol.

Translated proteins were clarified by centrifugation at 15,000 × g for 15 minutes at 4 °C and subjected to downstream applications. For binding assays, translated PNT proteins were buffer exchanged into assay buffer (20 mM HEPES, pH 7.4, 150 mM NaCl) using desalting columns. Protein yield was quantified by SDS/PAGE with Coomassie staining or by western blot when appropriate. For NMR or DSF studies, translated material was further purified by size-exclusion chromatography to remove low-molecular-weight components derived from the extract.

Pulldown assay

Cell nuclear lysates were prepared in ice-cold lysis buffer supplemented with protease and phosphatase inhibitor cocktail (Thermo Fisher Scientific; catalog #: 78440) using the NE-PER™ Nuclear and Cytoplasmic Extraction Reagents (Thermo Fisher Scientific; catalog #: 78835) according to the

manufacturer's instructions. Clarified lysates were precleared with Pierce NeutrAvidin agarose beads (Thermo Fisher Scientific; catalog #: 29204) for 1 hour at 4 ℃ and incubated with biotinylated compounds or negative controls for an additional 1 hour at room temperature with gentle rotation. Prewashed NeutrAvidin agarose beads were added and incubated with the mixtures overnight at 4 ℃. Beads were collected by centrifugation and washed five times with ice-cold wash buffer. Bound proteins were eluted by boiling in 2× SDS sample buffer and analyzed by western blot.

Sequential multiplex immunofluorescence (Seq-mIF) by COMET on single tissue sample

Five-micron formalin-fixed, paraffin-embedded (FFPE) tissue samples were mounted onto SuperFrost Plus™ glass slides in accordance with Lunaphore's tissue placement guidelines. To ensure proper adhesion of the tissue, the slides were baked at 60°C for 1 hour before staining. Subsequently, the slides were deparaffinized using a standard xylene and graded ethanol series, followed by heat-induced epitope retrieval (HIER) as per the Lunaphore COMET protocol to unmask target epitopes (7). Automated sequential immunofluorescence (seqIF™) staining was performed on the COMET™ instrument (Lunaphore) to enable highly multiplexed protein detection with the target primary antibodies, as detailed in **Supplementary Table 1**. The antibody panel—detailing targets, clones, vendors, and optimal dilutions—was thoroughly optimized before the full run. This process involved cycles of staining, imaging, and elution managed by the COMET Control software through controlled temperature and microfluidic reagent delivery. Each cycle included incubation with unconjugated primary antibodies, followed by species-specific fluorescently conjugated secondary antibodies (e.g., Alexa Fluor 555 and

647), automated image acquisition including DAPI counterstaining, and complete antibody removal via elution. After the final staining cycle, the COMET Control software automatically stitched, aligned, and stacked images to produce a single OME-TIFF file for each sample. During post-processing, autofluorescence background was systematically subtracted using reference images acquired from unstained tissue sections at the beginning of the protocol.

Seq-mIF image Processing and data analysis

The raw data consisted of single-cell protein expression measurements across multiple markers along with associated spatial coordinates. Marker features were grouped into biologically relevant panels for downstream visualization and analysis. Outliers were removed separately for each marker within each annotation group by excluding cells with expression values outside ±2 median absolute deviations (MAD) from the group-specific median. The MAD is a robust measure of dispersion defined as the median of the absolute deviations from the median and is less sensitive to extreme values than the standard deviation. Following outlier removal, expression values were log1p-transformed to stabilize variance and reduce the influence of skewed distributions. Group differences were quantified using Cliff's delta (δ) (8), a non-parametric effect size defined as $\delta = P(X>Y) - P(X<Y)$, for observations X and Y drawn from the two groups, respectively. Vehicle was used as the reference group; thus, positive δ values indicate higher expression in Dox relative to Vehicle, and vice versa. This approach was chosen due to the large sample size (~10^4 cells), where significance testing can overstate minor differences (9). Effect sizes were

interpreted as: |δ| < 0.147 (negligible), 0.147–0.33 (small), 0.33–0.474 (medium), and ⩾ 0.474 (large), with 95% confidence intervals (CI) reported for all estimates.

RNA in situ hybridization

RNA in situ Hybridization (RNA-ISH) was performed on 4-micron FFPE tissue sections using RNAscope 2.5 HD Brown kit (Advanced Cell Diagnostics, Newark, CA) and target probes (**Supplementary Table 2**) with RNA quality accessed using Hs-PPIB probe (positive control) and DapB probe (negative control) as described previously (10, 11). The RNA-ISH data and quantitative scoring in the present study cohort for target probes were performed as described (12). The number of brown punctate dots (each dot corresponding to a single RNA molecule) and clusters per cell was counted; expression level was evaluated according to the RNAscope scoring criteria as follows: score 0 = no staining or fewer than one dot per 10 cells, 1 = one to three dots per cell, 2 = four to nine dots per cell with no/few clusters, 3 = 10-15 dots per cell and <10% dots in clusters, and 4 = >15 dots per cell with >10% dots in clusters.

Immunocytochemistry (ICC)/Immunohistochemistry (IHC)/ Immunofluorescence (IF)

Multiple molecular technologies were employed in this study. All three modalities—immunocytochemistry (ICC), immunohistochemistry (IHC), and immunofluorescence (IF)—were performed on the fully automated Ventana Discovery platform (Roche Diagnostics). IHC and IF were carried out as previously described (13). The primary antibodies used were ERG (Abcam; catalog #ab92513) and p21 (Cell Signaling Technology; catalog #2947S; rabbit monoclonal). Reagents

from Roche-Ventana Medical Systems included CC1 (catalog #06414575001), CC2 (catalog #950-223), Inhibitor (catalog #760-4840), OmniMap anti-rabbit HRP (catalog #760-4311), OmniMap anti-mouse HRP (catalog #760-4310), the Universal DAB Detection Kit (catalog #760-500), and the FITC Kit (catalog #760-232).

While IHC and IF were performed on unstained FFPE tissue sections, ICC was performed on cell pellets prepared from a cell suspension. Briefly, cells were washed, resuspended at a density of 5×10^6 cells/mL, loaded into a cytofunnel assembly with a slide and filter, and centrifuged to deposit cells as a monolayer. The deposited cells were briefly air-dried and fixed in pre-chilled acetone for 5 minutes, followed by air-drying. After fixation, ERG primary antibody staining was performed on the Ventana platform without an antigen-retrieval step. Secondary antibody incubation and chromogen development were carried out according to standard procedures.

Assessment of tumor regression

To evaluate treatment-induced changes in tumor morphology, representative FFPE tumor sections were stained with hematoxylin and eosin (H&E) and analyzed according to the AJCC/CAP tumor regression grading (TRG) parameters (14). The AJCC/CAP TRG system was modified to assess pathological response to ERG depletion by semi-quantitatively evaluating the volume of residual viable tumor cells.

Two pathologists, blinded to treatment groups, independently evaluated nuclear features (pyknosis, karyorrhexis, and karyolysis) and cytoplasmic alterations (eosinophilia and vacuolization/hydropic change) in tumor cells, as well as tumor microenvironmental features, including necrosis,

fibrosis/hyalinization, inflammatory infiltrates, and dystrophic calcification. For each tumor sample, the percentage of epithelial tumor cells exhibiting regression and degenerative changes (Rc) and the percentage of viable tumor cells (Vc) were recorded. The Tumor Regression Score (TRS) was calculated using the formula: TRS = (Rc / Vc) × 100.

Supplementary Table 1. Antibodies used for histological study

Reagent	Source	Catalog No
Rabbit Monoclonal IgG Human ERG [EPR3864] antibody	Abcam	ab92513
Rabbit Monoclonal IgG Human c-MYC [Y69] antibody	Abcam	ab32072
Rabbit Monoclonal IgG Human ki-67 [**BLR021E**] antibody	Abcam	ab243878
Rabbit Monoclonal IgG Human P21 [12D1] antibody	Cell Signaling Technology	2947
Rabbit Monoclonal IgG Human CK18 [DC10] antibody	Cell Signaling Technology	4548
Rabbit Monoclonal IgG Human CD44 [E7K2Y] antibody	Cell Signaling Technology	37259

Supplementary Table 2. Target probes used for RNA-ISH

Reagent	Source	Catalog No
RNAscope Target Probe - Homo sapiens v-ets erythroblastosis virus E26 oncogene homolog (avian) (*ERG*), transcript variant 1, mRNA	Advanced Cell Diagnostics, Inc	604021
RNAscope™ Probe - Hs-*AR* - Homo sapiens androgen receptor (*AR*), transcript variant 1, mRNA	Advanced Cell Diagnostics, Inc	400491
RNAscope™ Probe - Hs-*ARHGDIB*-C1, Homo sapiens Rho GDP dissociation inhibitor beta (ARHGDIB), transcript variant 4, mRNA	Advanced Cell Diagnostics, Inc	1677601
RNAscope™ Probe - Hs-MYC - Homo sapiens v-myc myelocytomatosis viral oncogene homolog (avian) (MYC), mRNA	Advanced Cell Diagnostics, Inc	311761
RNAscope™ Probe - Hs-PLAT-C1Homo sapiens plasminogen activator tissue type (PLAT) transcript variant 1 mRNA	Advanced Cell Diagnostics, Inc	1053701
RNAscope™ Probe - Hs-PLA1A - Homo sapiens phospholipase A1 member A (PLA1A) transcript variant 1 mRNA	Advanced Cell Diagnostics, Inc	536951
RNAscope™ Positive Control Probe - Hs-PPIB - Homo sapiens peptidylprolyl isomerase B (cyclophilin B) (PPIB), mRNA	Advanced Cell Diagnostics, Inc	313901
RNAscope™ Negative Control Probe - DapB - Bacillus subtilis strain SMY methylglyoxal synthase (mgsA) gene, partial cds dihydrodipicolinate reductase (dapB) gene, complete cds and YpjD (ypjD) gene, partial cds	Advanced Cell Diagnostics, Inc	310043

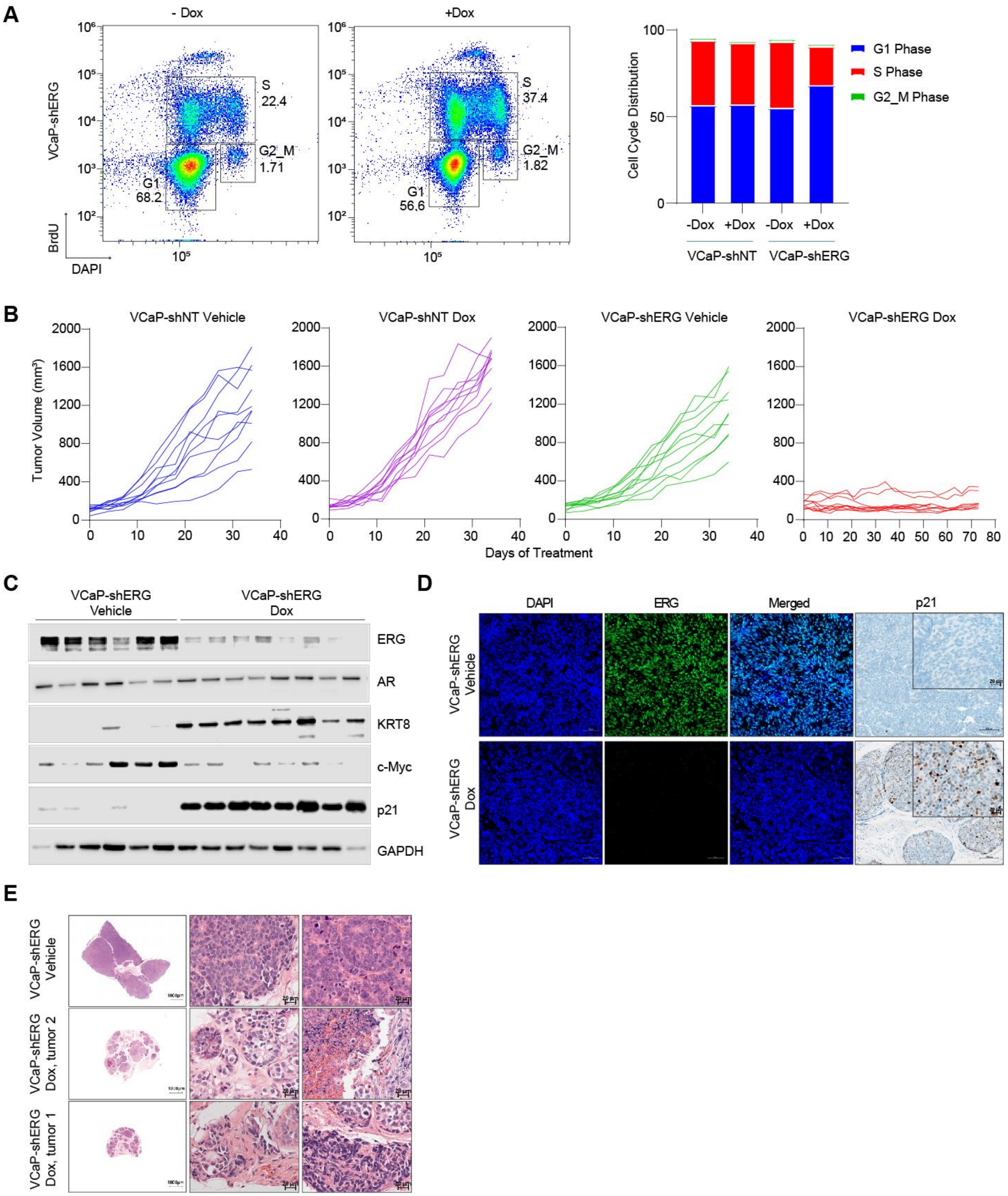
A
- Dox
+Dox
VCaP-shERG
BrdU
DAPI
S
22.4
G2_M
1.71
G1
68.2
S
37.4
G2_M
1.82
G1
56.6
Cell Cycle Distribution
G1 Phase
S Phase
G2_M Phase
-Dox +Dox -Dox +Dox
VCaP-shNT VCaP-shERG
B
VCaP-shNT Vehicle
VCaP-shNT Dox
VCaP-shERG Vehicle
VCaP-shERG Dox
Tumor Volume (mm³)
Days of Treatment
C
VCaP-shERG Vehicle
VCaP-shERG Dox
ERG
AR
KRT8
c-Myc
p21
GAPDH
D
DAPI
ERG
Merged
p21
VCaP-shERG Vehicle
VCaP-shERG Dox
E
VCaP-shERG Vehicle
VCaP-shERG Dox, tumor 2
VCaP-shERG Dox, tumor 1

F

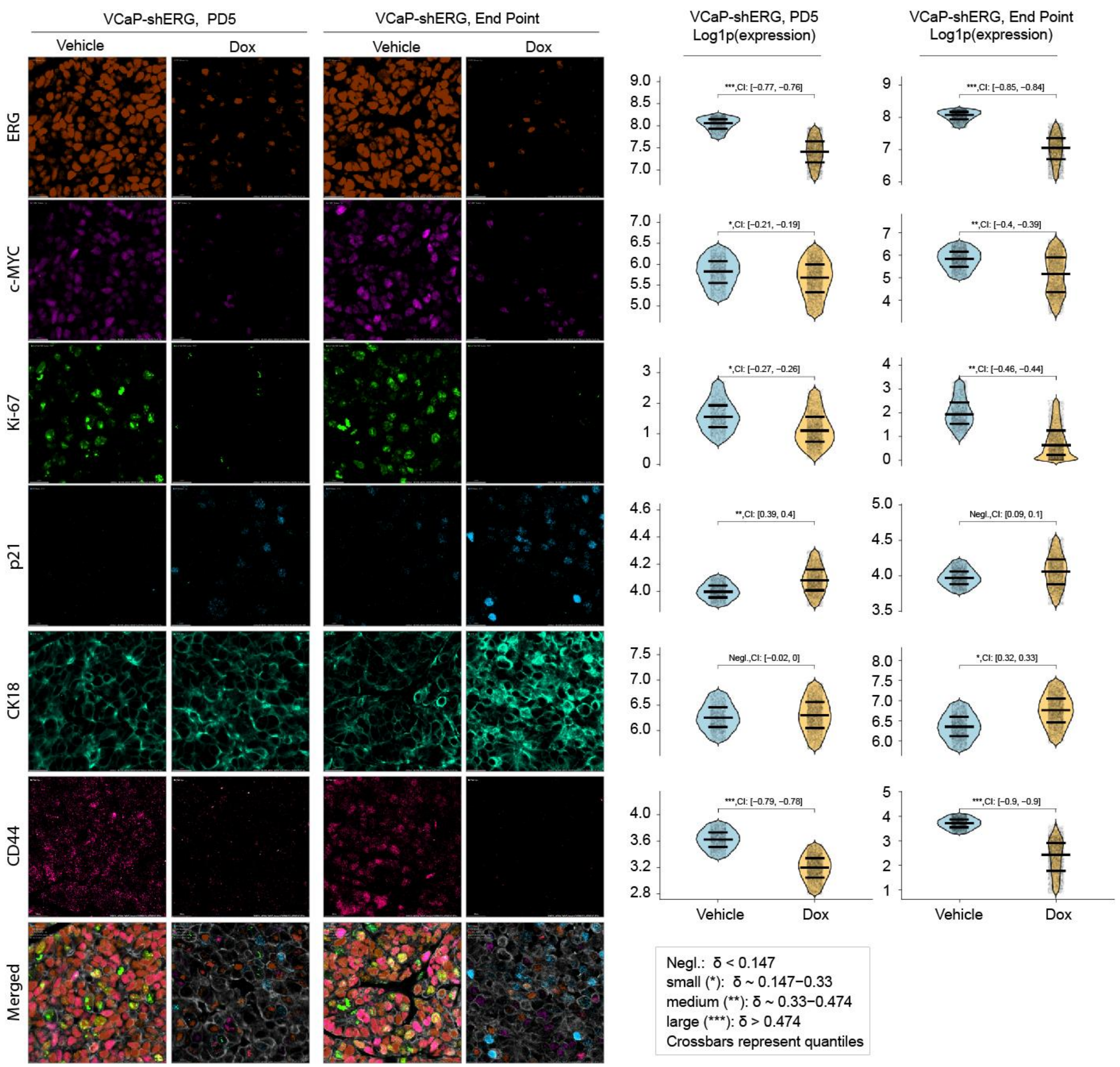

Fig. S1. (**A**) Doxycycline-induced ERG knockdown in the VCaP-shERG model for 48 hours reduces the S-phase population and induces G0/G1 cell-cycle arrest, as measured by flow cytometry. (**B**) Individual tumor volumes for VCaP-shRNA xenografts with or without doxycycline chow in SCID mice, measured twice weekly using calipers. (**C**) Immunoblot analysis of ERG and the indicated proteins in VCaP-shRNA xenograft tumors; GAPDH served as the loading control. (**D**) Representative images from the endpoint study, including immunofluorescence staining for ERG and immunohistochemistry for p21. (**E**) Representative H&E staining for vehicle- and doxycycline-treated tumors from VCaP-shRNA xenograft at the endpoint (partial data also shown in **Fig. 1K**). (**F**) Left: representative Seq-mIF images showing individual target staining from both PD5 and endpoint tumors in the VCaP-shERG xenograft study. Right: Violin plots showing log1p-transformed expression levels of individual targets across single cells based

on Seq-mIF staining (n > 30,000 cells), with Cliff's delta effect sizes and corresponding 95% confidence intervals reported for group comparisons.

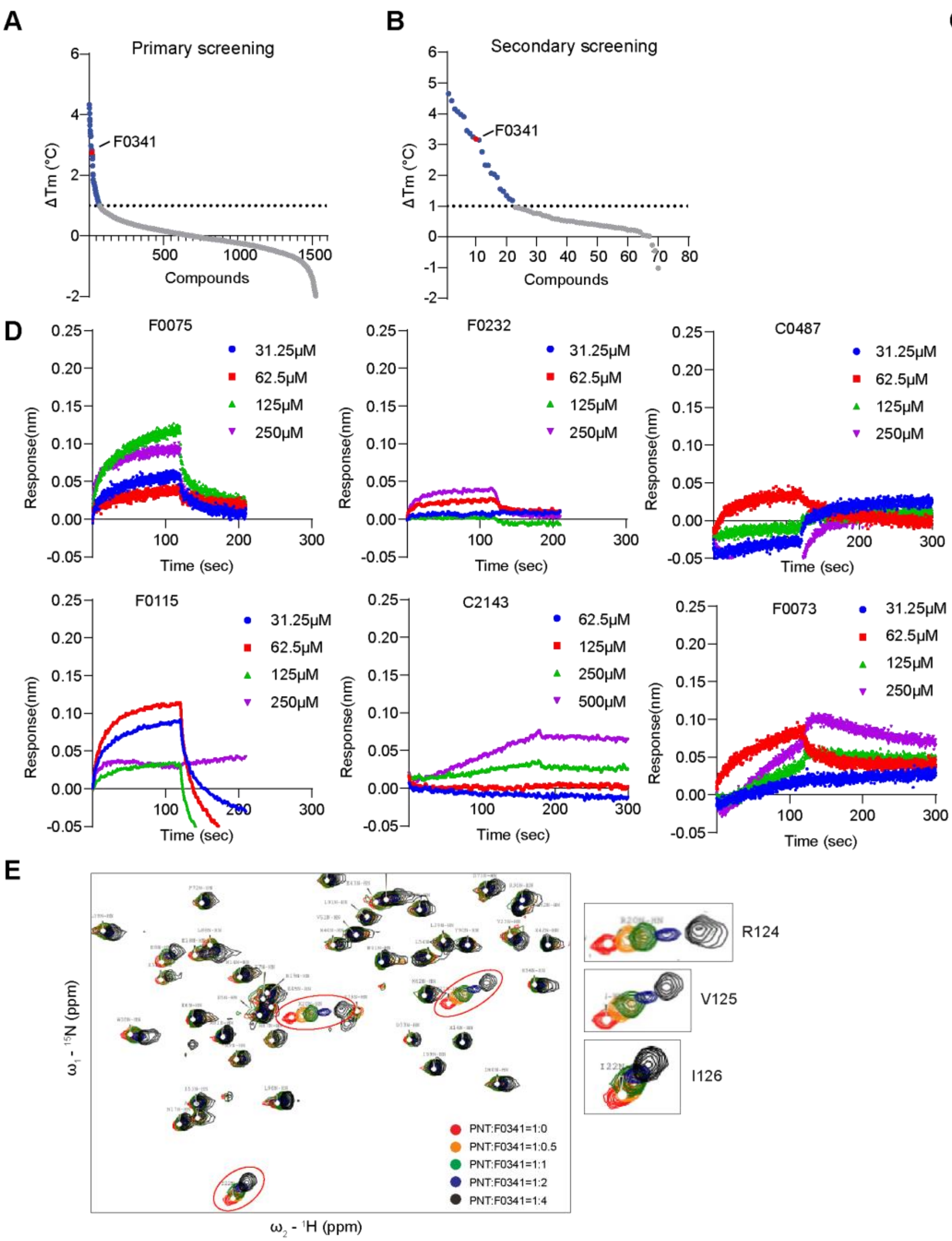

C

#	CCG Catalog	ΔTm(°C)	STD
1	F0115	4.65	0.18
2	P3051	4.42	0.74
3	F0923	4.15	0.27
4	F0271	4.07	0.33
5	P3191	3.9	0.08
6	V9561	3.45	0.73
7	F0075	3.36	0.29
8	F0044	3.24	0.43
9	F0341	3.18	0.24
10	C8712	3.15	0.87
11	P6261	2.76	0.4
12	C2143	2.33	0.27
13	F0027	2.06	0.53
14	Y6832	2.03	0.28
15	K1272	1.93	0.42
16	F0073	1.55	0.25
17	C0044	1.48	0.22
18	Y4486	1.34	0.2
19	P0063	1.23	0.38
20	F0232	1.18	0.14
21	C0437	1.08	0.45
22	C0487	1.06	0.18

Fig. S2. (**A**) Primary screening of the compound library (1,655 compounds total) by DSF at 200 μM. The top 72 hit compounds with a thermal shift (ΔTm) > 1 are highlighted in blue. (**B**) Secondary DSF screening of 72 hits at 200 μM (four technical replicates), confirming 22 compounds with ΔTm > 1 °C (blue). (**C**) Thermal stability profiles of the 22 candidate compounds. (**D**) Representative BLI sensorgrams showing binding of the candidate hit compounds to the PNT domain protein. (**E**) Binding site assessment by 2D NMR. Superimposed ^{1}H-^{15}N HSQC spectra of the PNT domain revealed the binding interface in a titration experiment. Three residues with the largest CSPs are highlighted with red circles, enlarged, and annotated with residue names and numbers. Different colors represent varying PNT:F0341 molar ratios.

A

Compounds	Domain	ΔTm (°C)	SD
F0341	PNT	5.25	0.18
Y0257	PNT	-1.51	0.04
Y0253	PNT	1.38	0.22
Y0226	PNT	-0.59	0.12
Y0222	PNT	0.39	0.09
Y0227	PNT	0.27	0.17
Y0223	PNT	0.43	0.19
Y0228	PNT	3.4	0.19
Y0318	PNT	8.51	0.1
Y0346	PNT	-0.15	0.14
Y0344	PNT	-1.11	0.15
Y0747	PNT	0.82	0.13
Y0739	PNT	-0.04	0.18
Y0636	PNT	5.54	0.24
Y0640	PNT	0.73	0.15
Y0743	PNT	-0.18	0.17
Y0752	PNT	-0.61	0.21

B

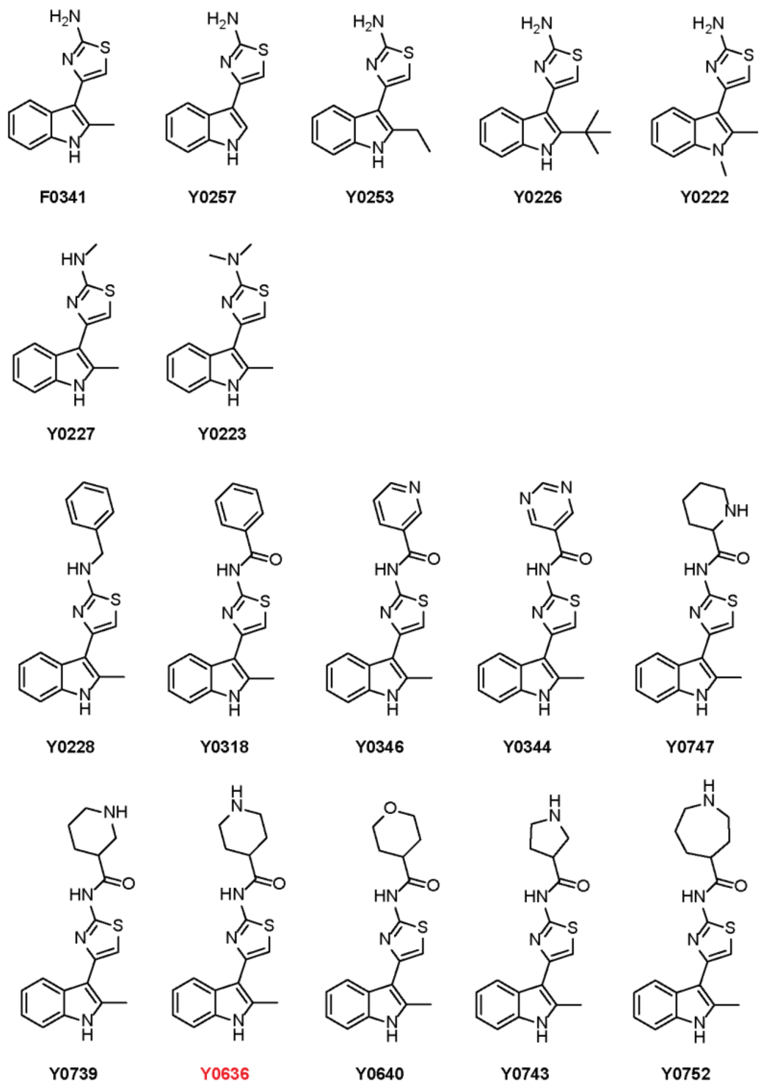

Fig. S3. (**A**) Thermal stability profiles of the new synthesized compounds at 100 μM for the ERG PNT domain protein by DSF assay. The ΔTm values are averages of seven technical replicates ± SD. (**B**) Chemical structures of the new synthesized compounds.

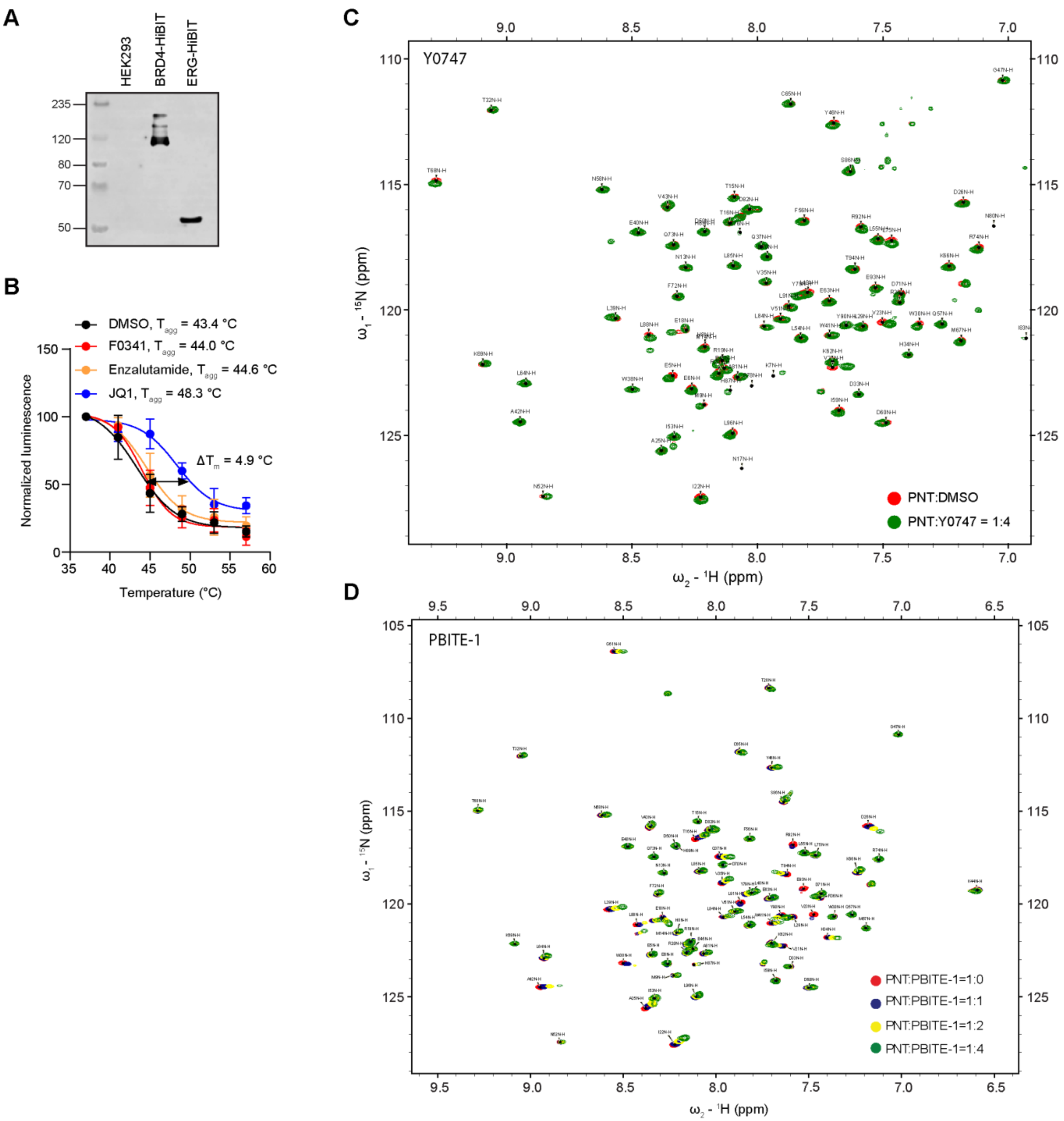

Fig. S4. (**A**) Immunoblots of ERG or BRD4 protein in HEK293-HiBiT cells. (**B**) HiBIT-CETSA assay for BRD4. HEK293 cells transfected with HiBIT-BRD4 plasmid for 24 hours were treated with DMSO, F0341, enzalutamide, or JQ1 (200 μM, 1 hour), followed by heating from 37-57 °C. Data represent mean ± SD from eight technical replicates. (**C-D**) NMR titration analysis of the negative control compound Y0747 (top) and PBITE-1 (bottom) with the PNT domain protein. Spectra at different PNT:compound molar ratios are shown in distinct colors.

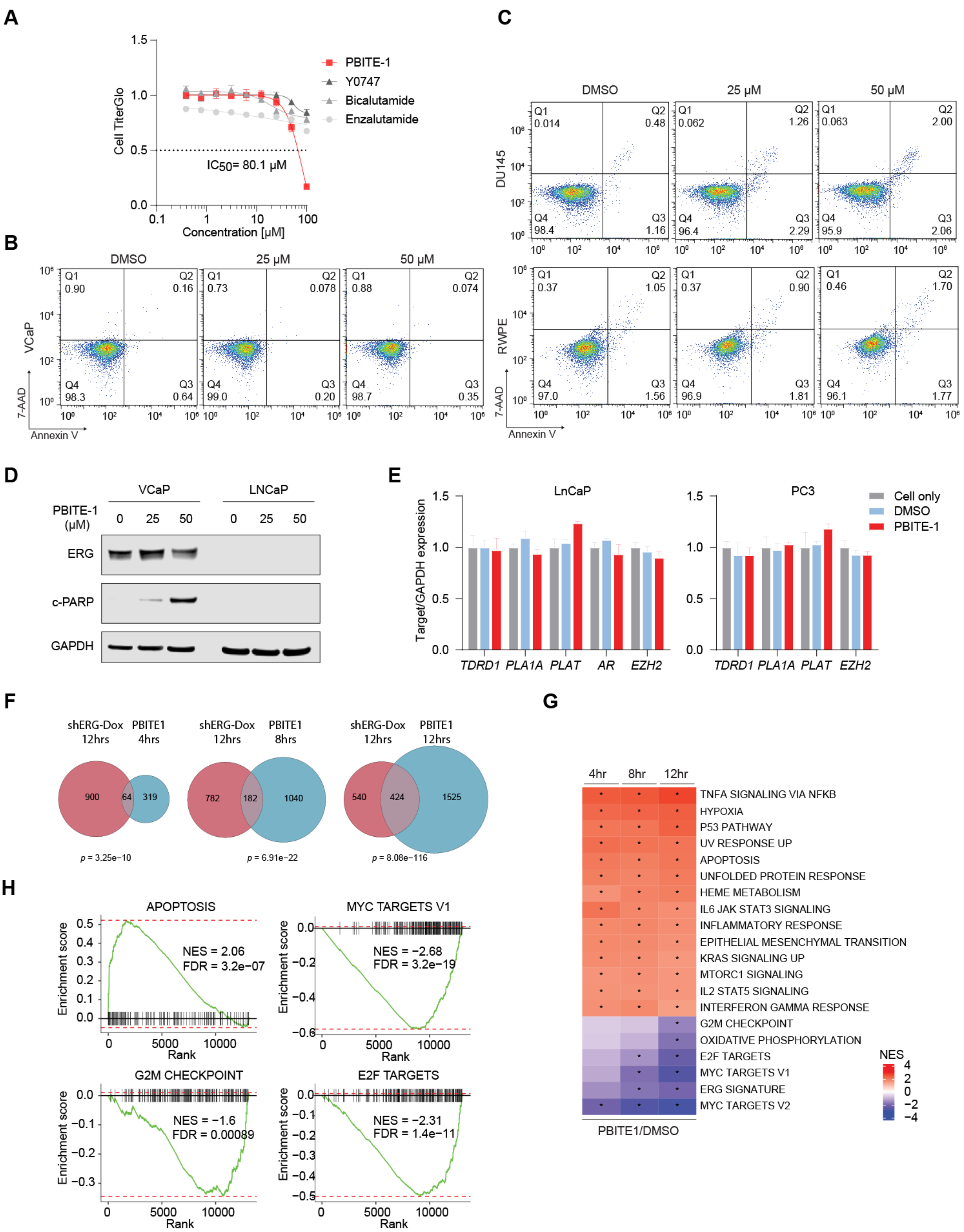
A
Cell TiterGlo
Concentration [μM]
IC50= 80.1 μM
PBITE-1
Y0747
Bicalutamide
Enzalutamide
B
DMSO
25 μM
50 μM
VCaP
7-AAD
Annexin V
C
DU145
RWPE
D
VCaP
LNCaP
PBITE-1 (μM)
0 25 50 0 25 50
ERG
c-PARP
GAPDH
E
LnCaP
PC3
Target/GAPDH expression
TDRD1 PLA1A PLAT AR EZH2
Cell only
DMSO
PBITE-1
F
shERG-Dox 12hrs
PBITE1 4hrs
PBITE1 8hrs
PBITE1 12hrs
p = 3.25e−10
p = 6.91e−22
p = 8.08e−116
G
4hr 8hr 12hr
TNFA SIGNALING VIA NFKB
HYPOXIA
P53 PATHWAY
UV RESPONSE UP
APOPTOSIS
UNFOLDED PROTEIN RESPONSE
HEME METABOLISM
IL6 JAK STAT3 SIGNALING
INFLAMMATORY RESPONSE
EPITHELIAL MESENCHYMAL TRANSITION
KRAS SIGNALING UP
MTORC1 SIGNALING
IL2 STAT5 SIGNALING
INTERFERON GAMMA RESPONSE
G2M CHECKPOINT
OXIDATIVE PHOSPHORYLATION
E2F TARGETS
MYC TARGETS V1
ERG SIGNATURE
MYC TARGETS V2
NES
PBITE1/DMSO
H
APOPTOSIS
NES = 2.06
FDR = 3.2e−07
MYC TARGETS V1
NES = −2.68
FDR = 3.2e−19
G2M CHECKPOINT
NES = −1.6
FDR = 0.00089
E2F TARGETS
NES = −2.31
FDR = 1.4e−11
Enrichment score
Rank

I

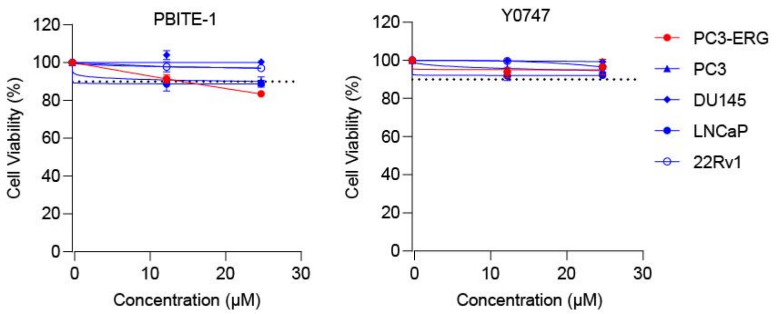

J

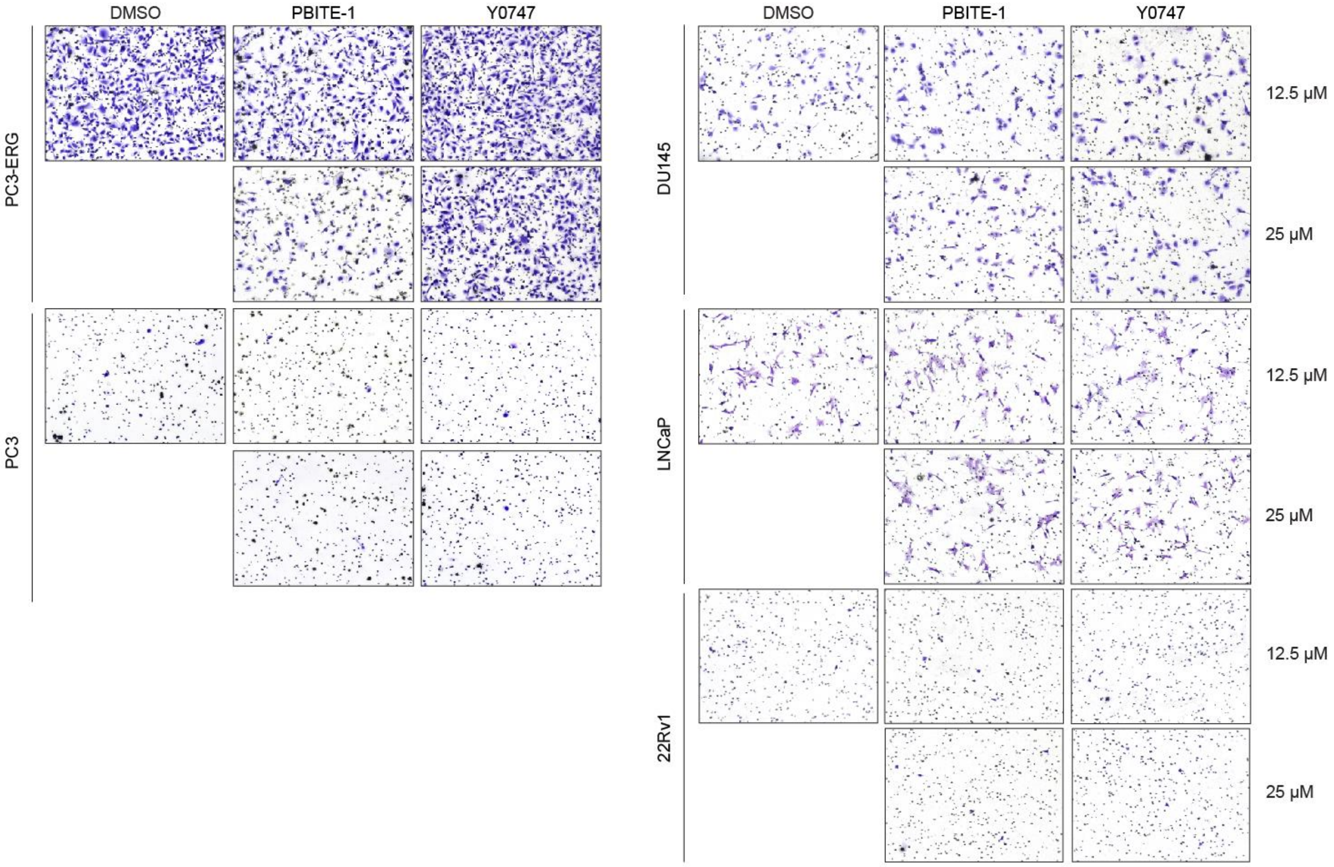

Fig. S5. (**A**) Cell viability of VCaP cells after 24-hour treatment with PBITE-1, Y0747, enzalutamide, or bicalutamide at the indicated concentrations. Data shown as mean ± SD from three independent experiments. (**B**) Apoptosis analysis in VCaP cells treated with Y0747 for 24 hours, measured by Annexin V/7-AAD staining. (**C**) Representative apoptosis analysis in DU145 and RWPE cells following 24-hour PBITE-1 treatment, assessed by flow cytometry using Annexin V/7-AAD staining. (**D**) Immunoblot analysis of ERG and the apoptosis marker c-PARP in VCaP and LNCaP cells treated with PBITE-1 at the indicated concentrations for 24 hours. (**E**) Relative expression of ERG target genes in LNCaP and PC3 cells (both are ERG-negative cell lines) after 8-hour treatment with PBITE-1, measured by quantitative PCR. Data represent mean ± SD of three technical replicates. (**F**) Venn diagram

illustrating the overlap of down-regulated genes ($log_2FC < -1$ and adjusted P value < 0.05) between shERG- and PBITE-1-treated VCaP cells. (**G**) Top HALLMARK pathway analyses of RNA-seq in VCaP cells upon PBITE-1 treatment (50 μM) at different time points. (**H**) GSEA net enrichment score (NES) plot of significantly altered hallmark pathways in VCaP cells treated with PBITE-1 for 12 hours. NES, normalized enrichment score; FDR, false discovery rate; n=3 biological replicates. Statistical significance was assessed using a two-sided GSEA permutation test with adjustment for multiple comparisons. (**I**) Cell viability measured by CellTiter-Glo assay in ERG-positive and ERG-negative cell lines treated with PBITE-1 or Y0747 at the indicated concentrations for 24 hours. Data represents mean ± SD from three independent experiments. (**J**) Representative images from Boyden chamber transwell invasion assays of PC3-ERG, PC3, DU145, and 22Rv1 cells treated with PBITE-1 or Y0747 for 24 hours in Matrigel-coated chambers, followed by fixation, crystal violet staining, and imaging. Error bars represent ± SEM. All statistical significance was determined by one-way ANOVA: *$p < 0.05$, **$p < 0.01$, ***$p < 0.001$, ****$p < 0.0001$.

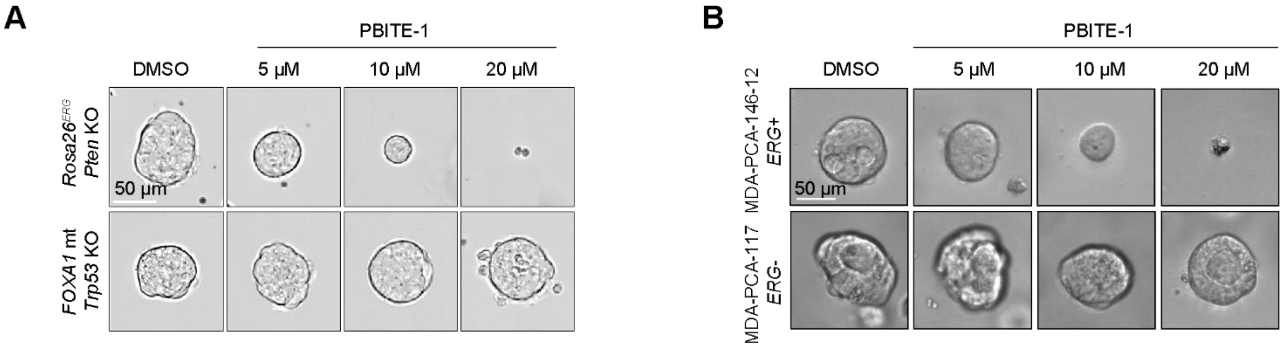

Fig. S6. (**A**) Representative images of mouse organoids after PBITE-1 treatment at the indicated doses. (**B**) Representative images of human organoids after PBITE-1 treatment at the indicated doses.

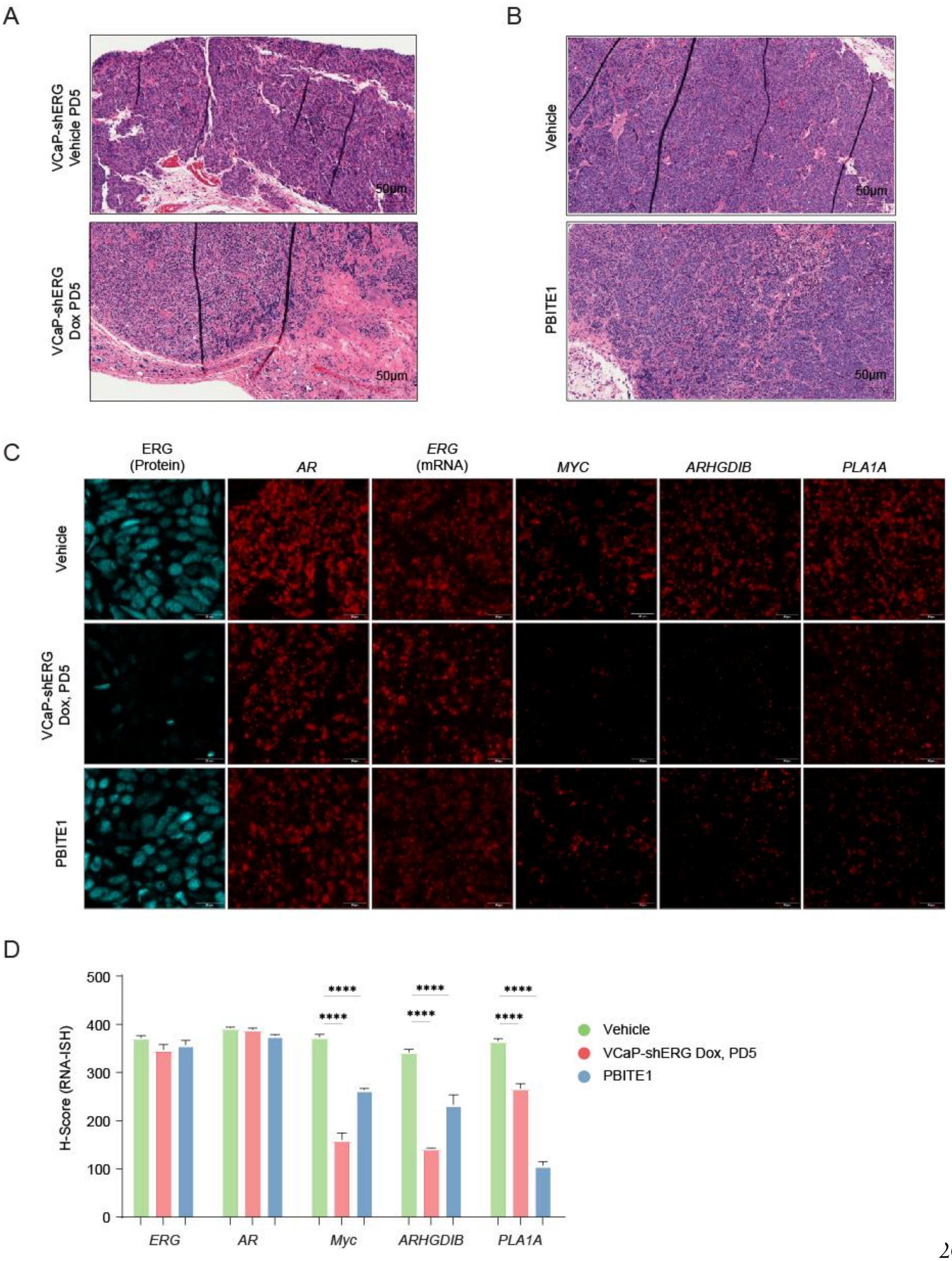

A
VCaP-shERG Vehicle PD5
VCaP-shERG Dox PD5
50µm
50µm
B
Vehicle
PBITE1
50µm
50µm
C
ERG (Protein)
AR
ERG (mRNA)
MYC
ARHGDIB
PLA1A
Vehicle
VCaP-shERG Dox, PD5
PBITE1
D
H-Score (RNA-ISH)
500
400
300
200
100
0
ERG
AR
Myc
ARHGDIB
PLA1A
****
****
****
****
****
****
Vehicle
VCaP-shERG Dox, PD5
PBITE1

Fig. S7. (**A**) Representative H&E staining of VCaP-shERG xenograft tumors following 5 days of treatment with doxycycline or vehicle control. (**B**) Representative H&E staining of VCaP xenograft tumors following 5 days of daily treatment with 100 μM PBITE-1 or vehicle control. (**C**) Representative combined immunofluorescence staining of ERG and RNA in situ hybridization (RNA-ISH) for the indicated ERG-regulated genes in tumor sections from VCaP-shERG and PBITE-1-treated VCaP xenograft models from the PD5 studies shown in (**A-B**). (**D**) Quantification of RNA-ISH–positive cells from (**C**) across treatment groups. Data are presented as mean ± SD. All statistical significance was assessed by one-way ANOVA: *$p < 0.05$, **$p < 0.01$, ***$p < 0.001$, ****$p < 0.0001$.

Chemistry

General information

All reagents and solvents for chemical synthesis were purchased commercially and not purified further, unless this has been stated. Reactions were monitored in the study by thin-layer chromatography (TLC) or Waters A21UPD315A Liquid Chromatograph-Mass Spectrometer (LC-MS). The spots on TLC were visualized by UV (254/365 nm). All indicated compounds were purified by column chromatography on silica gel (300-400 mesh), unless otherwise stated. The ^{1}H, ^{13}C, ^{19}F NMR spectra were detected on a Bruker AVANCE 400 spectrometer (Bruker Company, Germany), Agilent DD2 500 spectrometer (Agilent Technologies Inc., USA), or Bruker AVANCE 600 spectrometer (Bruker Company, Germany). The spectra of high-resolution mass (HRMS) were analyzed by a Bruker MaXis 4G TOF Mass Spectrometer and ESI source. Purities of all final compounds were identified by High Performance Liquid Chromatography (HPLC) using the Agilent 1200 system. HPLC condition: Triart C18 reversed-phase column, 5 μm, 4.6 mm × 250 mm, and a flow rate 1.0 mL/min, beginning with a 15 minute gradient from 0.1% TFA in water and acetonitrile 9:1 mixture to 0.1% TFA in acetonitrile; this ended with 0.1% TFA in acetonitrile for 5 minutes. All final compounds were confirmed to have a purity > 95% by HPLC analysis using the Agilent 1260 system.

Compounds synthesis and characterization

Procedure I:

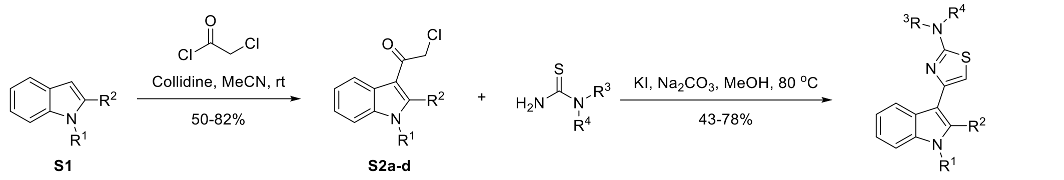

S1a: R^1 = H, R^2 = Me;
S1b: R^1 = Me, R^2 = Me;
S1c: R^1 = H, R^2 = Et;
S1d: R^1 = H, R^2 =*t*-Bu.

S2a: R^1 = H, R^2 = Me;
S2b: R^1 = Me, R^2 = Me;
S2c: R^1 = H, R^2 = Et;
S2d: R^1 = H, R^2 =*t*-Bu.

F0341: R^2 = Me, R^1, R^3, R^4 = H;
Y0222: R^1, R^2 = Me, R^2, R^3, R^4 = H;
Y0253: R^2 = Et, R^1, R^3, R^4 = H;
Y0226: R^2 = *t*-Bu, R^1, R^3, R^4 = H;
Y0227: R^2, R^3 = Me, R^1, R^4 = H;
Y0223: R^2, R^3, R^4 = Me, R^1 = H;
Y0228: R^2 = H, R^3 = Bn, R^1, R^4 = H;

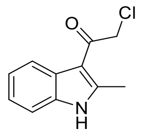

2-chloro-1-(2-methyl-1*H*-indol-3-yl)ethan-1-one (S2a)

The solution of **S1a** (5.0 g, 38.2 mmol) and collidine (9.2 g, 76.4 mmol) in MeCN (50 mL) was cooled to 0 ℃, then was added 2-chloroacetyl chloride (6.5 g, 57.3 mmol) in MeCN (10 mL). The mixture was warmed to room temperature and monitored by TLC. Once the reaction was completed, the mixture was cooled to 0 ℃, quenched with H_2O (50 mL) dropwise. Stir thoroughly and filter. Wash the filter cake with acetonitrile-H_2O mixture (200 mL, 1:1, *v*/*v*), then dry at 60 ℃ to obtain **S1a** (5.2 g, 25.2 mmol, 66% yield) as grayish-white solid. ^{1}H NMR (500 MHz, DMSO-d_6) δ 12.01 (s, 1H), 7.98 (dd, *J* = 5.9, 3.2 Hz, 1H), 7.37 (dd, *J* = 5.8, 3.3 Hz, 1H), 7.15 (dd, *J* = 6.0, 3.2 Hz, 2H), 4.91 (s, 2H), 2.69 (s, 3H).

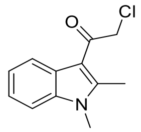

2-chloro-1-(1,2-dimethyl-1*H*-indol-3-yl)ethan-1-one (S2b)

Yellow solid (1.3 g, 5.7 mmol, 69% yield).^{1}H NMR (500 MHz, DMSO-d_6) δ 7.94 (dd, *J* = 6.6, 2.1 Hz, 1H), 7.54 (dd, *J* = 6.8, 2.0 Hz, 1H), 7.25–7.18 (m, 2H), 4.93 (s, 2H), 3.73 (s, 3H), 2.72 (s, 3H).

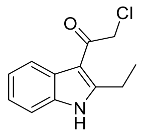

2-chloro-1-(2-ethyl-1*H*-indol-3-yl)ethan-1-one (S2c)

White solid (1.2 g, 5.1 mmol, 62% yield). ^{1}H NMR (500 MHz, Chloroform-*d*) δ 8.93 (s, 1H), 7.81 (d, *J* = 7.9 Hz, 1H), 7.41 (d, *J* = 7.9 Hz, 1H), 7.29 (t, *J* = 7.5 Hz, 1H), 7.27–7.23 (m, 1H), 4.76 (s, 2H), 3.23 (q, *J* = 7.5 Hz, 2H), 1.38 (t, *J* = 7.5 Hz, 3H).

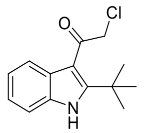

1-(2-(*tert*-butyl)-1*H*-indol-3-yl)-2-chloroethan-1-one (S2d)

White solid (1.5 g, 5.8 mmol, 84% yield).^{1}H NMR (500 MHz, Chloroform-*d*) δ 8.72 (s, br, 1H), 7.72 (d, *J* = 8.0 Hz, 1H), 7.43 (d, *J* = 8.0 Hz, 1H), 7.31–7.26 (m, 2H), 4.82 (s, 2H), 1.57 (s, 9H).

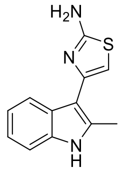

4-(2-methyl-1*H*-indol-3-yl)thiazol-2-amine (F0341)

To an oven-dried 250 mL round bottom flask equipped with a stirring bar was added **S2a** (1.4 g, 5.0 mmol), thiourea (570 mg, 7.5 mmol), KI (830 mg, 5.0 mmol), Na_2CO_3 (1.6 g, 15.0 mmol) in MeOH (0.5 M). The mixture was stirred for 12 h at 80 ℃. After TLC validating complete consumption of **S2a**, the mixture was filtrated and extracted by ethyl acetate. The organic phase was washed with brine, dried over anhydrous Na_2SO_4 and concentrated under reduced pressure. The crude was purified by column chromatography (EA: PE = 1:5 to 1:2) to give title compound **F0341** (800 mg, 3.5 mmol, 69% yield) as

purple-black solid. ^{1}H NMR (500 MHz, DMSO-d_6) δ 11.04 (s, 1H), 7.87 (d, J = 7.8 Hz, 1H), 7.25 (d, J = 7.8 Hz, 1H), 7.03–6.98 (t, J = 8.0 Hz, 1H), 6.96 (t, J = 8.0 Hz, 1H), 6.88 (s, 2H), 6.43 (s, 1H), 2.57 (s, 3H). HPLC purity: 98.16%, t_R = 8.518 min.

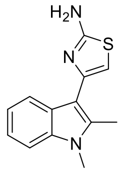

4-(1,2-dimethyl-1*H*-indol-3-yl)thiazol-2-amine (Y0222)

White solid (179 mg, 0.7 mmol, 72% yield). ^{1}H NMR (500 MHz, DMSO-d_6) δ 7.85 (d, J = 8.1 Hz, 1H), 7.38 (d, J = 8.1 Hz, 1H), 7.09 (t, J = 7.5 Hz, 1H), 7.01 (t, J = 7.5 Hz, 1H), 6.93 (s, 2H), 6.45 (s, 1H), 3.67 (s, 3H), 2.62 (s, 3H). ^{13}C NMR (126 MHz, DMSO-d_6) δ 167.68, 146.69, 136.61, 135.47, 126.35, 121.01, 120.01, 119.59, 109.57, 108.36, 100.01, 29.80, 12.01. HRMS (ESI) calcd for $C_{13}H_{14}N_3S$ $[M + H]^+$ 244.0903; found 244.0897. HPLC purity: 98.30%, t_R = 8.881 min.

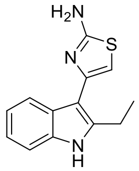

4-(2-ethyl-1*H*-indol-3-yl)thiazol-2-amine (Y0253)

Green solid (68.4 mg, 0.3 mmol, 28% yield). ^{1}H NMR (500 MHz, DMSO-d_6) δ 11.02 (s, 1H), 7.83 (d, J = 7.8 Hz, 1H), 7.27 (d, J = 7.9 Hz, 1H), 7.01 (t, J = 7.5 Hz, 1H), 6.96 (t, J = 7.9 Hz, 1H), 6.87 (s, 2H), 6.42 (s, 1H), 3.04 (q, J = 7.5 Hz, 2H), 1.25 (t, J = 7.6 Hz, 3H). ^{13}C NMR (126 MHz, DMSO-d_6) δ 167.56, 146.92, 139.86, 135.57, 127.13, 120.90, 120.14, 119.30, 111.04, 107.39, 99.42, 20.55, 14.54. HRMS (ESI) calcd for $C_{13}H_{14}N_3S$ $[M + H]^+$ 244.0903; found 244.0907. HPLC purity: 98.37%, t_R = 8.855 min.

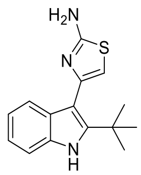

4-(2-(*tert*-butyl)-1*H*-indol-3-yl)thiazol-2-amine (Y0226)

White solid (176.2 mg, 0.7 mmol, 65% yield). ^{1}H NMR (400 MHz, DMSO-d_6) δ 10.71 (s, 1H), 7.32 (d, *J* = 7.9 Hz, 1H), 7.25 (d, *J* = 7.8 Hz, 1H), 7.01 (t, *J* = 7.3 Hz, 1H), 6.91 (t, *J* = 7.3 Hz, 1H), 6.82 (s, 2H), 6.35 (s, 1H), 1.37 (s, 9H). ^{13}C NMR (101 MHz, DMSO-d_6) δ 167.07, 146.68, 144.78, 134.49, 129.82, 120.83, 119.07, 118.78, 111.15, 107.15, 104.38, 33.56, 30.88. HRMS (ESI) calcd for $C_{15}H_{18}N_3S$ [M + H]$^+$ 272.26; found 272.1120. HPLC purity: 98.11%, t_R = 9.530 min.

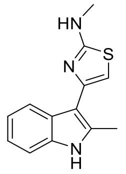

***N*-methyl-4-(2-methyl-1*H*-indol-3-yl)thiazol-2-amine (Y0227)**

Brownish-yellow solid (167.7 mg, 0.7 mmol, 69% yield). ^{1}H NMR (500 MHz, DMSO-d_6) δ 11.06 (s, 1H), 7.91 (d, *J* = 7.7 Hz, 1H), 7.42 (d, *J* = 4.5 Hz, 1H), 7.27 (d, *J* = 7.9 Hz, 1H), 7.02 (t, *J* = 7.1 Hz, 1H), 6.98 (t, *J* = 7.4 Hz, 1H), 6.50 (s, 1H), 2.88 (d, *J* = 4.7 Hz, 3H), 2.60 (s, 3H). ^{13}C NMR (126 MHz, DMSO-d_6) δ 168.79, 147.49, 135.42, 134.29, 127.18, 120.85, 120.14, 119.40, 110.90, 108.21, 98.36, 31.47, 14.00. HRMS (ESI) calcd for $C_{13}H_{14}N_3S$ [M + H]$^+$ 244.0903; found 244.0906. HPLC purity: 95.58%, t_R = 8.427 min.

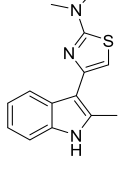

***N*,*N*-dimethyl-4-(2-methyl-1*H*-indol-3-yl)thiazol-2-amine (Y0223)**

Yellowish-white solid (161.9 mg, 0.6 mmol, 63% yield). ^{1}H NMR (500 MHz, Chloroform-*d*) δ 7.99–7.95 (m, 1H), 7.92 (s, 1H), 7.29–7.26 (m, 1H), 7.16–7.10 (m, 2H), 6.49 (s, 1H), 3.17 (s, 7H), 2.68 (s, 3H). ^{13}C NMR (126 MHz, Chloroform-*d*) δ 170.22, 147.77, 135.09, 133.39, 127.53, 121.30, 119.96, 119.93, 110.05, 109.22, 100.30, 40.25, 13.60. HRMS (ESI) calcd for $C_{14}H_{16}N_3S$ $[M + H]^+$ 258.1059; found 258.1056. HPLC purity: 100%, t_R = 8.676 min.

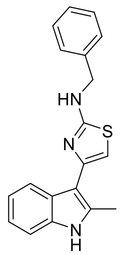

***N*-benzyl-4-(2-methyl-1*H*-indol-3-yl)thiazol-2-amine (Y0228)**

Dark red solid (111.6 mg, 0.4 mmol, 88% yield). ^{1}H NMR (500 MHz, DMSO-d_6) δ 11.05 (s, 1H), 8.08 (d, *J* = 6.0 Hz, 1H), 7.83 (d, *J* = 7.8 Hz, 1H), 7.39 (d, *J* = 7.4 Hz, 2H), 7.33 (t, *J* = 7.6 Hz, 2H), 7.25 (t, *J* = 7.4 Hz, 2H), 7.00 (t, *J* = 7.1 Hz, 1H), 6.95 (t, *J* = 7.3 Hz, 1H), 6.49 (s, 1H), 4.51 (d, *J* = 5.8 Hz, 2H), 2.55 (s, 3H). ^{13}C NMR (126 MHz, DMSO-d_6) δ 167.62, 147.17, 140.17, 135.39, 134.30, 128.71, 127.89, 127.28, 127.12, 120.84, 120.14, 119.40, 108.12, 98.63, 98.62, 48.21, 13.98. HRMS (ESI) calcd for $C_{19}H_{18}N_3S$ $[M + H]^+$ 320.1216; found 320.1219. HPLC purity: 95.39%, t_R = 10.761 min.

Procedure II:

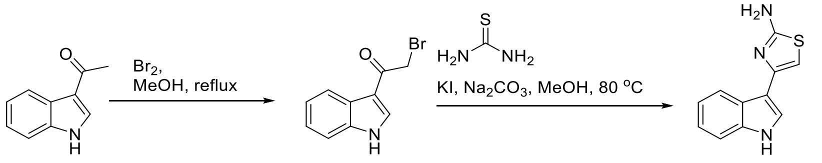

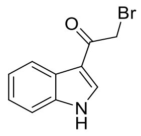

2-bromo-1-(1*H*-indol-3-yl)ethan-1-one (S2e)

To the solution of **S1c** (1.6 g, 10.0 mmol) in MeOH (50 mL) was added liquid bromine (2.7 g, 1.3 mmol). The mixture was refluxed and stirred for 5 h. After TLC validating complete consumption of **S1c**, the mixture was cooled to room temperature, quenched by saturated aq. $NaHCO_3$, and diluted with ethyl acetate. The organic phase was washed with brine, dried over anhydrous Na_2SO_4 and concentrated under reduced pressure. The crude was purified by column chromatography (EA: PE = 1:50 to 1:2) to give title compound **S2e** (405 mg, 1.7 mmol, 17%) as brown solid. ^{1}H NMR (500 MHz, DMSO-d_6) δ 12.14 (s, 1H), 8.47 (d, *J* = 3.2 Hz, 1H), 8.14 (d, *J* = 7.0 Hz, 1H), 7.48 (d, *J* = 8.1 Hz, 1H), 7.26–7.17 (m, 2H), 4.64 (s, 2H).

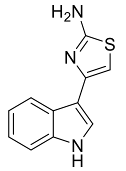

4-(1*H*-indol-3-yl)thiazol-2-amine (Y0257)

Dark red solid (60 mg, 0.3 mmol, 28% yield). ^{1}H NMR (500 MHz, Acetonitrile-d_3) δ 9.49 (s, 1H), 8.00 (d, *J* = 7.9 Hz, 1H), 7.65 (d, *J* = 2.6 Hz, 1H), 7.47 (dd, *J* = 7.7, 1.2 Hz, 1H), 7.24–7.15 (m, 2H), 6.68 (s, 1H), 5.85 (s, 2H). ^{13}C NMR (126 MHz, Acetonitrile-d_3) δ 167.55, 146.55, 136.79, 124.84, 124.23, 121.94, 119.97, 117.44, 112.15, 111.72, 98.92. HRMS (ESI) calcd for $C_{11}H_9N_3S$ $[M + H]^+$ 216.0590; found 216.0593. HPLC purity: 97.12%, t_R = 8.108 min.

Procedure III:

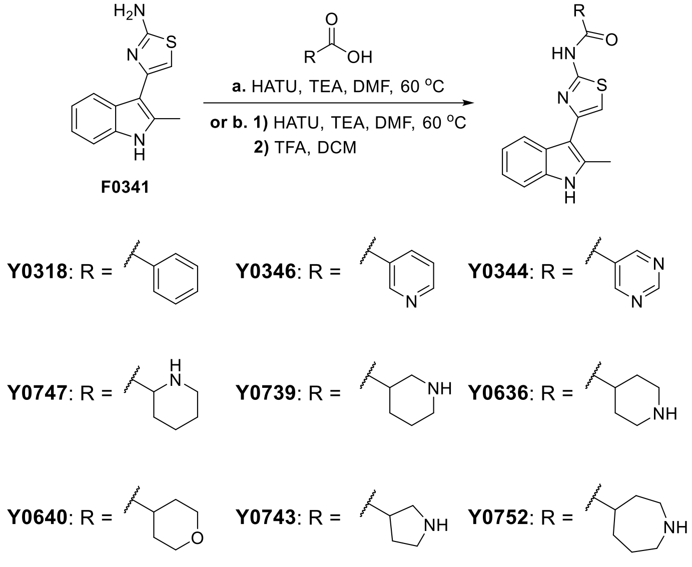

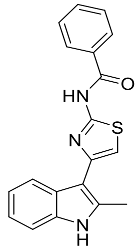

***N*-(4-(2-methyl-1*H*-indol-3-yl)thiazol-2-yl)benzamide (Y0318)**

To the solution of **F0341** (100 mg, 0.4 mmol), benzoic acid (88 mg, 0.7 mmol) and HATU in DMF (5 mL) was added TEA (111 mg, 1.1 mmol). The mixture was stirred for 2 h at 60 ℃. After TLC validating complete consumption of **F0341**, the mixture was cooled to room temperature and diluted with ethyl acetate. The organic phase was washed with aq. NaOH (1 M), dried over anhydrous Na_2SO_4 and concentrated under reduced pressure. The crude was purified by column chromatography (EA: PE = 1:50 to 1:2) to give title compound **Y0318** (32 mg, 0.1 mmol, 22%) as yellow solid. ^{1}H NMR (500 MHz, DMSO-d_6) δ 12.56 (s, 1H), 11.19 (s, 1H), 8.13 (d, *J* = 7.3 Hz, 2H), 8.02 (d, *J* = 7.6 Hz, 1H), 7.63 (t, *J* = 7.3 Hz, 1H), 7.55 (t, *J* = 7.6 Hz, 2H), 7.31 (d, *J* = 7.6 Hz, 1H), 7.12 (s, 1H), 7.06 (t, *J* = 7.5 Hz, 1H), 7.02 (t,

J = 7.2 Hz, 1H), 2.64 (s, 3H). ^{13}C NMR (126 MHz, DMSO-d_6) δ 165.56, 157.67, 146.37, 135.47, 134.54, 132.93, 132.75, 128.98, 128.68, 127.19, 121.11, 120.16, 119.54, 111.02, 107.59, 106.41, 13.82. HRMS (ESI) calcd for $C_{19}H_{16}N_3OS$ [M + H]$^+$ 334.1009; found 334.1013. HPLC purity: 99.13%, t_R = 14.455 min.

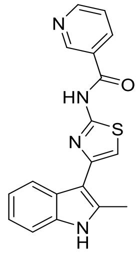

_N_-(4-(2-methyl-1*H*-indol-3-yl)thiazol-2-yl)nicotinamide (Y0346)

White solid (93.5 mg, 0.3 mmol, 63% yield). ^{1}H NMR (600 MHz, DMSO-d_6) δ 12.79 (s, 1H), 11.21 (s, 1H), 9.25 (s, 1H), 8.81 (dd, *J* = 4.8, 1.6 Hz, 1H), 8.46 (dt, *J* = 8.0, 1.8 Hz, 1H), 8.02 (d, *J* = 7.4 Hz, 1H), 7.62–7.58 (m, 1H), 7.32 (d, *J* = 7.8 Hz, 1H), 7.18 (s, 1H), 7.09–7.06 (m, 1H), 7.04 (td, *J* = 7.6, 1.1 Hz, 1H), 2.66 (s, 3H). ^{13}C NMR (151 MHz, DMSO-d_6) δ 164.30, 157.32, 153.26, 149.67, 146.46, 136.41, 135.49, 134.62, 128.75, 127.19, 124.00, 121.15, 120.09, 119.58, 111.07, 107.51, 106.66, 13.82. HRMS (ESI) calcd for $C_{18}H_{14}N4OS$ [M + H]$^+$ 335.0961; found 335.0964. HPLC purity: 97.63%, t_R = 10.220 min.

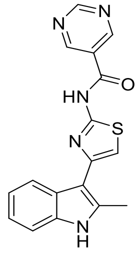

_N_-(4-(2-methyl-1*H*-indol-3-yl)thiazol-2-yl)pyrimidine-5-carboxamide (Y0344)

Orange-red solid (67 mg, 0.2 mmol, 45% yield). ^{1}H NMR (600 MHz, DMSO-d_6) δ 12.97 (s, 1H), 11.23 (s, 1H), 9.40 (s, 3H), 8.05–7.97 (m, 1H), 7.33 (d, *J* = 7.8 Hz, 1H), 7.20 (s, 1H), 7.08 (t, *J* = 7.2 Hz, 1H), 7.04 (t, *J* = 7.2 Hz, 1H), 2.66 (s, 3H). ^{13}C NMR (151 MHz, DMSO-d_6) δ 162.60, 161.02, 157.18,

157.06, 146.55, 135.50, 134.68, 127.16, 121.19, 120.02, 119.62, 111.10, 107.44, 106.85, 13.81. HRMS (ESI) calcd for $C_{17}H_{14}N_5OS$ $[M + H]^+$ 336.0922; found 336.0914. HPLC purity: 99.11%, t_R = 11.596 min.

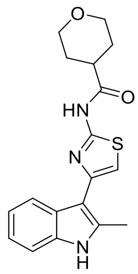

***N*-(4-(2-methyl-1*H*-indol-3-yl)thiazol-2-yl)tetrahydro-2*H*-pyran-4-carboxamide (Y0640)**

White solid (114 mg, 0.3 mmol, 76% yield). ^1H NMR (600 MHz, DMSO-d_6) δ 12.09 (s, 1H), 11.18 (s, 1H), 7.96 (d, *J* = 7.0 Hz, 1H), 7.31 (d, *J* = 7.2 Hz, 1H), 7.03 (m, 3H), 3.91 (s, 2H), 3.37 (m, 2H), 2.62 (m, 3H), 1.73 (m, 4H). ^{13}C NMR (151 MHz, DMSO-d_6) δ 173.41, 157.20, 146.08, 135.47, 134.46, 127.15, 121.09, 120.03, 119.53, 111.05, 107.57, 105.81, 66.75, 40.88, 29.04, 13.83. HRMS (ESI) calcd for $C_{18}H_{19}N_3O_2S$ $[M + H]^+$ 342.1271; found 342.1270. HPLC purity: 99.4%, t_R = 11.956 min.

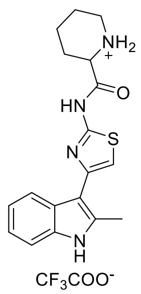

***N*-(4-(2-methyl-1*H*-indol-3-yl)thiazol-2-yl)piperidine-2-carboxamide (Y0747)**

To the solution of **F0341** (100 mg, 0.4 mmol), 1-(*tert*-butoxycarbonyl)piperidine-2-carboxylic acid (88 mg, 0.7 mmol) and HATU in DMF (5 mL) was added TEA (111 mg, 1.1 mmol). The mixture was stirred for 2 h at 60 ℃. After TLC validating complete consumption of **F0341**, the mixture was cooled to room temperature and diluted with ethyl acetate. The organic phase was washed with aq. NaOH (1 M), dried

over anhydrous Na_2SO_4 and concentrated under reduced pressure. The crude product was used in the next step without further purification.

To the solution of the crude intermediate in DCM (4.5 mL) was added TFA (0.5 mL). The mixture was stirred for 2 h at room temperature. After LCMS validating complete consumption of the intermediate, the mixture was concentrated under the reduced pressure to afford the residue. The residue was washed by aether to afford the final product as white salt (76 mg, 0.2 mmol, 50% yield). ^{1}H NMR (600 MHz, DMSO-d_6) δ 12.70 (s, 1H), 11.23 (s, 1H), 8.98 (s, 2H), 7.95 (d, *J* = 7.7 Hz, 1H), 7.33 (d, *J* = 7.9 Hz, 1H), 7.19 (s, 1H), 7.08 (t, *J* = 7.4 Hz, 1H), 7.03 (t, *J* = 7.9 Hz, 1H), 4.07 (dd, *J* = 12.1, 2.9 Hz, 1H), 3.35 (s, 1H), 3.03 (td, *J* = 12.6, 3.1 Hz, 1H), 2.63 (s, 3H), 2.30 (d, *J* = 13.5 Hz, 1H), 1.86 (d, *J* = 12.9 Hz, 1H), 1.76 (d, *J* = 13.7 Hz, 1H), 1.66 (qd, *J* = 12.8, 6.5 Hz, 2H), 1.55 (ddd, *J* = 15.8, 8.1, 3.2 Hz, 1H). ^{13}C NMR (151 MHz, DMSO-d_6) δ 166.81, 155.06, 145.44, 134.40, 133.65, 125.96, 120.13, 118.78, 118.57, 110.08, 106.12, 105.56, 56.69, 42.87, 26.24, 21.02, 20.62, 12.73. ^{19}F NMR (376 MHz, DMSO-d_6) δ -69.16, -71.05. HRMS (ESI) calcd for $C_{18}H_{20}N_4OS$ $[M + H]^+$ 341.1431; found 341.1433. HPLC purity: 99.50%, t_R = 9.331 min.

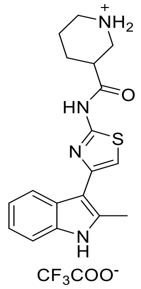

***N*-(4-(2-methyl-1*H*-indol-3-yl)thiazol-2-yl)piperidine-3-carboxamide (Y0739)**

Brown salt (67 mg, 0.2 mmol, 45% yield). ^{1}H NMR (600 MHz, DMSO-d_6) δ 12.37 (s, 1H), 11.24 (s, 1H), 8.83 (s, 2H), 7.96 (d, J = 7.8 Hz, 1H), 7.32 (d, J = 7.9 Hz, 1H), 7.10 (s, 1H), 7.06 (t, J = 7.5 Hz, 1H), 7.02 (t, J = 7.2 Hz, 1H), 3.41 (d, J = 14.5 Hz, 1H), 3.21 (d, J = 12.4 Hz, 1H), 3.13 (t, J = 11.5 Hz, 1H), 3.09–3.01 (m, 1H), 2.94 (t, J = 10.2 Hz, 1H), 2.62 (s, 3H), 2.12–2.06 (m, 1H), 1.88–1.81 (m, 1H), 1.70 (p, J = 9.8, 8.3 Hz, 2H). ^{13}C NMR (151 MHz, DMSO) δ 171.26, 158.94, 158.74, 158.53, 158.33, 156.78, 146.25, 135.49, 134.58, 127.12, 121.12, 120.75, 119.98, 119.56, 118.76, 116.77, 114.78, 111.11, 107.43, 106.05, 44.19, 43.44, 40.40, 39.09, 26.45, 21.59, 13.82. ^{19}F NMR (376 MHz, DMSO) δ -74.78, -74.79. HRMS (ESI) calcd for $C_{18}H_{20}N_4OS$ $[M + H]^+$ 341.1431; found 341.1433. HPLC purity: 99.51%, t_R = 9.040 min.

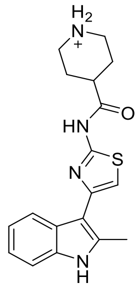

***N*-(4-(2-methyl-1*H*-indol-3-yl)thiazol-2-yl)piperidine-4-carboxamide (Y0636, PBITE-1)**

White-yellow salt (53 mg, 0.2 mmol, 35% yield). ^{1}H NMR (600 MHz, DMSO-d_6) δ 11.22 (s, 1H), 7.96 (d, J = 7.8 Hz, 1H), 7.32 (d, J = 7.9 Hz, 1H), 7.09–6.99 (m, 3H), 3.04 (d, J = 11.8 Hz, 2H), 2.67 (td, J = 11.7, 10.0, 6.0 Hz, 1H), 2.62 (s, 3H), 2.56 (dd, J = 16.4, 7.2 Hz, 4H), 1.78 (d, J = 13.7 Hz, 2H), 1.58 (q, J = 12.2 Hz, 2H). ^{13}C NMR (151 MHz, DMSO-d_6) δ 173.96, 158.88, 158.67, 158.47, 158.26, 157.22, 146.08, 135.47, 134.49, 127.14, 121.07, 120.75, 120.01, 119.53, 118.76, 116.77, 114.78, 111.07,

107.54, 105.70, 45.40, 42.15, 28.95, 13.81. ^{19}F NMR (376 MHz, DMSO-d_6) δ -73.42. HRMS (ESI) calcd for $C_{18}H_{20}N_4OS$ [M + H]$^+$ 341.1431; found 341.1434. HPLC purity: 96.40%, t_R = 8.892 min.

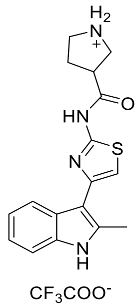

N-(4-(2-methyl-1*H*-indol-3-yl)thiazol-2-yl)pyrrolidine-3-carboxamide (Y0743)

White salt (48 mg, 0.1 mmol, 33% yield). ^{1}H NMR (600 MHz, DMSO-d_6) δ 12.41 (s, 1H), 11.23 (s, 1H), 9.08 (s, 2H), 7.96 (d, *J* = 7.8 Hz, 1H), 7.32 (d, *J* = 7.9 Hz, 1H), 7.11 (s, 1H), 7.06 (t, *J* = 7.5 Hz, 1H), 7.02 (t, *J* = 7.4 Hz, 1H), 3.46 (td, *J* = 12.0, 11.1, 3.6 Hz, 3H), 3.27 (t, *J* = 7.2 Hz, 2H), 2.62 (s, 3H), 2.31 (dq, *J* = 14.5, 7.2 Hz, 1H), 2.13 (dq, *J* = 13.4, 7.3 Hz, 1H). ^{13}C NMR (151 MHz, DMSO-d_6) δ 170.93, 158.91, 158.71, 158.50, 158.30, 156.91, 146.26, 135.48, 134.57, 127.11, 121.13, 119.94, 119.57, 111.11, 107.43, 106.15, 47.24, 45.46, 42.46, 29.11, 13.82. ^{19}F NMR (376 MHz, DMSO) δ -74.36, -74.45. HRMS (ESI) calcd for $C_{17}H_{18}N_4OS$ [M + H]$^+$ 327.1274; found 327.1276. HPLC purity: 99.82%, t_R = 8.577 min.

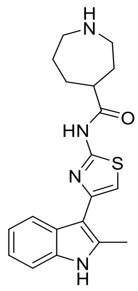

N-(4-(2-methyl-1*H*-indol-3-yl)thiazol-2-yl)azepane-4-carboxamide (Y0752)

Brown solid (purified by HPLC, 63 mg, 0.2 mmol, 41% yield). ^{1}H NMR (600 MHz, DMSO-d_6) δ 11.28 (s, 1H), 8.50 (s, 1H), 7.96 (d, J = 7.8 Hz, 1H), 7.32 (d, J = 7.9 Hz, 1H), 7.08–7.00 (m, 3H), 3.19–3.13 (m, 1H), 3.07–3.00 (m, 1H), 3.01–2.92 (m, 2H), 2.87 (tt, J = 8.9, 4.0 Hz, 1H), 2.62 (s, 3H), 1.96 (dddt, J = 35.6, 25.4, 14.6, 5.6 Hz, 4H), 1.82–1.66 (m, 2H). ^{13}C NMR (151 MHz, DMSO-d_6) δ 174.61, 166.75, 157.24, 146.13, 135.50, 134.51, 127.15, 121.07, 120.02, 119.53, 111.08, 107.55, 105.74, 46.59, 44.48, 43.99, 29.95, 29.64, 25.22, 13.84. HRMS (ESI) calcd for $C_{19}H_{22}N_4OS$ [M + H]$^+$ 355.1587; found 355.1587. HPLC purity: 100%, t_R = 9.291 min.

Procedure IV:

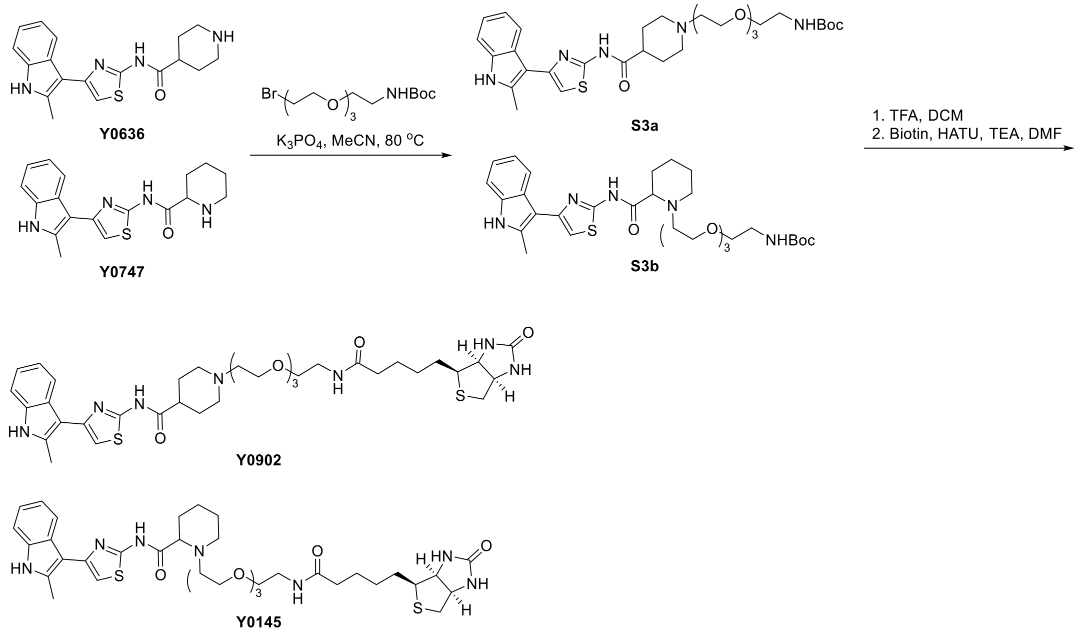

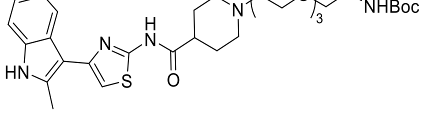

***tert*-butyl(2-(2-(2-(2-(4-((4-(2-methyl-1*H*-indol-3-yl)thiazol-2-yl)carbamoyl)piperidin-1-yl)ethoxy)ethoxy)ethoxy)ethyl)carbamate (S3a)**

To the solution of **PBITE-1** (170 mg, 0.5 mmol), tert-butyl (2-(2-(2-(2-bromoethoxy)ethoxy)ethoxy)ethyl)carbamate (237 mg, 0.7 mmol) in MeCN (10 mL) was added K_3PO_4 (212 mg, 2.0 mmol). The mixture was stirred for 2 h at 80 ℃. After TLC validating complete consumption of **PBITE-1**, the mixture was cooled to room temperature and diluted with ethyl acetate. The organic phase was washed with brine, dried over anhydrous Na_2SO_4 and concentrated under reduced pressure. The crude was purified by column chromatography (MeOH: DCM = 1:50 to 1:8) to give title compound **S3** (119 mg, 0.2 mmol, 38%) as yellow solid. ^{1}H NMR (500 MHz, Chloroform-*d*) δ 12.17 (s, 1H), 9.02 (s, 1H), 7.91 (d, *J* = 7.9 Hz, 1H), 7.31 (d, *J* = 8.0 Hz, 1H), 7.12 (t, *J* = 7.5 Hz, 1H), 7.06 (t, *J* = 7.5 Hz, 1H), 6.90 (s, 1H), 5.20 (s, 1H), 3.69–3.60 (m, 6H), 3.59 (d, *J* = 2.5 Hz, 2H), 3.54 (s, 2H), 3.48 (t, *J* = 5.6 Hz, 2H), 3.36–3.26 (m, 2H), 2.65 (s, 3H), 2.60 (d, *J* = 11.2 Hz, 2H), 2.33 (s, 2H), 2.27 (s, 3H), 1.59–1.49 (m, 3H), 1.43 (s, 9H).

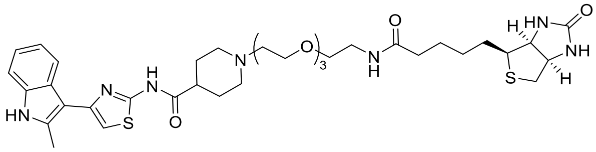

***N*-(4-(2-methyl-1*H*-indol-3-yl)thiazol-2-yl)-1-(13-oxo-17-((3*aS*,4*S*,6*aR*)-2-oxohexahydro-1*H*-thieno[3,4-d]imidazol-4-yl)-3,6,9-trioxa-12-azaheptadecyl)piperidine-4-carboxamide (Y0902, Bio-PBITE-1)**

To the solution of **S3** (102 mg, 0.2 mmol), tert-butyl (2-(2-(2-(2-bromoethoxy)ethoxy)ethoxy)ethyl)carbamate (237 mg, 0.7 mmol) in DCM (4.5 mL) was added TFA (0.5 mL). The mixture was stirred for 2 h at room temperature. After LCMS validating complete consumption of **S3**, the mixture was concentrated under the reduced pressure to afford the

residue. The crude product was used in the next step without further purification.

To the solution of the crude intermediate in DMF (5 mL) was added biotin (62 mg, 0.2 mmol), HATU (91 mg, 0.2 mmol) and TEA (80.8 mg, 0.8 mmol). The mixture was stirred for 2 h at 60 ℃. After LCMS validating complete consumption of the intermediate, the mixture was purified by HPLC (MeCN: H_2O = 0 to 100%) to afford the final product as white solid (43 mg, 0.1 mmol, 36% yield). ^{1}H NMR (600 MHz, DMSO-d_6) δ 12.05 (s, 1H), 11.17 (s, 1H), 7.96 (d, *J* = 7.9 Hz, 1H), 7.83 (t, *J* = 5.6 Hz, 1H), 7.31 (d, *J* = 7.9 Hz, 1H), 7.07–6.99 (m, 3H), 6.42 (s, 1H), 6.35 (s, 1H), 4.31–4.26 (m, 1H), 4.11 (ddt, *J* = 7.4, 4.8, 2.4 Hz, 1H), 3.56–3.49 (m, 10H), 3.40 (t, *J* = 5.9 Hz, 2H), 3.19 (dt, *J* = 11.6, 5.4 Hz, 2H), 3.08 (ddd, *J* = 8.6, 6.1, 4.6 Hz, 1H), 2.95 (d, *J* = 10.7 Hz, 2H), 2.80 (dd, *J* = 12.4, 5.1 Hz, 1H), 2.62 (s, 3H), 2.57 (d, *J* = 12.2 Hz, 1H), 2.54–2.51 (m, 1H), 2.47 (d, *J* = 13.7 Hz, 1H), 2.07 (t, *J* = 7.4 Hz, 2H), 2.04–1.97 (m, 2H), 1.80 (d, *J* = 11.3 Hz, 2H), 1.70–1.56 (m, 3H), 1.55–1.41 (m, 3H), 1.29 (dddd, *J* = 25.5, 16.6, 13.5, 6.7 Hz, 3H), 0.87–0.81 (m, 1H). ^{13}C NMR (151 MHz, DMSO) δ 172.93, 171.51, 162.09, 156.12, 144.98, 134.39, 133.37, 126.07, 120.00, 118.96, 118.44, 109.96, 106.50, 104.65, 69.17, 69.13, 69.08, 68.99, 68.58, 67.75, 60.42, 58.57, 56.86, 54.82, 54.31, 52.39, 47.99, 40.81, 37.85, 34.50, 27.65, 27.59, 27.43, 24.66. HRMS (ESI) calcd for $C_{36}H_{52}N_7O_6S_2$ $[M + H]^+$ 742.3415; found 742.3416. HPLC purity: 97.30%, t_R = 9.069 min.

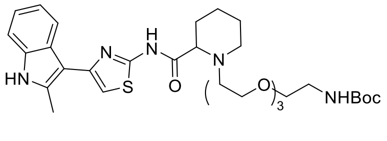

***tert*-butyl(2,2-diethoxy-2-(2-(2-((4-(2-methyl-1*H*-indol-3-yl)thiazol-2-yl)carbamol)piperidin-1-yl)ethoxy)ethyl)carbamate (S3b)**

Yellow solid (125 mg, 0.2 mmol, 42%). ^{1}H NMR (600 MHz, DMSO-d_6) δ 11.18 (s, 1H), 7.96 (d, *J* = 7.8 Hz, 1H), 7.31 (d, *J* = 7.9 Hz, 1H), 7.09 (s, 1H), 7.08–7.04 (m, 1H), 7.02 (td, *J* = 7.5, 7.1, 1.1 Hz, 1H), 6.70 (s, 1H), 3.62–3.44 (m, 16H), 3.31 (t, *J* = 6.1 Hz, 2H), 3.17 (s, 1H), 3.02 (q, *J* = 5.9 Hz, 2H), 2.62 (s, 3H), 1.67 (q, *J* = 13.3, 11.0 Hz, 2H), 1.64–1.51 (m, 2H), 1.36 (s, 9H).

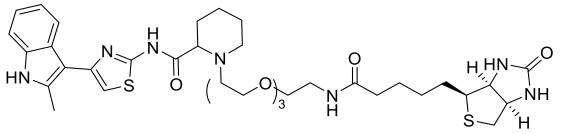

1-(2-(1,1-diethoxy-2-(5-((3*aS*,*4S*,6*aR*)-2-oxohexahydro-1*H*-thieno[3,4-d]imidazol-4-yl)pentanamido)ethoxy)ethyl)-*N*-(4-(2-methyl-1*H*-indol-3-yl)thiazol-2-yl)piperidine-2-carboxamide (Y0145, Bio-Y0747)

White solid (67 mg, 0.1 mmol, 56% yield). ^{1}H NMR (600 MHz, DMSO-d_6) δ 11.63 (s, 1H), 11.18 (s, 1H), 7.98–7.94 (m, 1H), 7.78 (t, *J* = 5.6 Hz, 1H), 7.31 (d, *J* = 7.9 Hz, 1H), 7.08 (s, 1H), 7.07–7.04 (m, 1H), 7.01 (td, *J* = 7.6, 7.1, 1.1 Hz, 1H), 6.41 (s, 1H), 6.35 (s, 1H), 4.31–4.26 (m, 1H), 4.12–4.07 (m, 1H), 3.55 (ddd, *J* = 12.2, 6.1, 1.5 Hz, 1H), 3.51 (d, *J* = 3.5 Hz, 5H), 3.45–3.42 (m, 2H), 3.42–3.39 (m, 2H), 3.33 (t, *J* = 5.9 Hz, 1H), 3.27 (dd, *J* = 8.3, 3.3 Hz, 1H), 3.13 (td, *J* = 11.8, 5.0 Hz, 3H), 3.09–3.04 (m, 1H), 2.80 (dd, *J* = 12.4, 5.1 Hz, 1H), 2.62 (m, 4H), 2.56 (d, *J* = 12.4 Hz, 1H), 2.26 (t, *J* = 9.2 Hz, 1H), 2.06–2.01 (m, 2H), 1.83 1.76 (m, 1H), 1.71–1.63 (m, 2H), 1.62–1.55 (m, 2H), 1.47 (ddtd, *J* = 23.0, 18.9, 9.9, 8.9, 5.1 Hz, 4H), 1.36 1.21 (m, 4H). ^{13}C NMR (151 MHz, DMSO) δ 171.46, 171.42, 162.07, 155.60, 145.07, 134.36, 133.40, 126.05, 119.99, 118.93, 118.45, 109.94, 106.42, 104.86, 69.10, 69.00, 68.86, 68.49, 67.35, 64.26, 60.40, 58.55, 54.79, 54.46, 50.38, 39.43, 39.21, 37.78, 34.45, 28.55, 27.56, 27.40, 24.62, 23.99,

21.71, 12.72. HRMS (ESI) calcd for $C_{36}H_{52}N_7O_6S_2$ $[M + H]^+$ 742.3415; found 742.3413. HPLC purity: 95.98%, t_R = 9.004 min.

NMR, HRMS and HPLC Spectra of Final Product

Compound **F0341**, ^{1}H NMR (500 MHz, DMSO-d_6)

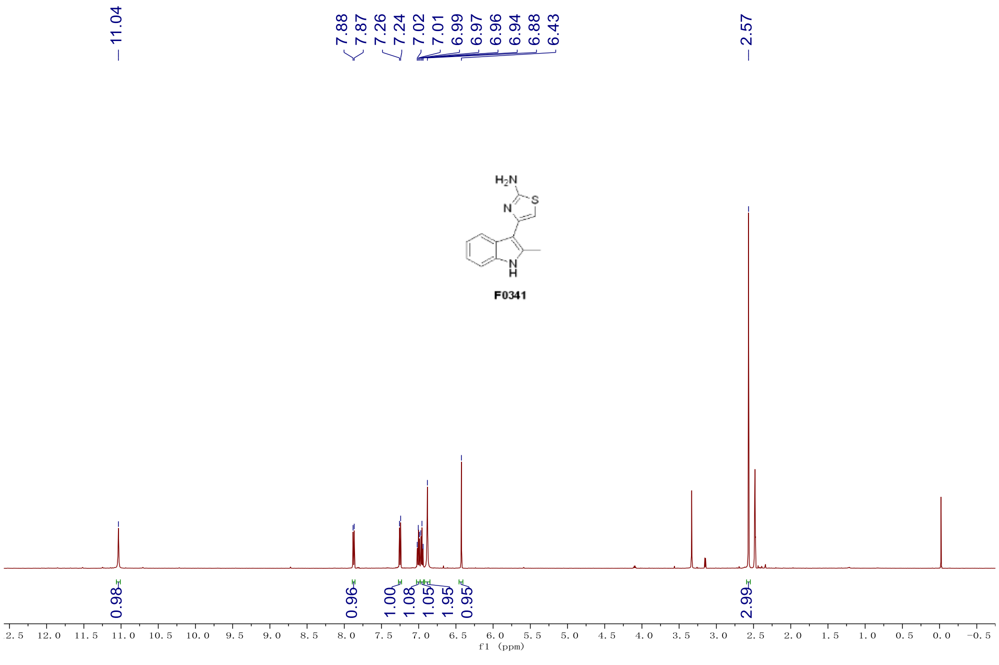

Compound **F0341**, HPLC

Single Injection Report Agilent

**Data file:**	YJH-F0341.dx		
**Sequence Name:**	YJH-F0341-2	**Project Name:**	1260
**Sample name:**	YJH-F0341	**Operator:**	SYSTEM
**Instrument:**	1260	**Injection date:**	2023-01-12 15:42:53+08:00
**Inj. volume:**	10.000 µL	**Location:**	P1-A1
**Acq. method:**	normal.amx	**Type:**	Sample
**Processing method:**	GC_LC Area Percent_DefaultMethod.pmx	**Sample amount:**	0.00
**Manually modified:**	Manual Integration		

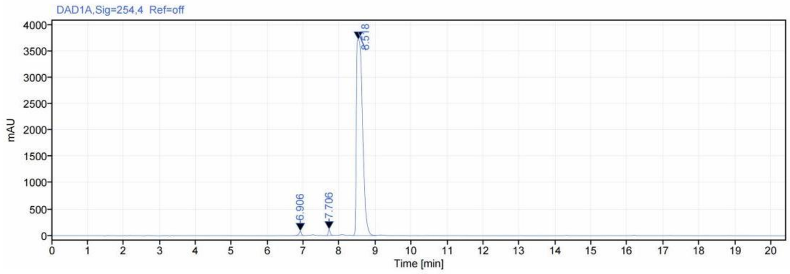

Signal: DAD1A,Sig=254,4 Ref=off

RT [min]	Type	Width [min]	Area	Height	Area%	Name
6.906	BB	0.45	397.41	82.63	0.88	
7.706	BV	0.21	433.05	114.11	0.96	
8.518	VV	0.67	44379.45	3721.84	98.16	
		**Sum**	45209.91		100.00	

Compound **Y0222**, ^{1}H-NMR (500 MHz, DMSO-d_6)

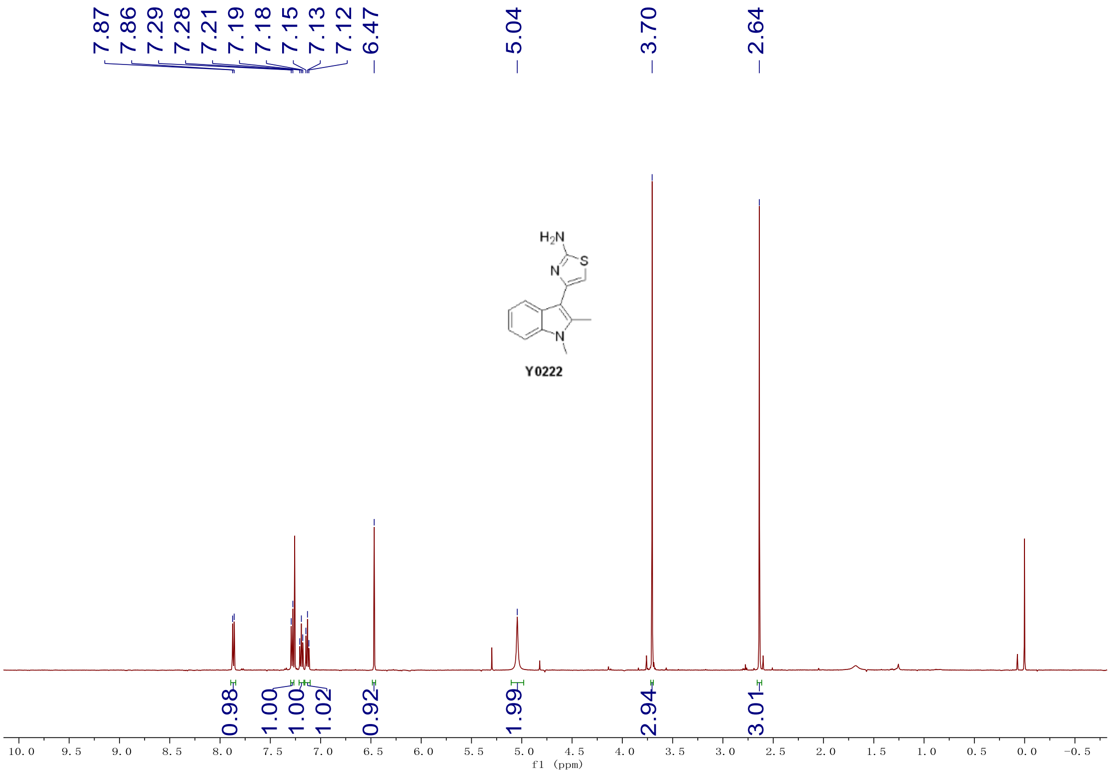

Compound **Y0222**, ^{13}C NMR (126 MHz, DMSO-d_6)

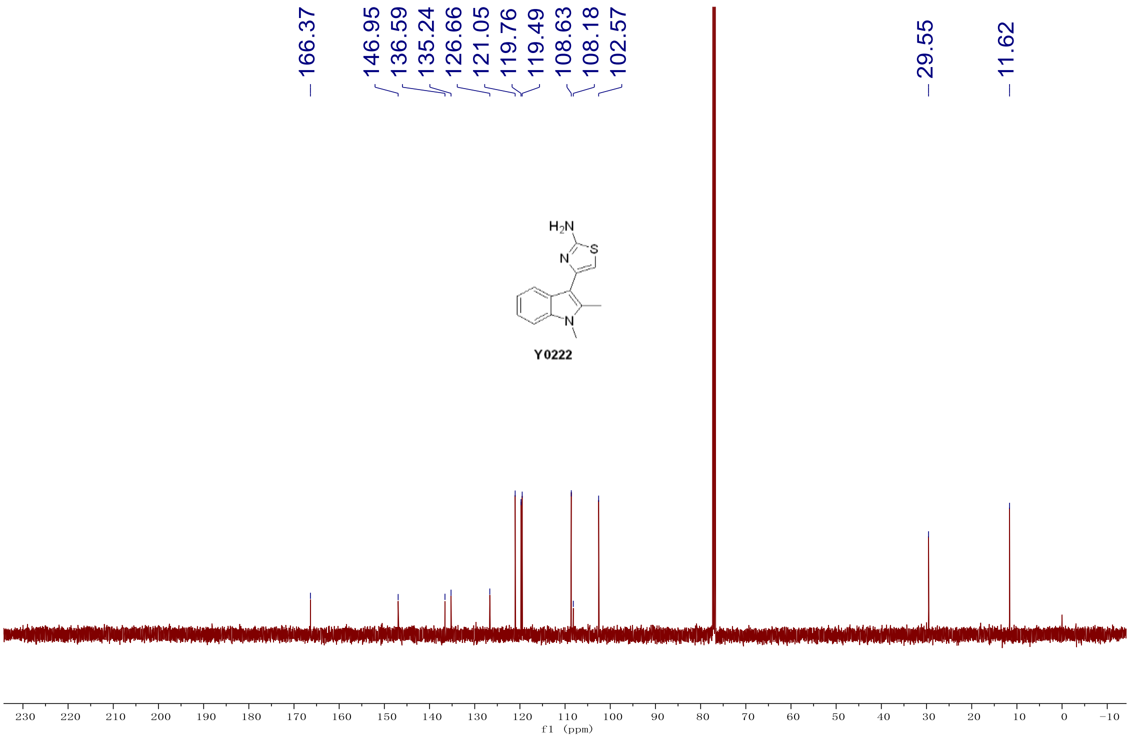

Compound **Y0222**, HRMS (ESI)

Mass Spectrum SmartFormula Report

Analysis Info

Analysis Name	D:\Data\SHUJVFENXI\DINGKE-GROUP\2021187-YJH-02-22-P_RA7_01_33260.d	Acquisition Date	12/5/2022 4:47:33 PM
Method	20150915.m	Operator	BDAL@DE
Sample Name	2021187-YJH-02-22-P	Instrument / Ser#	maXis 4G 21240
Comment			

Acquisition Parameter

Source Type	ESI	Ion Polarity	Positive	Set Nebulizer	1.0 Bar
Focus	Not active	Set Capillary	3000 V	Set Dry Heater	220 °C
Scan Begin	50 m/z	Set End Plate Offset	-500 V	Set Dry Gas	6.0 l/min
Scan End	1500 m/z	Set Collision Cell RF	600.0 Vpp	Set Divert Valve	Waste

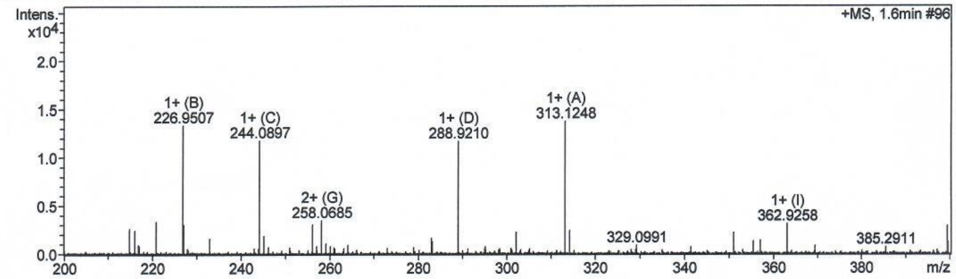

Meas. m/z	#	Formula	Score	m/z	err [ppm]	Mean err [ppm]	mSigma	rdb	e⁻ Conf	N-Rule
244.0897	1	C 13 H 14 N 3 S	100.00	244.0903	2.3	2.4	7.9	8.5	even	ok

Compound **Y0222**, HPLC

Single Injection Report

Agilent

**Data file:**	YJH-02-22-P.dx		
**Sequence Name:**	YJH-02-22-P	**Project Name:**	1260
**Sample name:**	YJH-02-22-P	**Operator:**	SYSTEM
**Instrument:**	1260	**Injection date:**	2022-12-03 21:54:15+08:00
**Inj. volume:**	10.000 µL	**Location:**	P1-D2
**Acq. method:**	normal.amx	**Type:**	Sample
**Processing method:**	GC_LC Area Percent_DefaultMethod.pmx	**Sample amount:**	0.00
**Manually modified:**	Manual Integration		

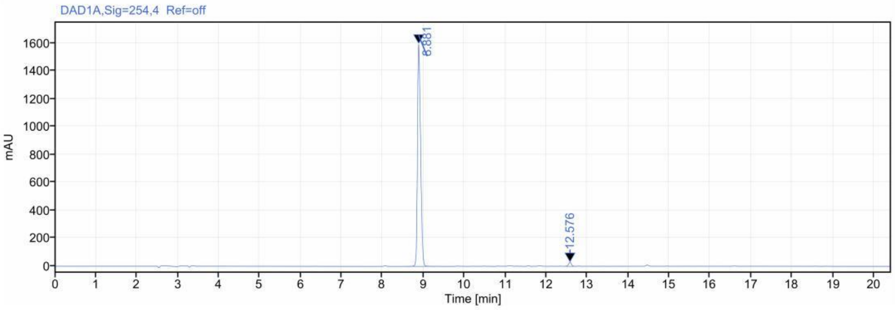

Signal: DAD1A,Sig=254,4 Ref=off

RT [min]	Type	Width [min]	Area	Height	Area%	Name
8.881	BV	0.71	8449.81	1594.85	98.30	
12.576	VV	0.31	145.78	30.19	1.70	
		**Sum**	8595.59		100.00	

Compound **Y0257**, ^{1}H-NMR (500 MHz, Acetonitrile-d_3)

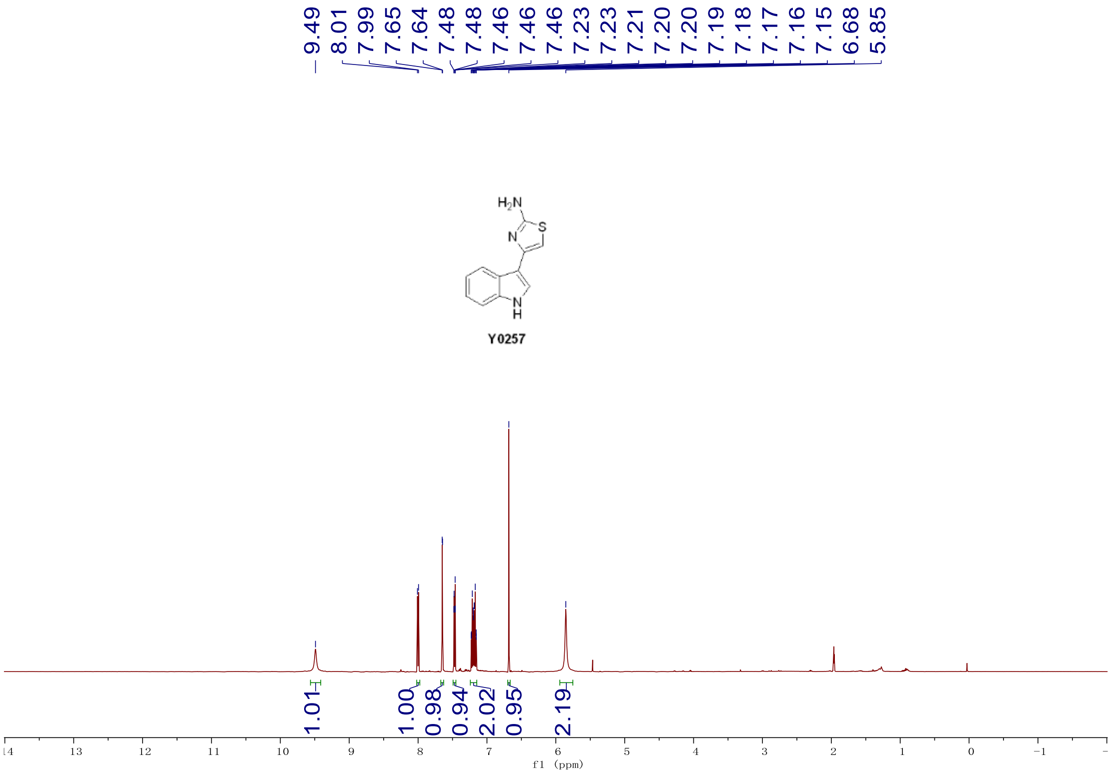

Compound **Y0257**, ^{13}C NMR (126 MHz, Acetonitrile-d_3)

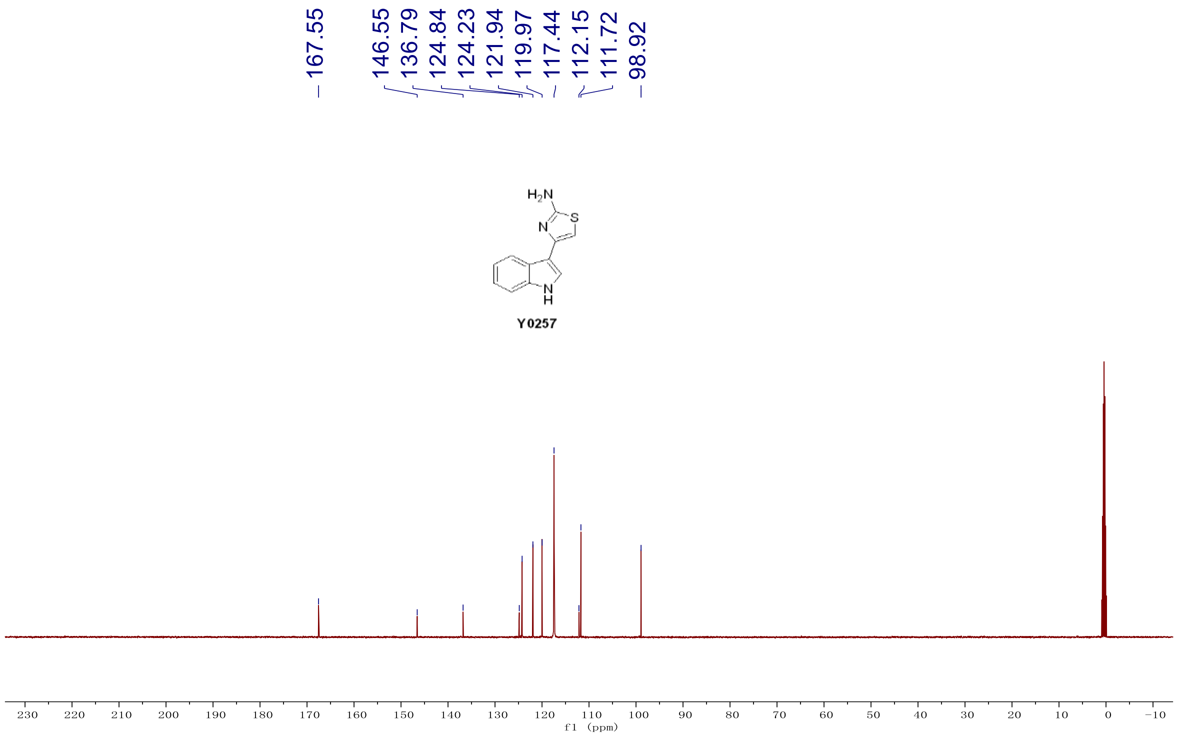

Compound **Y0257**, HRMS (ESI)

Mass Spectrum SmartFormula Report

Analysis Info

Analysis Name	D:\Data\SHUJVFENXI\DINGKE-GROUP\2021187-Yyjh-02-57-P_BC2_01_42545.d	Acquisition Date	3/12/2024 10:43:11 AM
Method	6min.m	Operator	BDAL@DE
Sample Name	2021187-Yyjh-02-57-P	Instrument / Ser#	maXis 4G 21240
Comment			

Acquisition Parameter

Source Type	ESI	Ion Polarity	Positive	Set Nebulizer	1.0 Bar
Focus	Not active	Set Capillary	3000 V	Set Dry Heater	220 °C
Scan Begin	50 m/z	Set End Plate Offset	-500 V	Set Dry Gas	6.0 l/min
Scan End	1500 m/z	Set Collision Cell RF	600.0 Vpp	Set Divert Valve	Waste

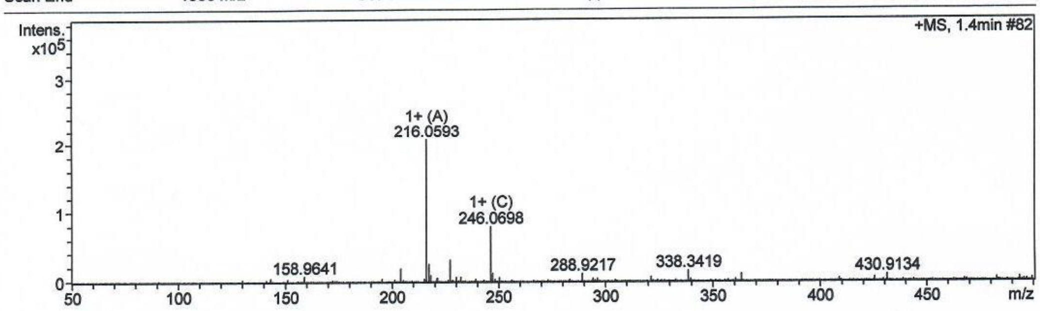

Meas. m/z	#	Formula	Score	m/z	err [ppm]	Mean err [ppm]	mSigma	rdb	e⁻ Conf	N-Rule
216.0593	1	C 11 H 10 N 3 S	100.00	216.0590	-1.5	-1.3	11.3	8.5	even	ok

Compound **Y0257**, HPLC

Single Injection Report

Agilent

**Data file:**	YJH-02-57-P.dx		
**Sequence Name:**	YJH-02-57-P	**Project Name:**	1260
**Sample name:**	YJH-02-57-P	**Operator:**	SYSTEM
**Instrument:**	1260	**Injection date:**	2022-12-18 22:35:38+08:00
**Inj. volume:**	10.000 µL	**Location:**	P1-D1
**Acq. method:**	normal.amx	**Type:**	Sample
**Processing method:**	GC_LC Area Percent_DefaultMethod.pmx	**Sample amount:**	0.00
**Manually modified:**	Manual Integration		

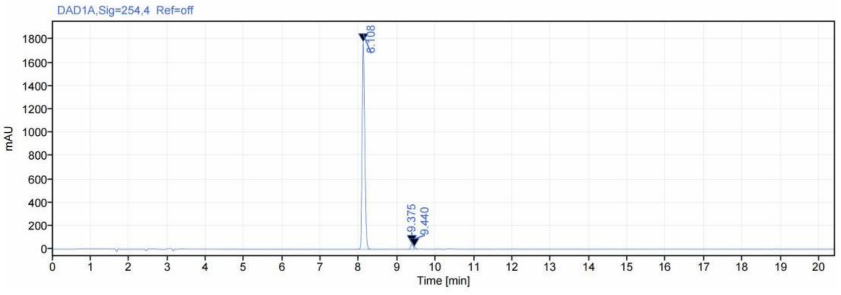

Signal: DAD1A,Sig=254,4 Ref=off

RT [min]	Type	Width [min]	Area	Height	Area%	Name
8.108	BV	0.52	8477.07	1778.36	97.12	
9.375	BV	0.15	189.08	46.77	2.17	
9.440	VV	0.12	61.96	20.03	0.71	
		Sum	8728.10		100.00	

Compound **Y0253**, ^{1}H NMR (500 MHz, DMSO-d_6)

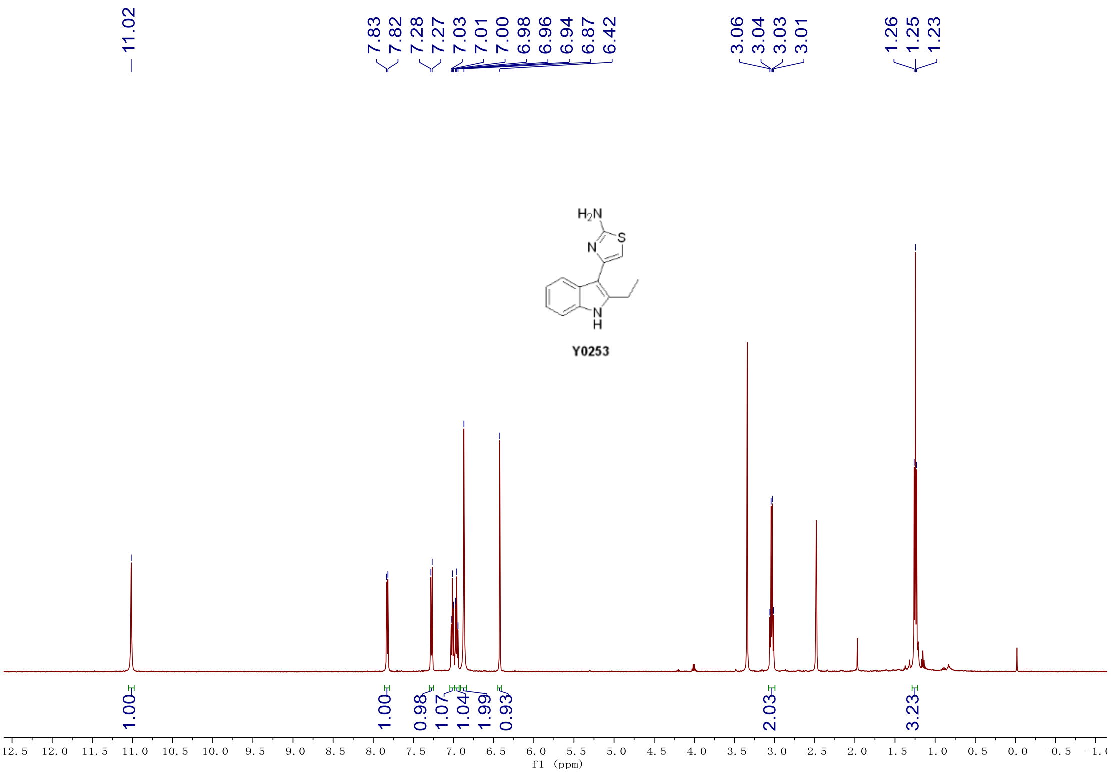

Compound **Y0253**, ^{13}C NMR (126 MHz, DMSO-d_6)

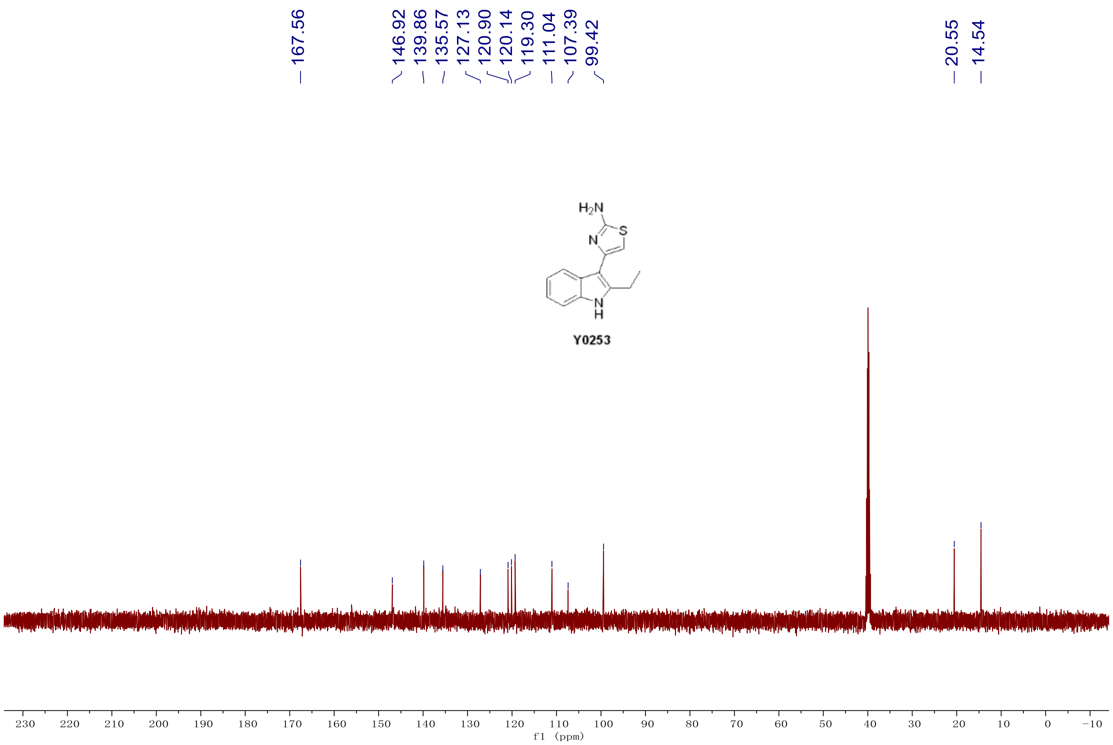

Compound **Y0253**, HRMS (ESI)

Mass Spectrum SmartFormula Report

Analysis Info

Analysis Name	D:\Data\SHUJVFENXI\DINGKE-GROUP\2021187-YJH-02-53-P_BB3_01_34071.d	Acquisition Date	2/8/2023 12:08:43 PM
Method	20150915.m	Operator	BDAL@DE
Sample Name	2021187-YJH-02-53-P	Instrument / Ser#	maXis 4G 21240
Comment			

Acquisition Parameter

Source Type	ESI	Ion Polarity	Positive	Set Nebulizer	1.0 Bar
Focus	Not active	Set Capillary	3000 V	Set Dry Heater	220 °C
Scan Begin	50 m/z	Set End Plate Offset	-500 V	Set Dry Gas	6.0 l/min
Scan End	1500 m/z	Set Collision Cell RF	600.0 Vpp	Set Divert Valve	Waste

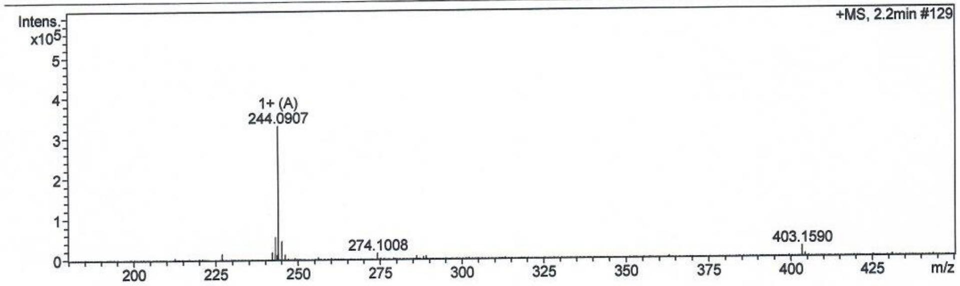

Meas. m/z	#	Formula	Score	m/z	err [ppm]	Mean err [ppm]	mSigma	rdb	e⁻ Conf	N-Rule
244.0907	1	C 13 H 14 N 3 S	100.00	244.0903	-1.6	-1.3	14.3	8.5	even	ok

Compound **Y0253**, HPLC

Single Injection Report

Agilent

**Data file:**	YJH-02-53-P.dx		
**Sequence Name:**	YJH-02-53-P	**Project Name:**	1260
**Sample name:**	YJH-02-53-P	**Operator:**	SYSTEM
**Instrument:**	1260	**Injection date:**	2022-12-15 21:51:24+08:00
**Inj. volume:**	10.000 µL	**Location:**	P1-E2
**Acq. method:**	normal.amx	**Type:**	Sample
**Processing method:**	GC_LC Area Percent_DefaultMethod.pmx	**Sample amount:**	0.00
**Manually modified:**	Manual Integration		

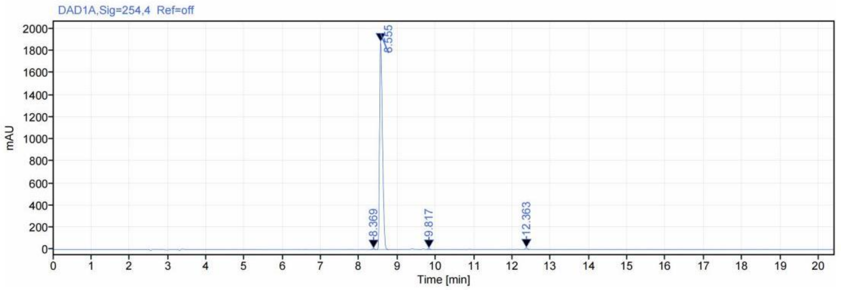

Signal: DAD1A,Sig=254,4 Ref=off

RT [min]	Type	Width [min]	Area	Height	Area%	Name
8.369	BV	0.25	46.17	10.85	0.46	
8.555	VB	0.50	9825.16	1882.08	98.37	
9.817	VV	0.16	45.05	10.85	0.45	
12.363	BV	0.21	71.97	17.13	0.72	
		**Sum**	9988.35		100.00	

Compound **Y0226**, ^{1}H NMR (500 MHz, DMSO-d_6)

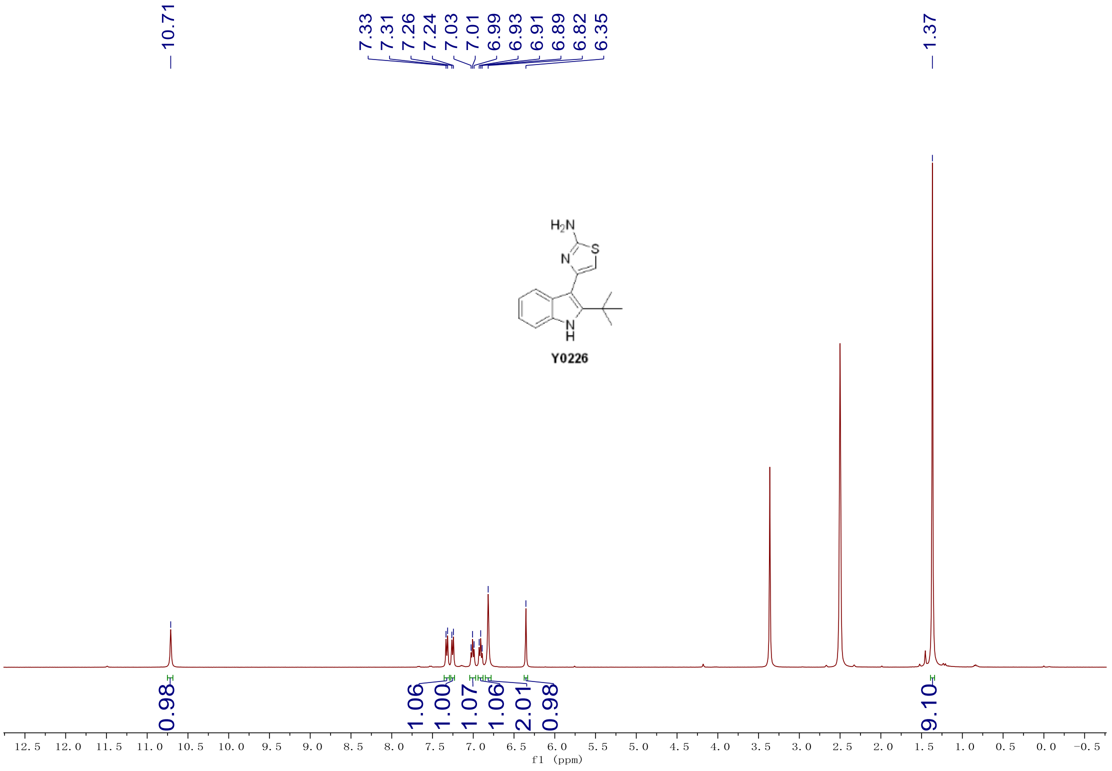

Compound **Y0226**, ^{13}C NMR (126 MHz, DMSO-d_6)

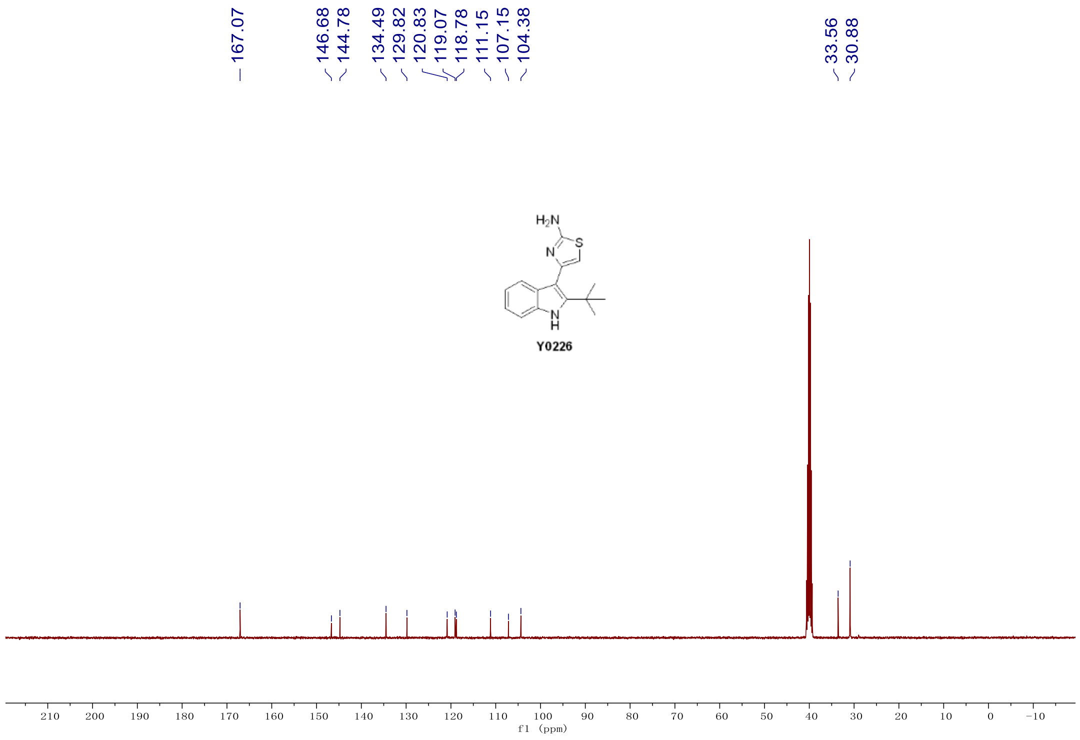

Compound **Y0226**, HRMS (ESA)

Mass Spectrum SmartFormula Report

**Analysis Info**		Acquisition Date	2/8/2023 11:36:11 AM
Analysis Name	D:\Data\SHUJVFENXI\DINGKE-GROUP\2021187-YJH-02-26-P_BA6_01_34066.d		
Method	20150915.m	Operator	BDAL@DE
Sample Name	2021187-YJH-02-26-P	Instrument / Ser#	maXis 4G 21240
Comment			

Acquisition Parameter

Source Type	ESI	Ion Polarity	Positive	Set Nebulizer	1.0 Bar
Focus	Not active	Set Capillary	3000 V	Set Dry Heater	220 °C
Scan Begin	50 m/z	Set End Plate Offset	-500 V	Set Dry Gas	6.0 l/min
Scan End	1500 m/z	Set Collision Cell RF	600.0 Vpp	Set Divert Valve	Waste

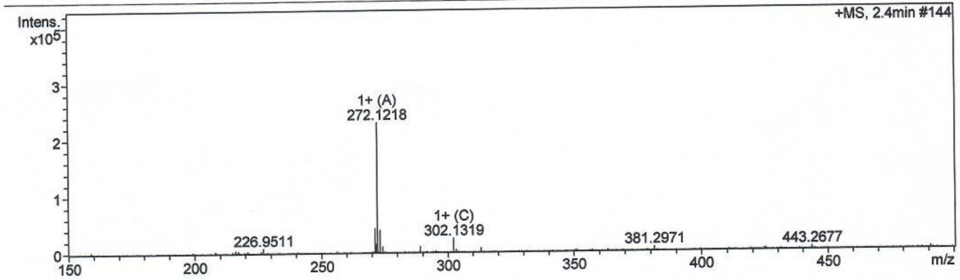

Meas. m/z	#	Formula	Score	m/z	err [ppm]	Mean err [ppm]	mSigma	rdb	e⁻ Conf	N-Rule
272.1218	1	C 15 H 18 N 3 S	100.00	272.1216	-0.7	-0.5	9.0	8.5	even	ok

Compound **Y0226**, HPLC

Single Injection Report

Agilent

**Data file:**	YJH-02-26-P.dx		
**Sequence Name:**	YJH-02-26-P	**Project Name:**	1260
**Sample name:**	YJH-02-26-P	**Operator:**	SYSTEM
**Instrument:**	1260	**Injection date:**	2022-12-06 20:42:32+08:00
**Inj. volume:**	10.000 µL	**Location:**	P1-E1
**Acq. method:**	normal.amx	**Type:**	Sample
**Processing method:**	GC_LC Area Percent_DefaultMethod.pmx	**Sample amount:**	0.00
**Manually modified:**	Manual Integration		

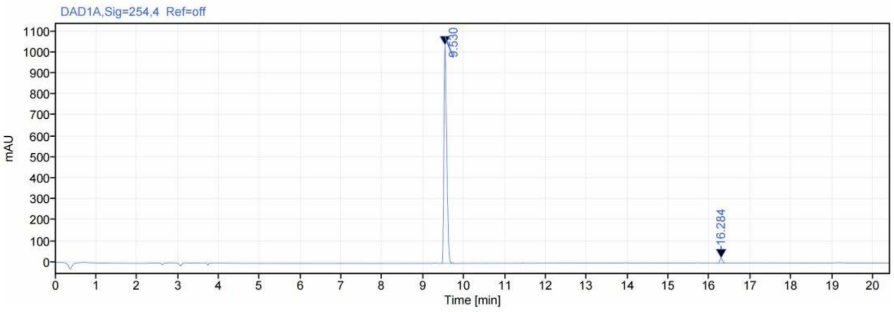

Signal: DAD1A,Sig=254,4 Ref=off

RT [min]	Type	Width [min]	Area	Height	Area%	Name
9.530	VB	0.48	4966.96	1039.59	98.11	
16.284	VV	0.26	95.72	22.22	1.89	
		**Sum**	5062.68		100.00	

Compound **Y0227**, (500 MHz, DMSO-d_6)

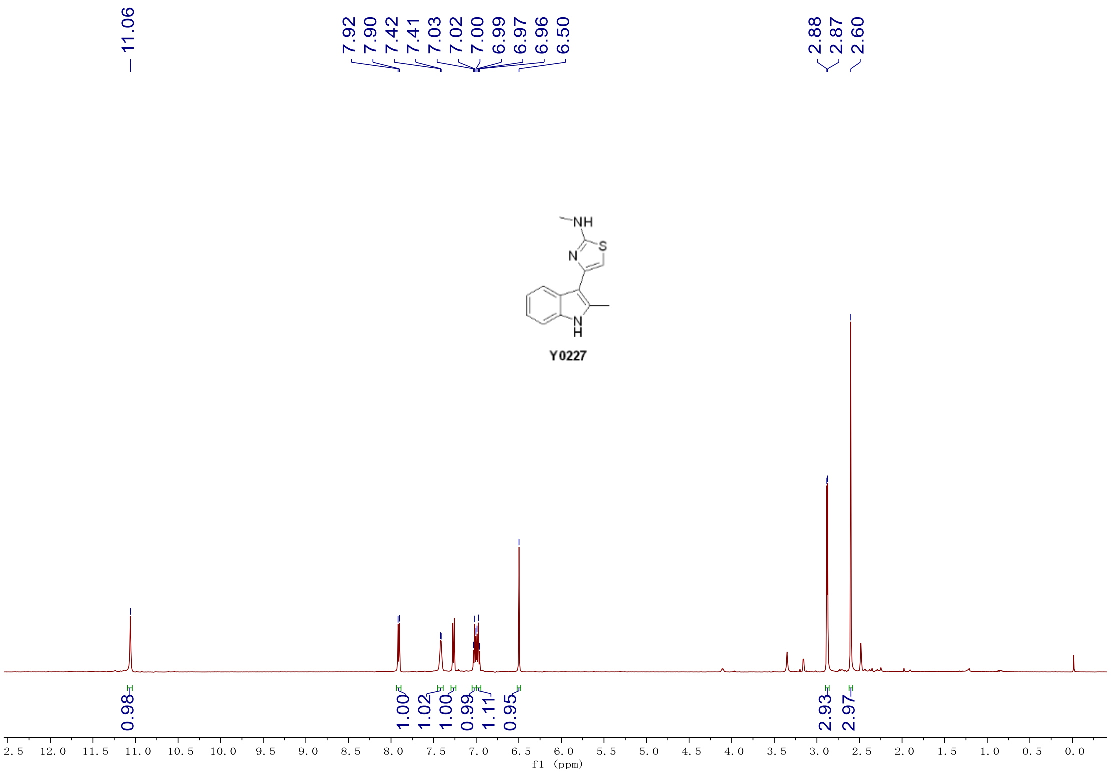

Compound **Y0227**, ^{13}C NMR (126 MHz, DMSO-d_6)

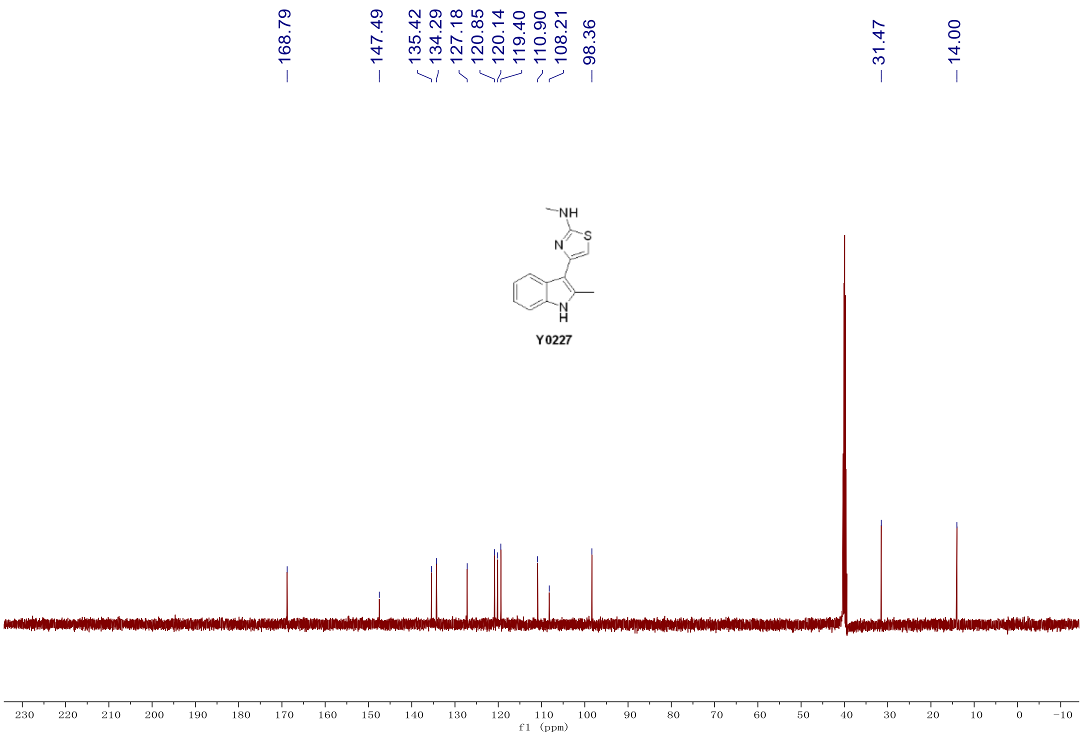

Compound **Y0227**, HRMS (ESI)

Mass Spectrum SmartFormula Report

Analysis Info

Analysis Name	D:\Data\SHUJVFENXI\DINGKE-GROUP\2021187-YJH-02-27-P_BA7_01_34067.d	Acquisition Date	2/8/2023 11:42:45 AM
Method	20150915.m	Operator	BDAL@DE
Sample Name	2021187-YJH-02-27-P	Instrument / Ser#	maXis 4G 21240
Comment			

Acquisition Parameter

Source Type	ESI	Ion Polarity	Positive	Set Nebulizer	1.0 Bar
Focus	Not active	Set Capillary	3000 V	Set Dry Heater	220 °C
Scan Begin	50 m/z	Set End Plate Offset	-500 V	Set Dry Gas	6.0 l/min
Scan End	1500 m/z	Set Collision Cell RF	600.0 Vpp	Set Divert Valve	Waste

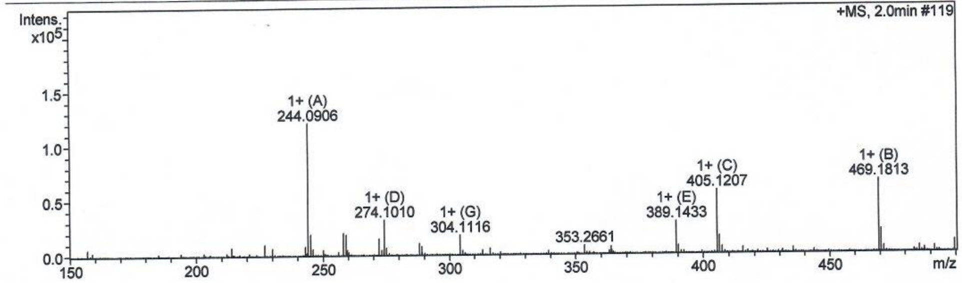

Meas. m/z	#	Formula	Score	m/z	err [ppm]	Mean err [ppm]	mSigma	rdb	e⁻ Conf	N-Rule
244.0906	1	C 13 H 14 N 3 S	100.00	244.0903	-1.2	-0.9	5.9	8.5	even	ok

Compound **Y0227**, HPLC

Single Injection Report

Agilent

**Data file:**	YJH-02-27-P.dx		
**Sequence Name:**	YJH-02-27-P	**Project Name:**	1260
**Sample name:**	YJH-02-27-P	**Operator:**	SYSTEM
**Instrument:**	1260	**Injection date:**	2022-12-06 21:07:45+08:00
**Inj. volume:**	10.000 µL	**Location:**	P1-E2
**Acq. method:**	normal.amx	**Type:**	Sample
**Processing method:**	GC_LC Area Percent_DefaultMethod.pmx	**Sample amount:**	0.00
**Manually modified:**	Manual Integration		

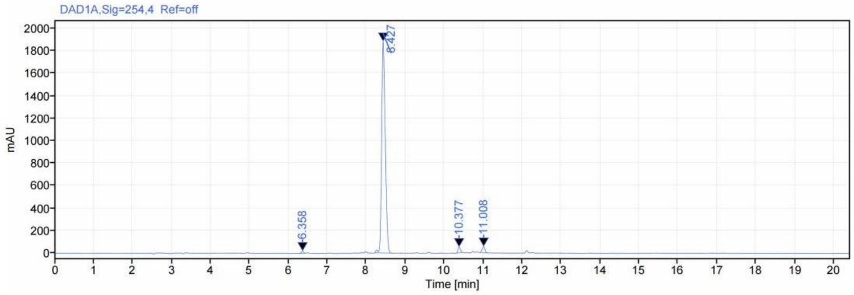

Signal: DAD1A,Sig=254,4 Ref=off

RT [min]	Type	Width [min]	Area	Height	Area%	Name
6.358	BV	0.21	78.26	20.54	0.61	
8.427	MB m	0.54	12170.81	1880.34	95.58	
10.377	BM m	0.18	218.82	50.39	1.72	
11.008	MB m	0.22	265.38	54.42	2.08	
		**Sum**	12733.26		100.00	

Compound **Y0223**, ^{1}H NMR (500 MHz, Chloroform-*d*)

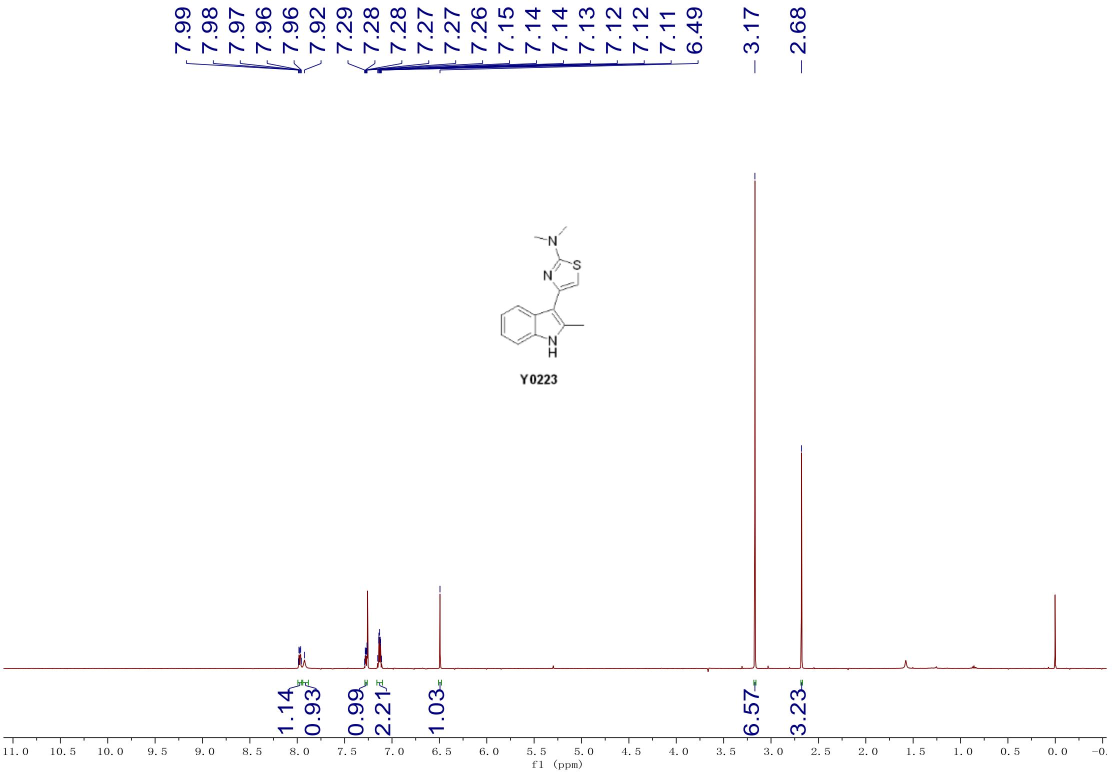

Compound **Y0223**, ^{13}C NMR (126 MHz, Chloroform-*d*)

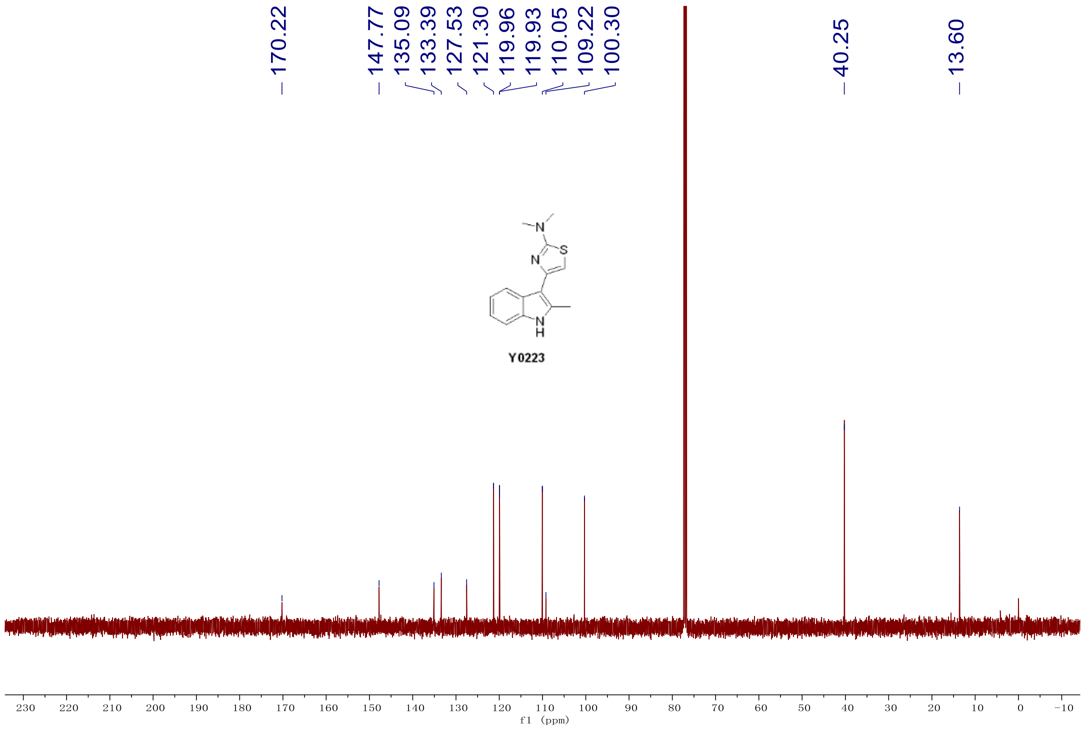

Compound **Y0223**, HRMS (ESI)

Mass Spectrum SmartFormula Report

Analysis Info

Analysis Name	D:\Data\SHUJVFENXI\DINGKE-GROUP\2021187-YJH-02-23-P_RA8_01_33261.d	Acquisition Date	12/5/2022 4:53:51 PM
Method	20150915.m	Operator	BDAL@DE
Sample Name	2021187-YJH-02-23-P	Instrument / Ser#	maXis 4G 21240
Comment			

Acquisition Parameter

Source Type	ESI	Ion Polarity	Positive	Set Nebulizer	1.0 Bar
Focus	Not active	Set Capillary	3000 V	Set Dry Heater	220 °C
Scan Begin	50 m/z	Set End Plate Offset	-500 V	Set Dry Gas	6.0 l/min
Scan End	1500 m/z	Set Collision Cell RF	600.0 Vpp	Set Divert Valve	Waste

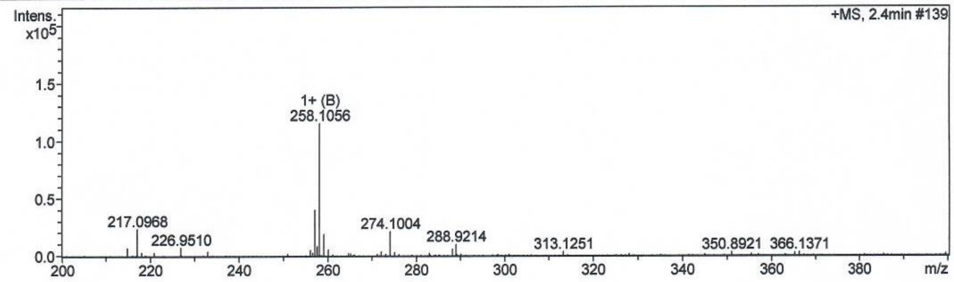

Meas. m/z	#	Formula	Score	m/z	err [ppm]	Mean err [ppm]	mSigma	rdb	e⁻ Conf	N-Rule
258.1056	1	C 14 H 16 N 3 S	100.00	258.1059	1.3	1.7	6.1	8.5	even	ok

Compound **Y0223**, HPLC

Single Injection Report

Agilent

**Data file:**	YJH-02-23-P.dx		
**Sequence Name:**	YJH-02-23-P	**Project Name:**	1260
**Sample name:**	YJH-02-23-P	**Operator:**	SYSTEM
**Instrument:**	1260	**Injection date:**	2022-12-03 22:19:26+08:00
**Inj. volume:**	10.000 µL	**Location:**	P1-D3
**Acq. method:**	normal.amx	**Type:**	Sample
**Processing method:**	GC_LC Area Percent_DefaultMethod.pmx	**Sample amount:**	0.00
**Manually modified:**	Manual Integration		

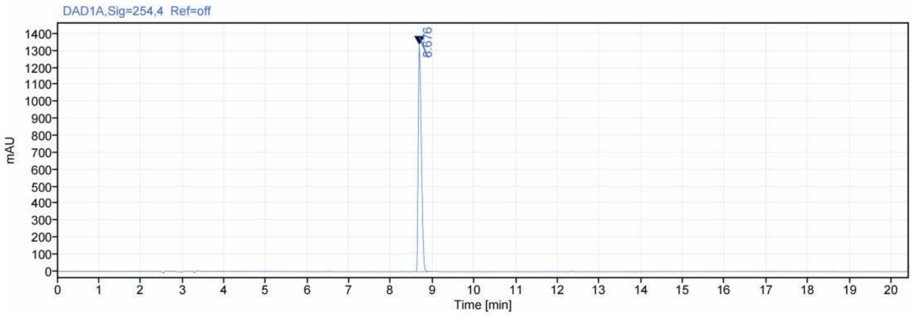

Signal: DAD1A,Sig=254,4 Ref=off

RT [min]	Type	Width [min]	Area	Height	Area%	Name
8.676	VV	0.30	7035.72	1334.35	100.00	
		**Sum**	7035.72		100.00	

Compound **Y0228**, ^{1}H NMR (500 MHz, DMSO-d_6)

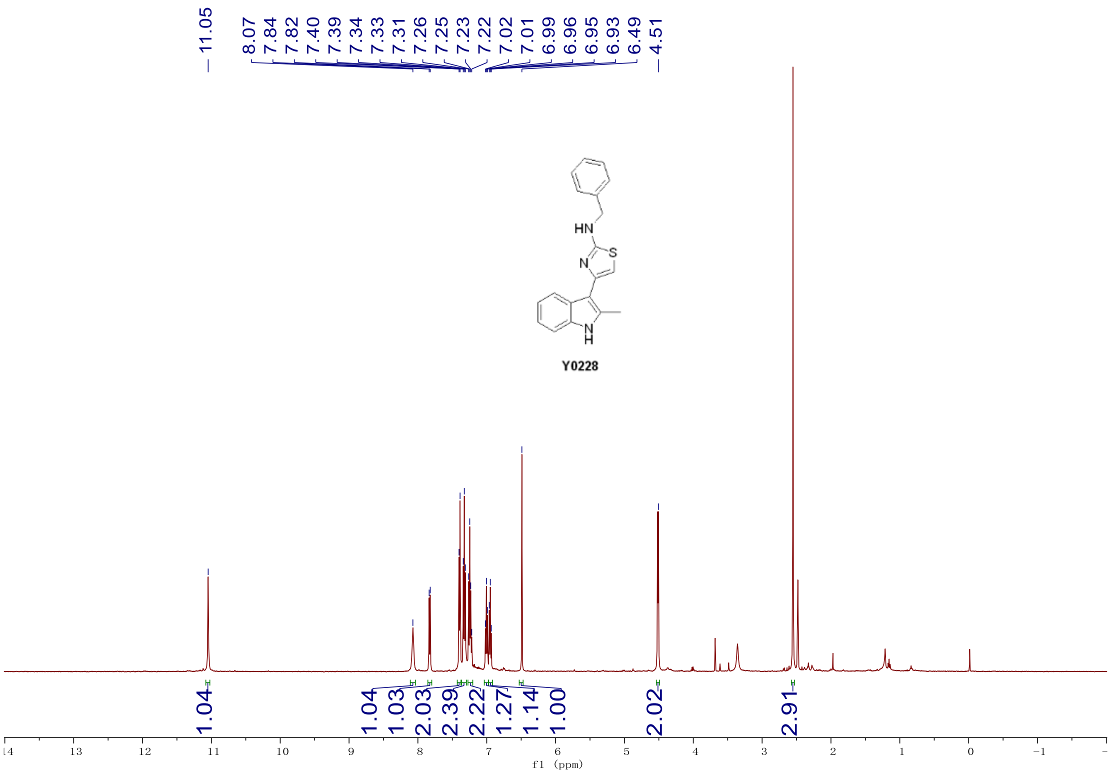

Compound **Y0228**, ^{13}C NMR (126 MHz, DMSO-d_6)

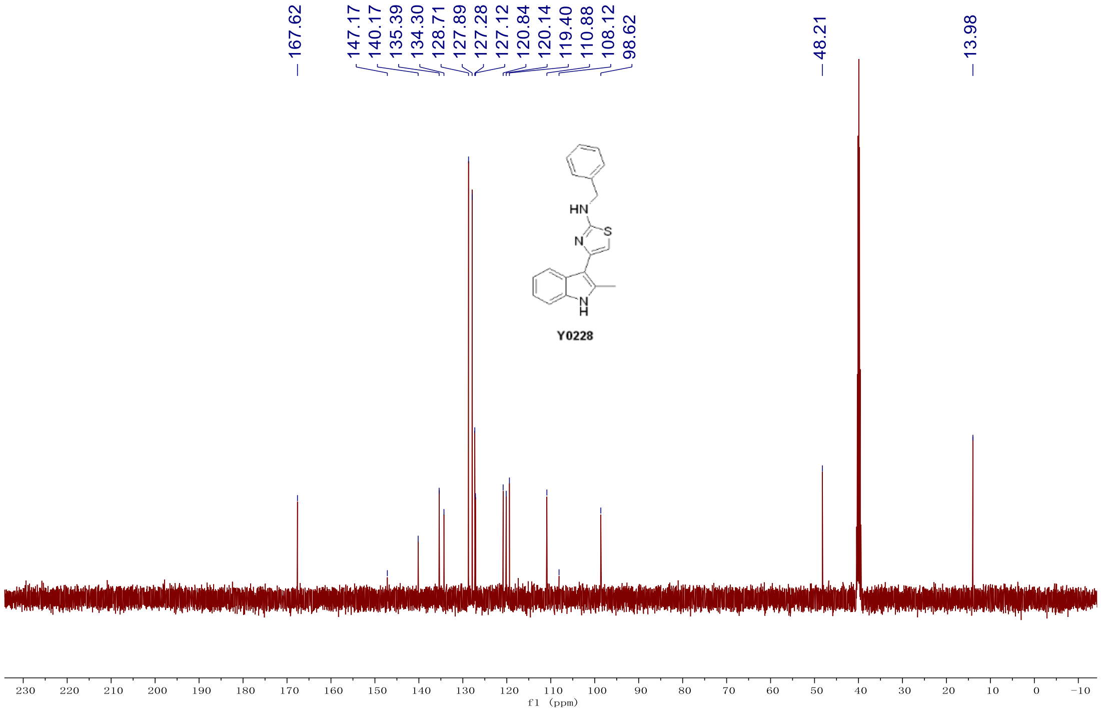

Compound **Y0228**, HRMS (ESI）

Mass Spectrum SmartFormula Report

Analysis Info

Analysis Name	D:\Data\SHUJVFENXI\DINGKE-GROUP\2021187-YJH-02-28-P_BA8_01_34068.d	Acquisition Date	2/8/2023 11:49:18 AM
Method	20150915.m	Operator	BDAL@DE
Sample Name	2021187-YJH-02-28-P	Instrument / Ser#	maXis 4G 21240
Comment			

Acquisition Parameter

Source Type	ESI	Ion Polarity	Positive	Set Nebulizer	1.0 Bar
Focus	Not active	Set Capillary	3000 V	Set Dry Heater	220 °C
Scan Begin	50 m/z	Set End Plate Offset	-500 V	Set Dry Gas	6.0 l/min
Scan End	1500 m/z	Set Collision Cell RF	600.0 Vpp	Set Divert Valve	Waste

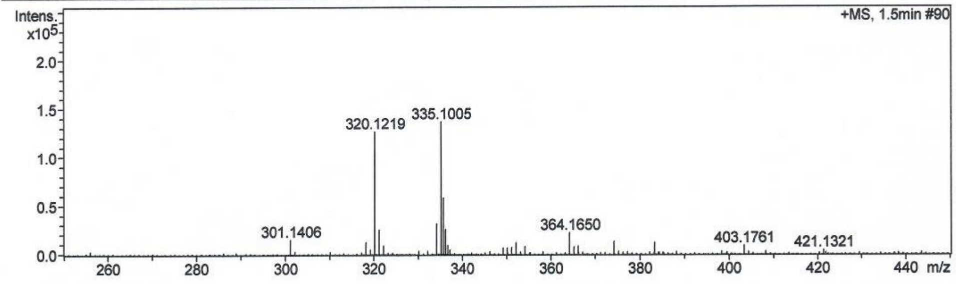

Meas. m/z	#	Formula	Score	m/z	err [ppm]	Mean err [ppm]	mSigma	rdb	e⁻ Conf	N-Rule
320.1219	1	C 19 H 18 N 3 S	100.00	320.1216	-0.9	-0.9	15.2	12.5	even	ok

Compound **Y0228**, HPLC

Single Injection Report

Agilent

**Data file:**	YJH-02-28-P.dx		
**Sequence Name:**	YJH-02-28-P	**Project Name:**	1260
**Sample name:**	YJH-02-28-P	**Operator:**	SYSTEM
**Instrument:**	1260	**Injection date:**	2022-12-06 21:32:56+08:00
**Inj. volume:**	10.000 µL	**Location:**	P1-E3
**Acq. method:**	normal.amx	**Type:**	Sample
**Processing method:**	GC_LC Area Percent_DefaultMethod.pmx	**Sample amount:**	0.00
**Manually modified:**	Manual Integration		

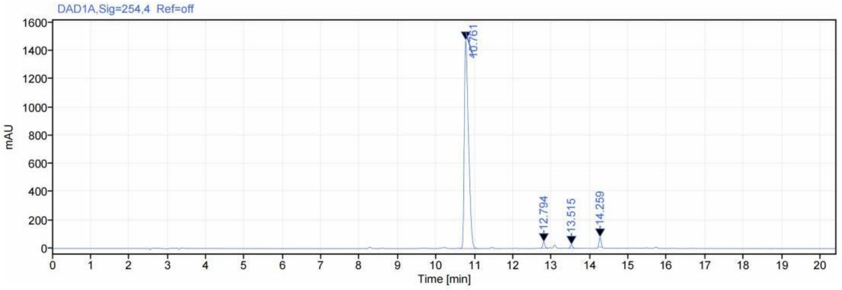

Signal: DAD1A,Sig=254,4 Ref=off

RT [min]	Type	Width [min]	Area	Height	Area%	Name
10.761	VV	0.54	11157.59	1475.03	95.39	
12.794	BV	0.19	192.54	45.14	1.65	
13.515	BV	0.18	104.94	25.77	0.90	
14.259	MM m	0.11	241.77	68.78	2.07	
		**Sum**	11696.84		100.00	

Compound **Y0318**, (500 MHz, DMSO-d_6)

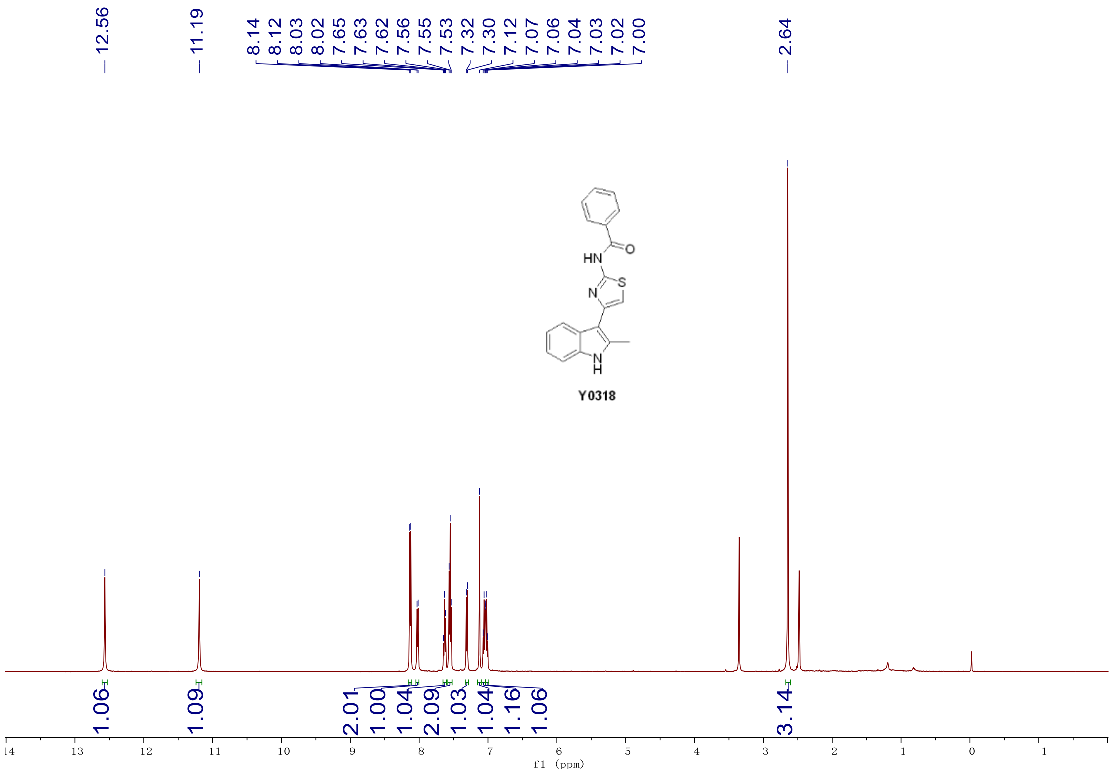

Compound **Y0318**, (126 MHz, DMSO-d_6)

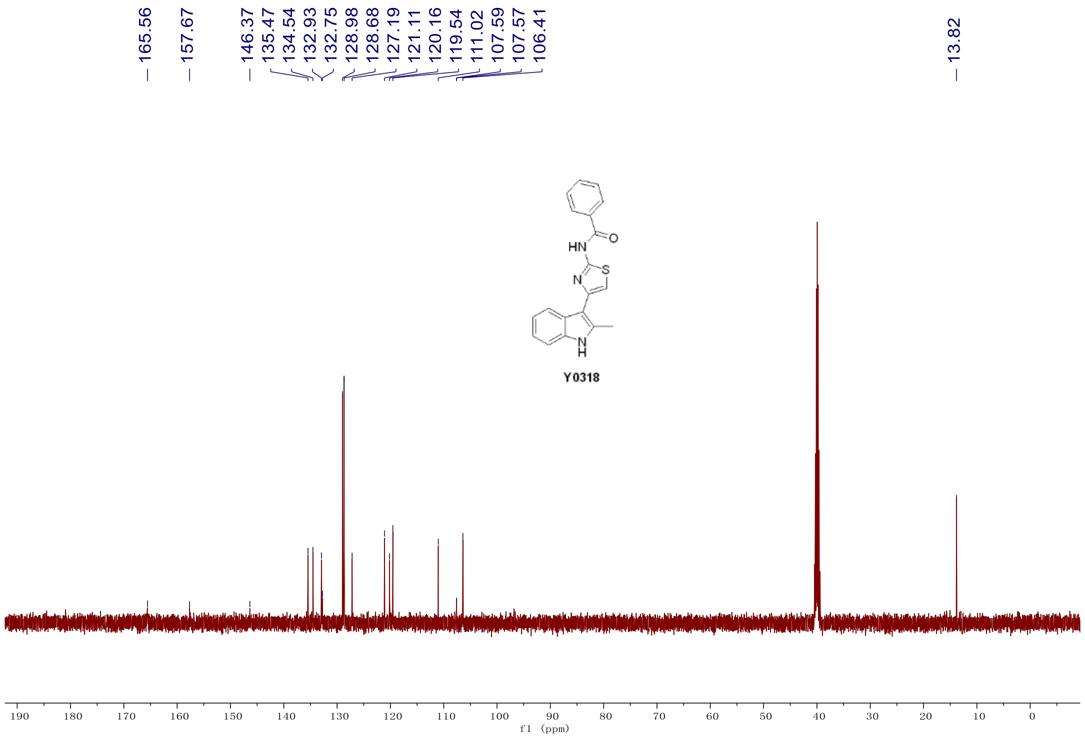

Compound **Y0318**, HRMS (ESI)

Mass Spectrum SmartFormula Report

Analysis Info

Analysis Name D:\Data\SHUJVFENXI\DINGKE-GROUP\2021187-YJH-03-18-P_RA5_01_34185.d
Method 20150915.m
Sample Name 2021187-YJH-03-18-P
Comment

Acquisition Date 2/13/2023 3:41:51 PM
Operator BDAL@DE
Instrument / Ser# maXis 4G 21240

Acquisition Parameter

Source Type	ESI	Ion Polarity	Positive	Set Nebulizer	1.0 Bar
Focus	Not active	Set Capillary	3000 V	Set Dry Heater	220 °C
Scan Begin	50 m/z	Set End Plate Offset	-500 V	Set Dry Gas	6.0 l/min
Scan End	1500 m/z	Set Collision Cell RF	600.0 Vpp	Set Divert Valve	Waste

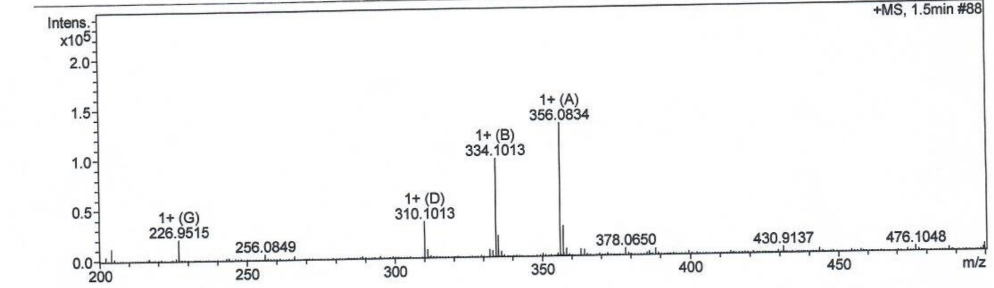

Meas. m/z	#	Formula	Score	m/z	err [ppm]	Mean err [ppm]	mSigma	rdb	e⁻ Conf	N-Rule
334.1013	1	C 19 H 16 N 3 O S	100.00	334.1009	-1.3	-1.4	10.3	13.5	even	ok
356.0834	1	C 19 H 15 N 3 Na O S	100.00	356.0828	-1.8	-1.7	8.6	13.5	even	ok

Compound **Y0318**, HPLC

Single Injection Report

Agilent

**Data file:**	YJH-03-18-4P.dx		
**Sequence Name:**	YJH-03-18-4P	**Project Name:**	1260
**Sample name:**	YJH-03-18-4P	**Operator:**	SYSTEM
**Instrument:**	1260	**Injection date:**	2023-02-10 22:31:12+08:00
**Inj. volume:**	10.000 µL	**Location:**	P1-D1
**Acq. method:**	normal.amx	**Type:**	Sample
**Processing method:**	GC_LC Area Percent_DefaultMethod.pmx	**Sample amount:**	0.00
**Manually modified:**	Manual Integration		

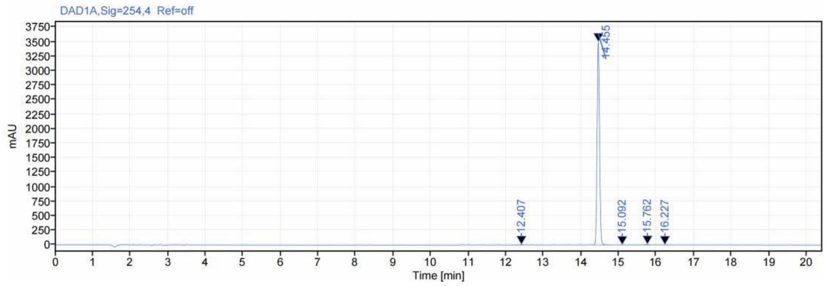

Signal: DAD1A,Sig=254,4 Ref=off

RT [min]	Type	Width [min]	Area	Height	Area%	Name
12.407	BV	0.31	40.33	7.83	0.25	
14.455	VB	0.48	15982.98	3499.51	99.13	
15.092	VB	0.18	8.65	1.97	0.05	
15.762	VB	0.29	65.92	10.62	0.41	
16.227	VB	0.30	25.56	4.36	0.16	
		**Sum**	16123.45		100.00	

Compound **Y0346**, ^{1}H NMR (600 MHz, DMSO-d_6)

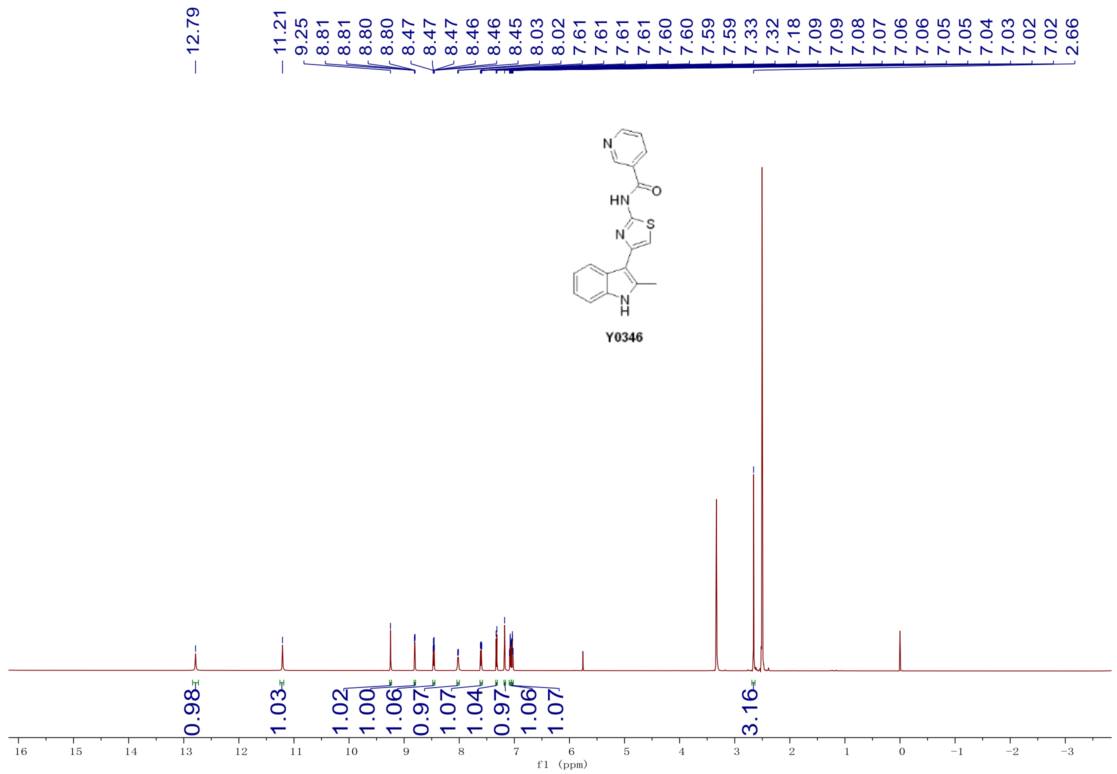

Compound **Y0346**, ^{13}C NMR (126 MHz, DMSO-d_6)

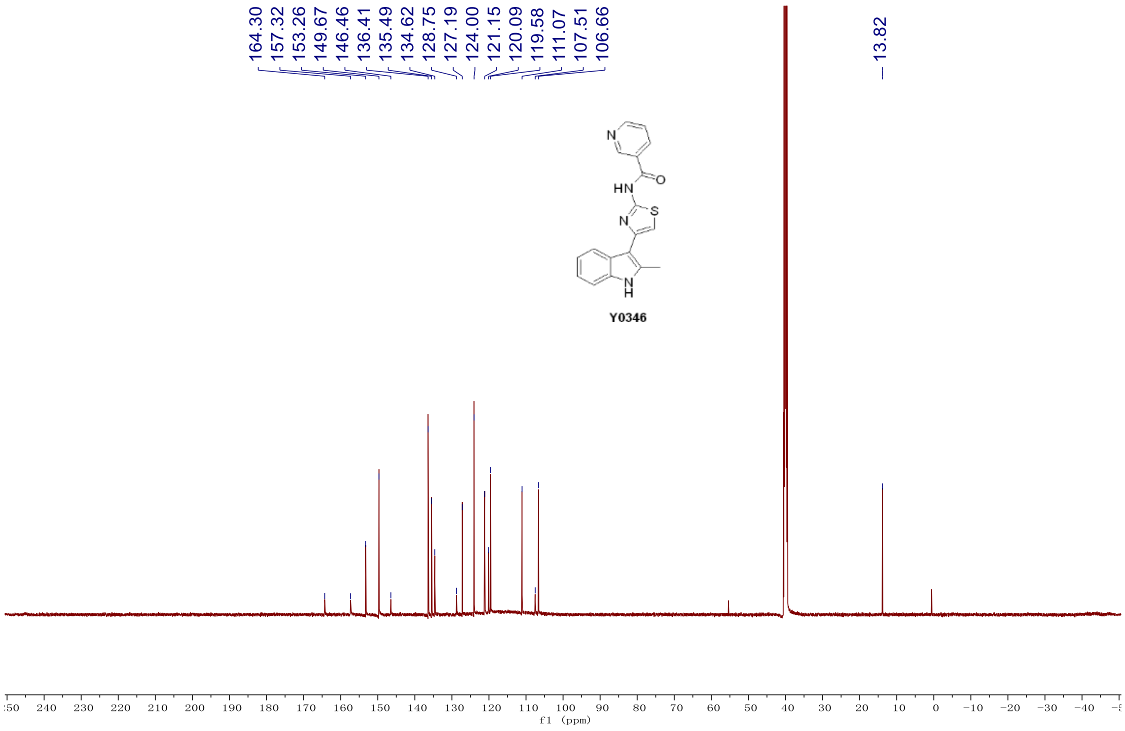

Compound **Y0346**, HRMS (ESI)

Mass Spectrum SmartFormula Report

Analysis Info

Analysis Name	D:\Data\SHUJVFENXI\DINGKE-GROUP\2021187-yjh-03-46-P_BC6_01_42552.d	Acquisition Date	3/12/2024 11:26:45 AM
Method	6min.m		
Sample Name	2021187-yjh-03-46-P	Operator	BDAL@DE
Comment		Instrument / Ser#	maXis 4G 21240

Acquisition Parameter

Source Type	ESI	Ion Polarity	Positive	Set Nebulizer	1.0 Bar
Focus	Not active	Set Capillary	3000 V	Set Dry Heater	220 °C
Scan Begin	50 m/z	Set End Plate Offset	-500 V	Set Dry Gas	6.0 l/min
Scan End	1500 m/z	Set Collision Cell RF	600.0 Vpp	Set Divert Valve	Waste

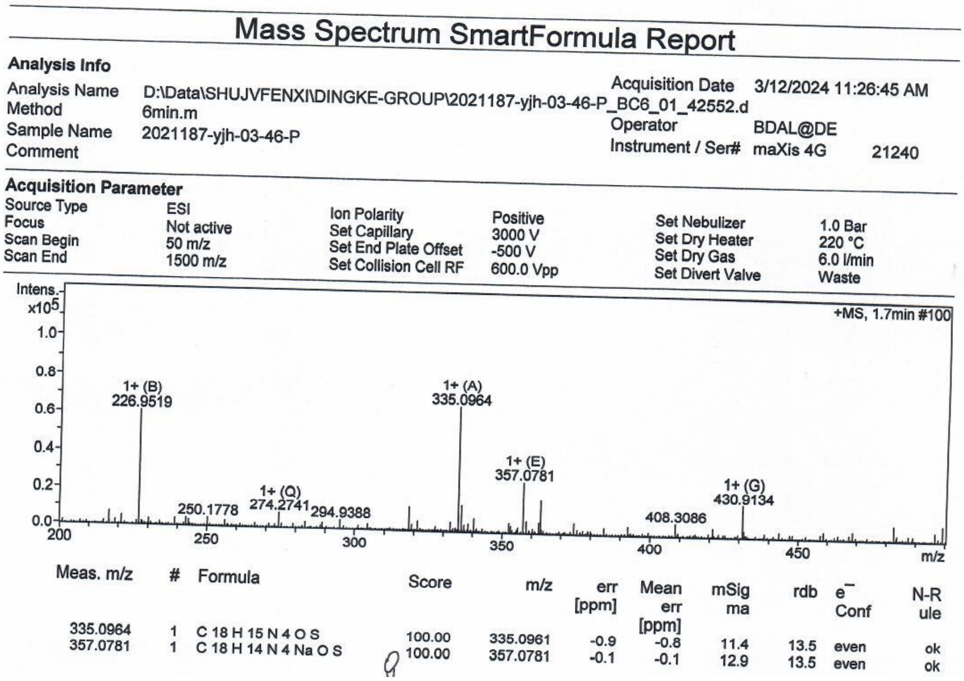

Meas. m/z	#	Formula	Score	m/z	err [ppm]	Mean err [ppm]	mSigma	rdb	e⁻ Conf	N-Rule
335.0964	1	C 18 H 15 N 4 O S	100.00	335.0961	-0.9	-0.8	11.4	13.5	even	ok
357.0781	1	C 18 H 14 N 4 Na O S	100.00	357.0781	-0.1	-0.1	12.9	13.5	even	ok

Compound **Y0346**, HPLC

Single Injection Report

Agilent

**Data file:**	YJH-03-46-P.dx		
**Sequence Name:**	YJH-03-46-P	**Project Name:**	1260
**Sample name:**	YJH-03-46-P	**Operator:**	SYSTEM
**Instrument:**	1260	**Injection date:**	2023-02-20 12:38:05+08:00
**Inj. volume:**	10.000 µL	**Location:**	P1-D1
**Acq. method:**	normal.amx	**Type:**	Sample
**Processing method:**	GC_LC Area Percent_DefaultMethod.pmx	**Sample amount:**	0.00
**Manually modified:**	Manual Integration		

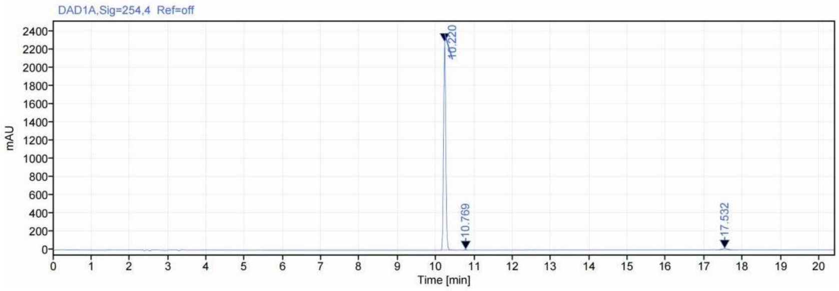

Signal: DAD1A,Sig=254,4 Ref=off

RT [min]	Type	Width [min]	Area	Height	Area%	Name
10.220	BV	0.50	9049.43	2282.25	97.63	
10.769	VV	0.20	25.57	5.75	0.28	
17.532	VV	0.41	194.07	15.79	2.09	
		Sum	9269.07		100.00	

Compound **Y0344**, ^{1}H NMR (600 MHz, DMSO-d_6)

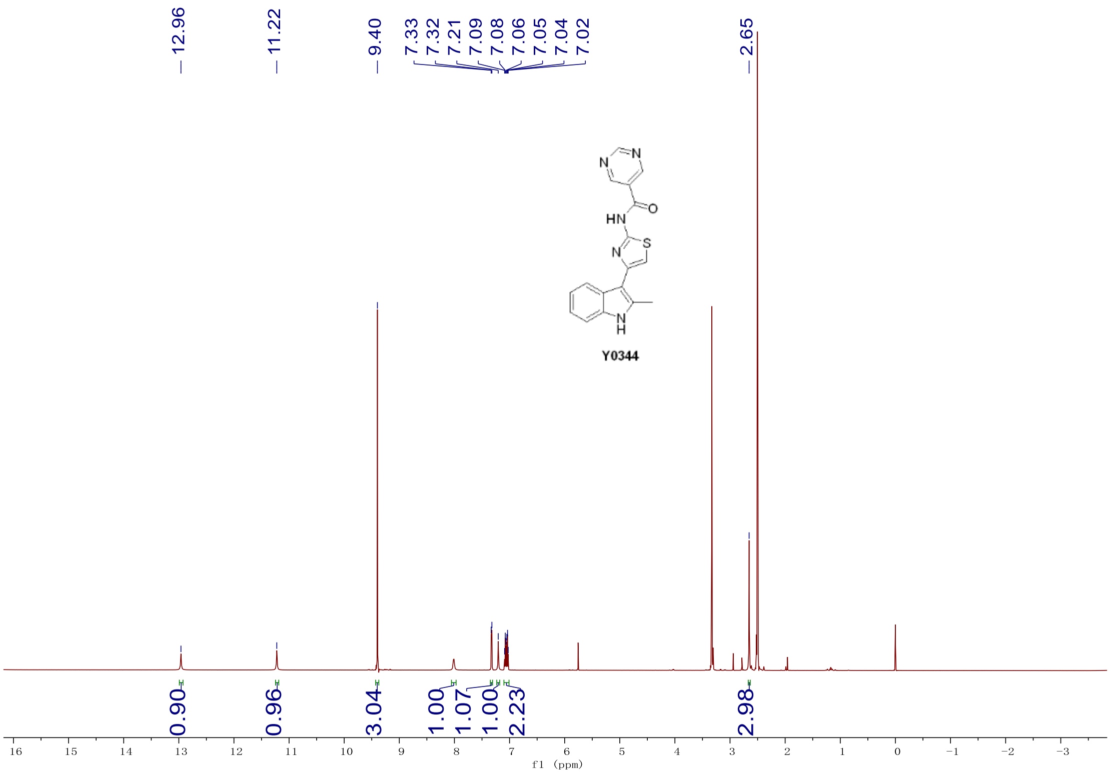

Compound **Y0344**, ^{13}C NMR (126 MHz, DMSO-d_6)

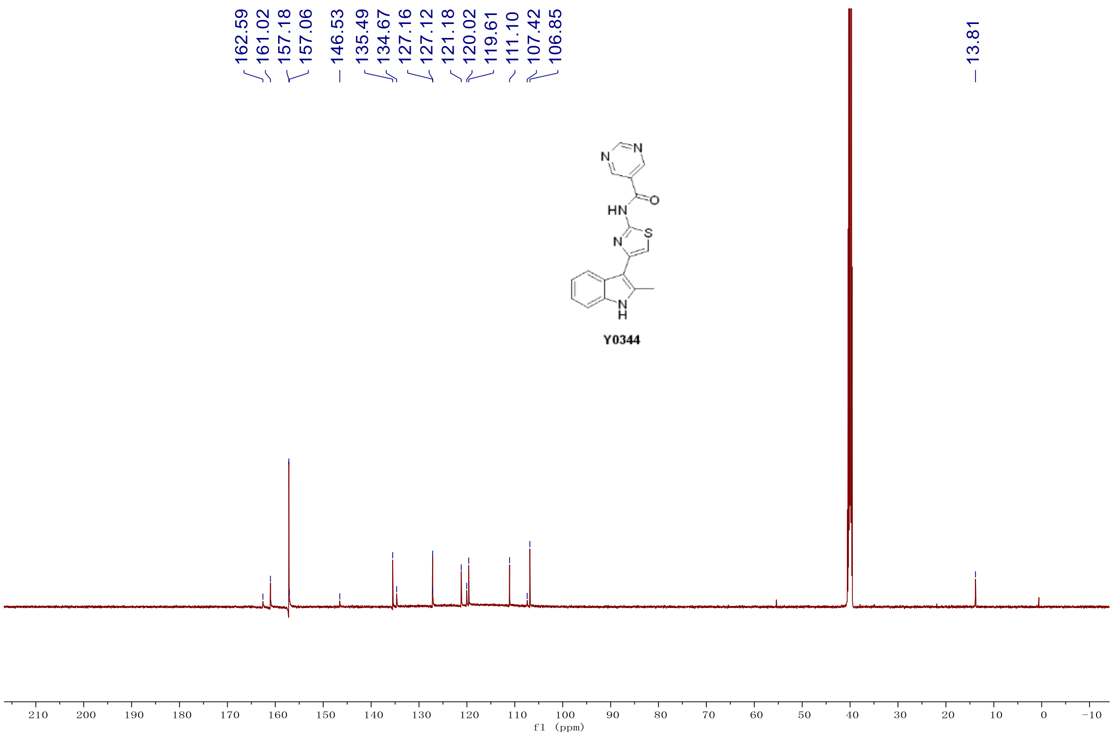

Compound **Y0344**, HRMS (ESI)

Mass Spectrum SmartFormula Report

Analysis Info

Analysis Name	D:\Data\SHUJVFENXI\DINGKE-GROUP\2021187-yjh-0344-P_BC5_01_42550.d	Acquisition Date	3/12/2024 11:12:04 AM
Method	6min.m		
Sample Name	2021187-yjh-0344-P	Operator	BDAL@DE
Comment		Instrument / Ser#	maXis 4G 21240

Acquisition Parameter

Source Type	ESI	Ion Polarity	Positive	Set Nebulizer	1.0 Bar
Focus	Not active	Set Capillary	3000 V	Set Dry Heater	220 °C
Scan Begin	50 m/z	Set End Plate Offset	-500 V	Set Dry Gas	6.0 l/min
Scan End	1500 m/z	Set Collision Cell RF	600.0 Vpp	Set Divert Valve	Waste

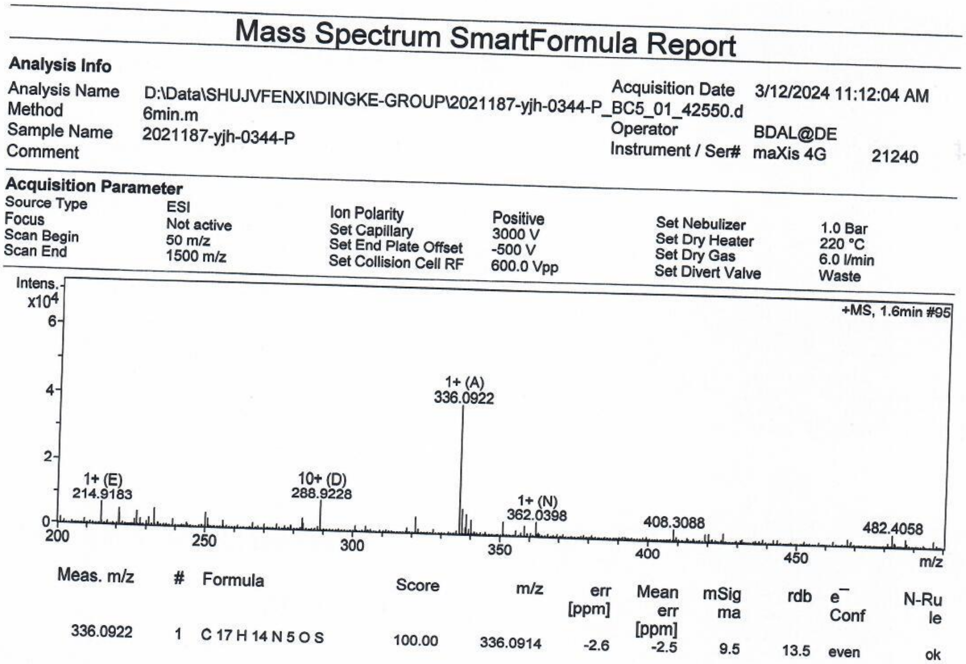

Meas. m/z	#	Formula	Score	m/z	err [ppm]	Mean err [ppm]	mSigma	rdb	e⁻ Conf	N-Rule
336.0922	1	C 17 H 14 N 5 O S	100.00	336.0914	-2.6	-2.5	9.5	13.5	even	ok

Compound **Y0344**, HPLC

Single Injection Report

Agilent

**Data file:**	YJH-03-44-P.dx		
**Sequence Name:**	YJH-03-44-P	**Project Name:**	1260
**Sample name:**	YJH-03-44-P	**Operator:**	SYSTEM
**Instrument:**	1260	**Injection date:**	2023-02-19 19:19:02+08:00
**Inj. volume:**	10.000 µL	**Location:**	P1-D2
**Acq. method:**	normal.amx	**Type:**	Sample
**Processing method:**	GC_LC Area Percent_DefaultMethod.pmx	**Sample amount:**	0.00
**Manually modified:**	Manual Integration		

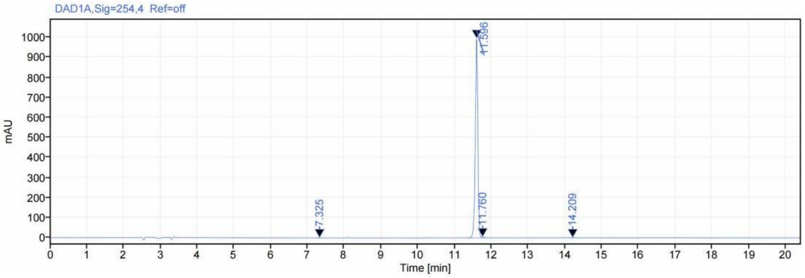

Signal: DAD1A,Sig=254,4 Ref=off

RT [min]	Type	Width [min]	Area	Height	Area%	Name
7.325	BB	0.28	8.35	2.04	0.17	
11.596	BV	0.47	4778.57	997.98	99.11	
11.760	VB	0.23	26.91	6.11	0.56	
14.209	BB	0.20	7.55	1.81	0.16	
		**Sum**	4821.38		100.00	

Compound **Y0747**, ^{1}H NMR (600 MHz, DMSO-d_6)

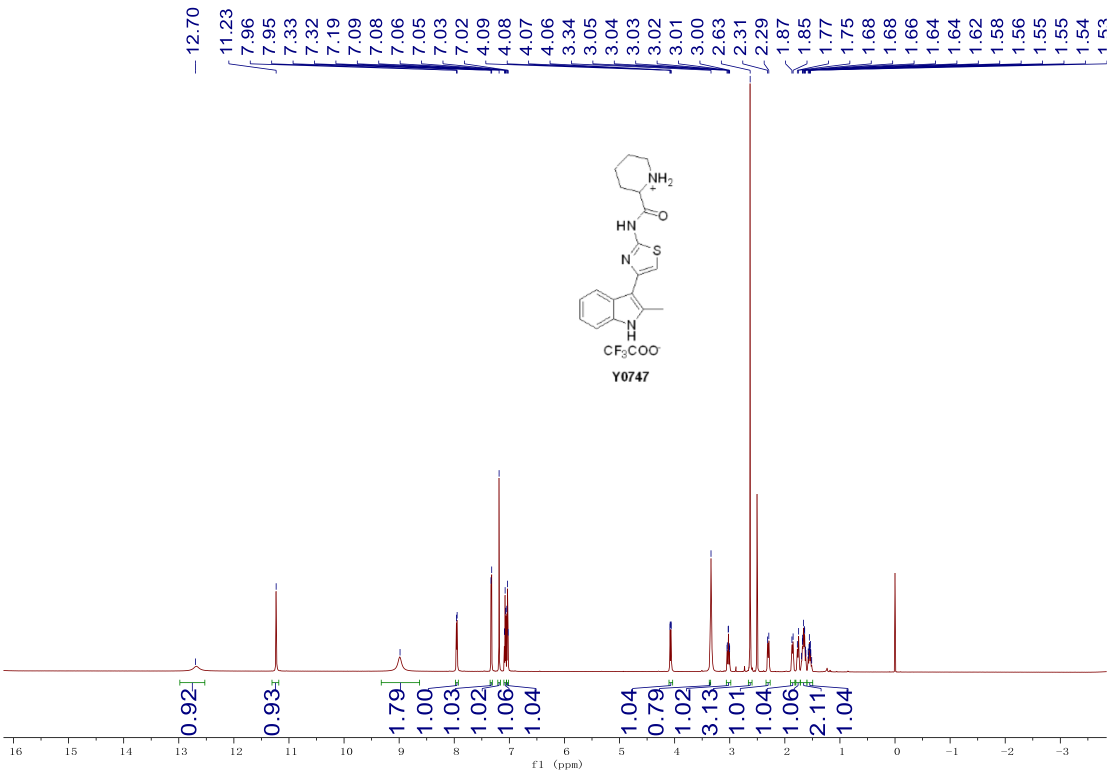

Compound **Y0747**, ^{13}C NMR (126 MHz, DMSO-d_6)

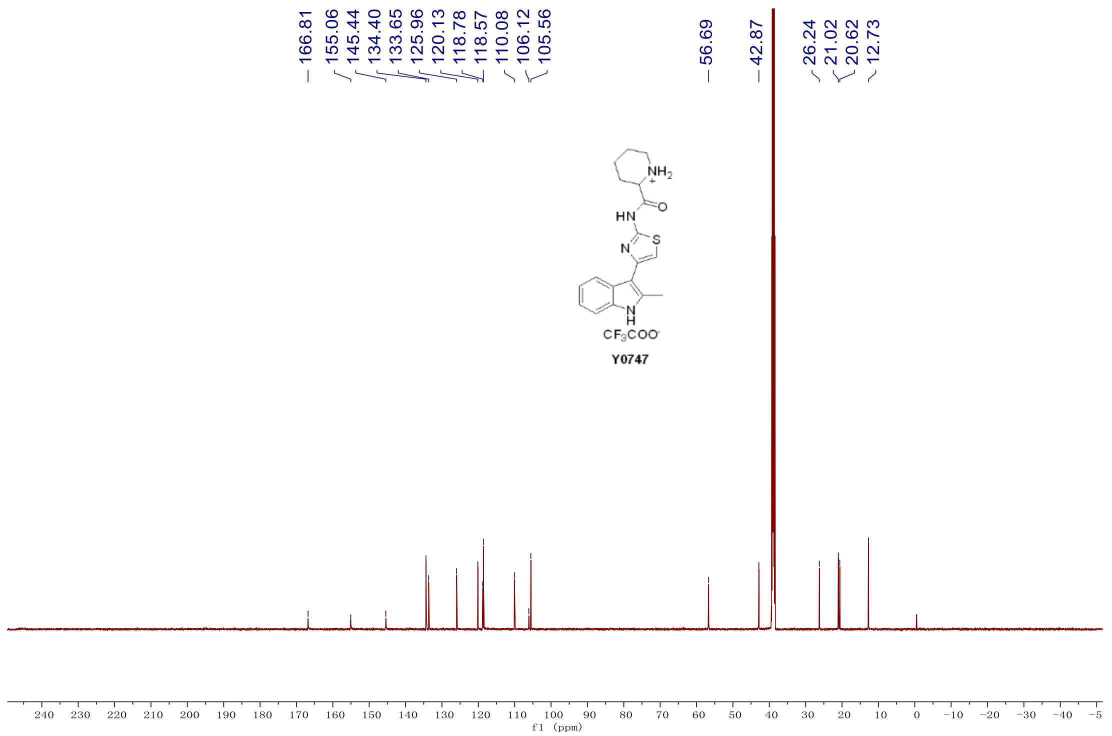

Compound **Y0747**, ^{19}F NMR (376 MHz, DMSO-d_6)

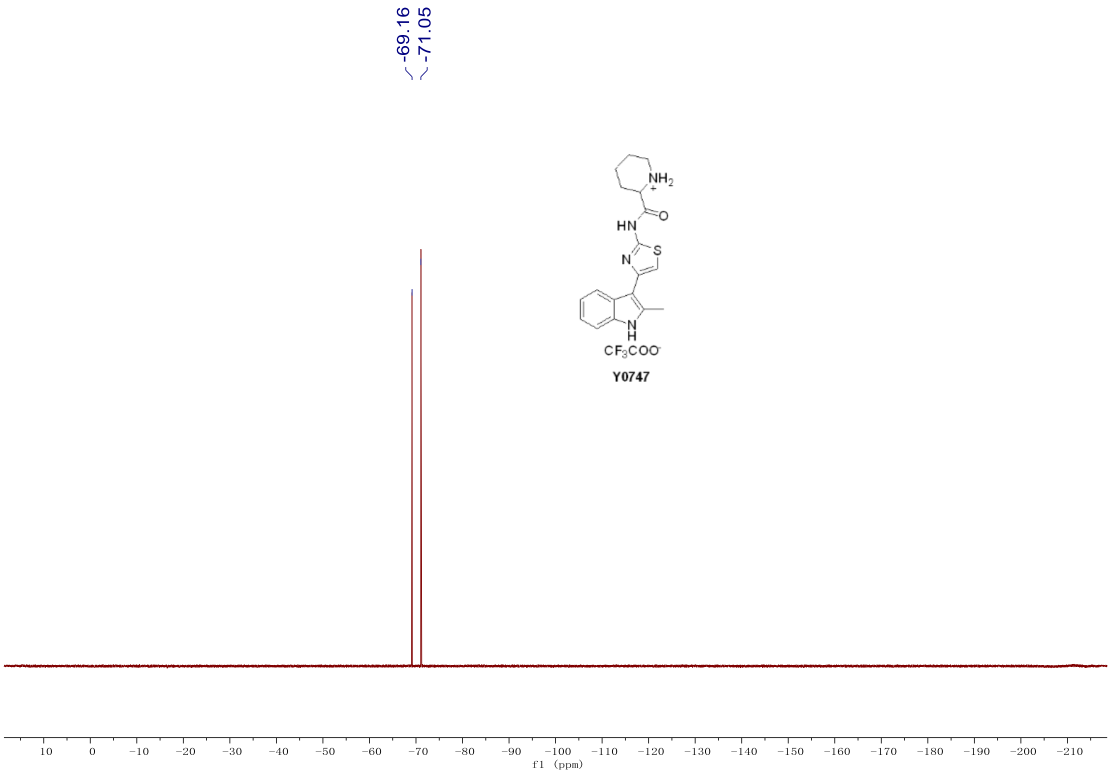

Compound **Y0747**, HRMS (ESI)

Mass Spectrum SmartFormula Report

Analysis Info

Analysis Name	D:\Data\SHUJVFENXI\DINGKE-GROUP\2021187-yjh-07-47-P_GB8_01_40653.d	Acquisition Date	1/5/2024 12:52:50 PM
Method	6min.m	Operator	BDAL@DE
Sample Name	2021187-yjh-07-47-P	Instrument / Ser#	maXis 4G 21240
Comment			

Acquisition Parameter

Source Type	ESI	Ion Polarity	Positive	Set Nebulizer	1.0 Bar
Focus	Not active	Set Capillary	3000 V	Set Dry Heater	220 °C
Scan Begin	50 m/z	Set End Plate Offset	-500 V	Set Dry Gas	6.0 l/min
Scan End	1500 m/z	Set Collision Cell RF	600.0 Vpp	Set Divert Valve	Waste

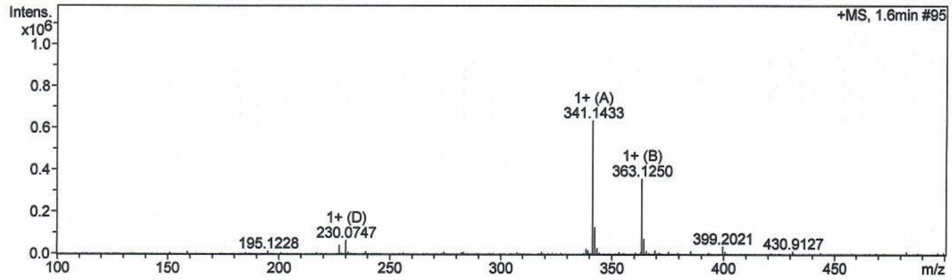

Meas. m/z	#	Formula	Score	m/z	err [ppm]	Mean err [ppm]	mSigma	rdb	e¯ Conf	N-Rule
341.1433	1	C 18 H 21 N 4 O S	100.00	341.1431	-0.8	-0.7	14.3	10.5	even	ok
363.1250	1	C 18 H 20 N 4 Na O S	100.00	363.1250	0.0	0.0	14.6	10.5	even	ok

Compound **Y0747**, HPLC

Single Injection Report

Agilent

**Data file:**	YJH-07-47-P.dx		
**Sequence Name:**	SingleSample	**Project Name:**	1260
**Sample name:**	YJH-07-47-P	**Operator:**	SYSTEM
**Instrument:**	1260	**Injection date:**	2023-12-27 13:15:04+08:00
**Inj. volume:**	20.000 µL	**Location:**	P1-C1
**Acq. method:**	normal.amx	**Type:**	Sample
**Processing method:**	INM.pmx	**Sample amount:**	0.00
**Manually modified:**	Manual Integration		

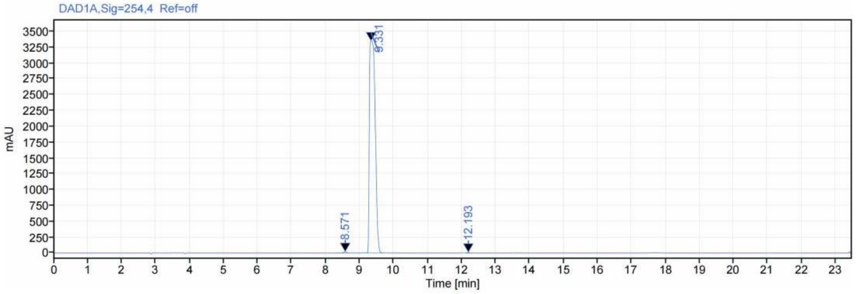

Signal: DAD1A,Sig=254,4 Ref=off

RT [min]	Type	Width [min]	Area	Height	Area%	Name
8.571	VV	0.41	140.90	19.63	0.35	
9.331	VV	0.47	39971.48	3347.29	99.50	
12.193	VB	0.25	60.76	10.83	0.15	
		**Sum**	40173.14		100.00	

Compound **Y0739**, ^{1}H NMR (600 MHz, DMSO-d_6)

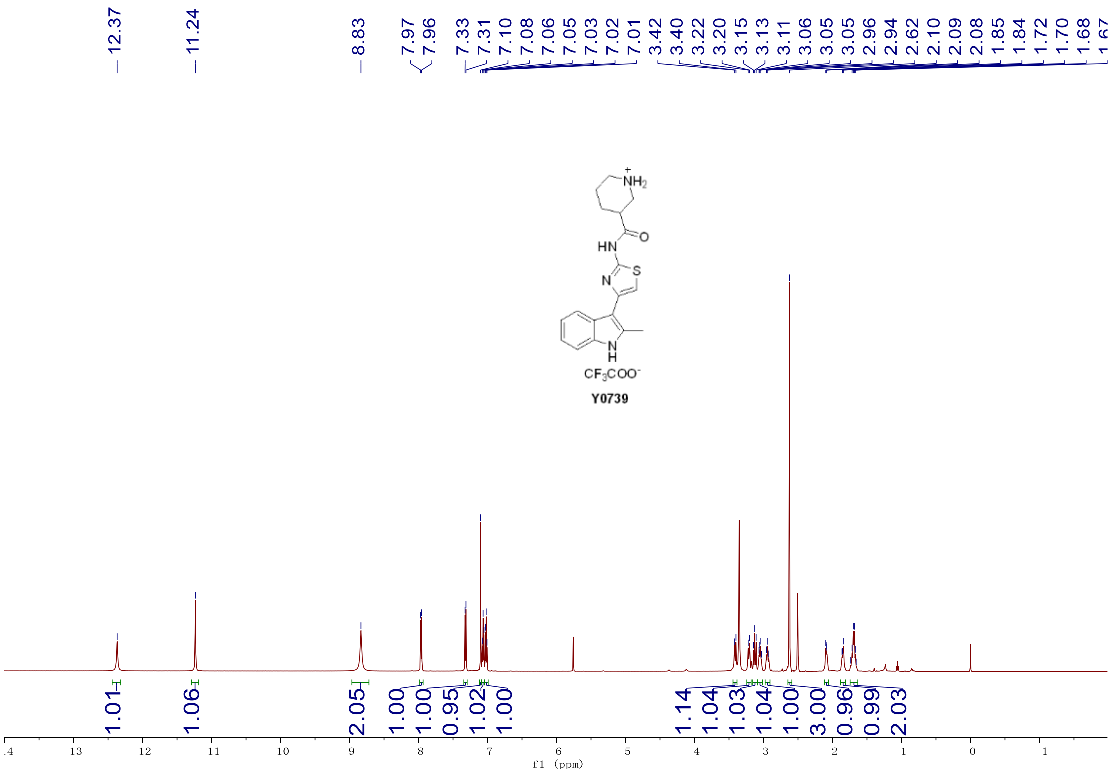

Compound **Y0739**, ^{13}C NMR (126 MHz, DMSO-d_6)

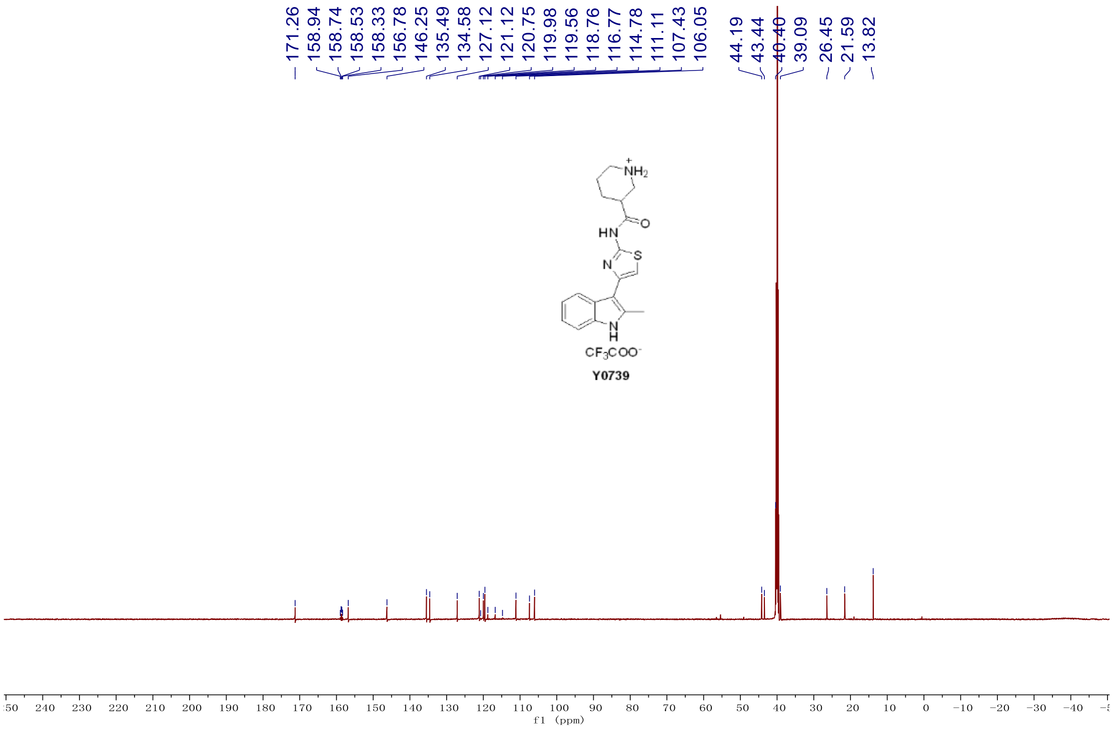

Compound **Y0739**, ^{19}F NMR (376 MHz, DMSO-d_6)

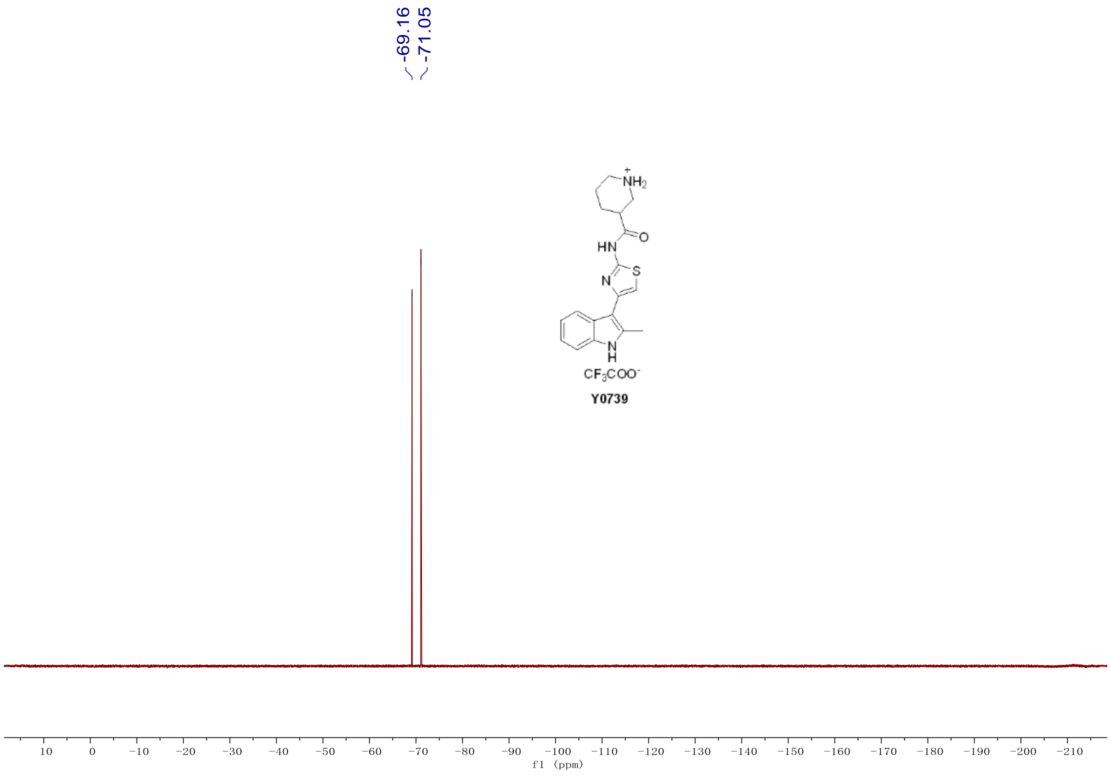

Compound **Y0739**, HRMS (ESI)

Mass Spectrum SmartFormula Report

Analysis Info

Analysis Name	D:\Data\SHUJVFENXI\DINGKE-GROUP\2021187-yjh-07-39-P_GB5_01_40647.d	Acquisition Date	1/5/2024 12:14:16 PM
Method	6min.m		
Sample Name	2021187-yjh-07-39-P	Operator	BDAL@DE
Comment		Instrument / Ser#	maXis 4G 21240

Acquisition Parameter

Source Type	ESI	Ion Polarity	Positive	Set Nebulizer	1.0 Bar
Focus	Not active	Set Capillary	3000 V	Set Dry Heater	220 °C
Scan Begin	50 m/z	Set End Plate Offset	-500 V	Set Dry Gas	6.0 l/min
Scan End	1500 m/z	Set Collision Cell RF	600.0 Vpp	Set Divert Valve	Waste

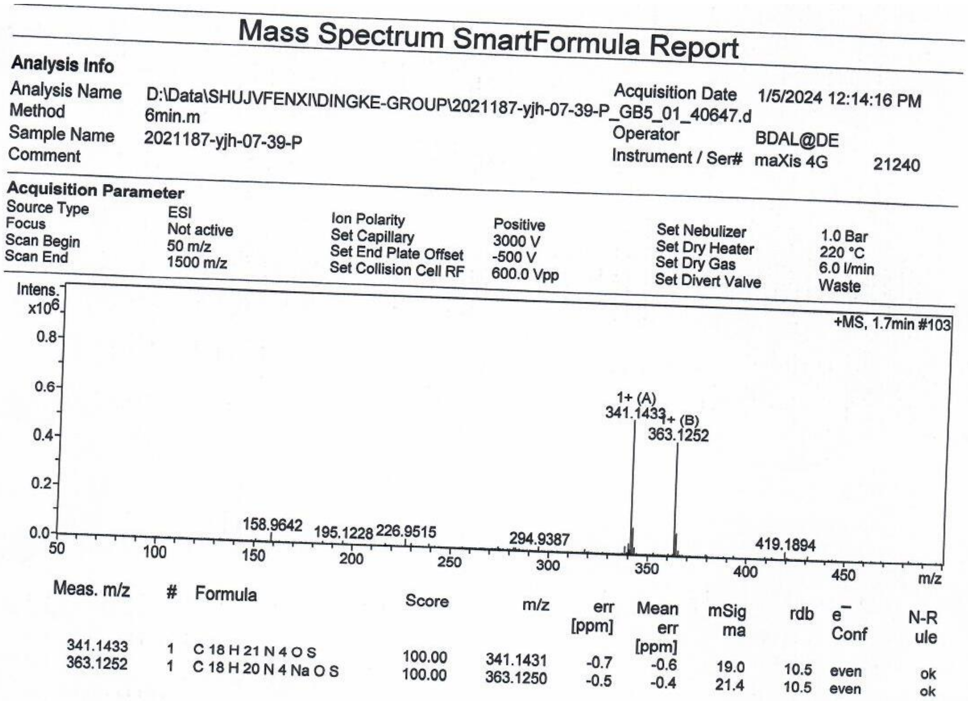

Meas. m/z	#	Formula	Score	m/z	err [ppm]	Mean err [ppm]	mSigma	rdb	e⁻ Conf	N-Rule
341.1433	1	C 18 H 21 N 4 O S	100.00	341.1431	-0.7	-0.6	19.0	10.5	even	ok
363.1252	1	C 18 H 20 N 4 Na O S	100.00	363.1250	-0.5	-0.4	21.4	10.5	even	ok

Compound **Y0739**, HPLC

Single Injection Report

Agilent

**Data file:**	YJH-07-39-P.dx		
**Sequence Name:**	SingleSample	**Project Name:**	1260
**Sample name:**	YJH-07-39-P	**Operator:**	SYSTEM
**Instrument:**	1260	**Injection date:**	2023-12-24 16:33:16+07:00
**Inj. volume:**	15.000 µL	**Location:**	P1-C1
**Acq. method:**	normal.amx	**Type:**	Sample
**Processing method:**	*INM.pmx	**Sample amount:**	0.00
**Manually modified:**	Manual Integration		

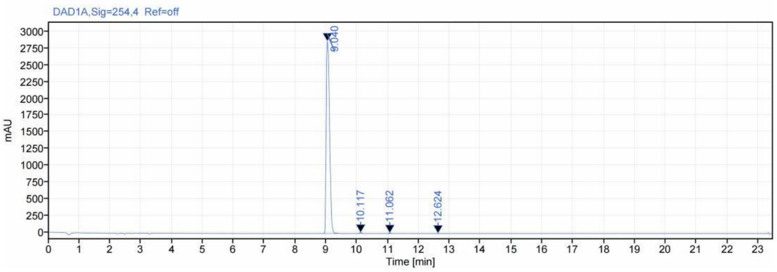

Signal: DAD1A,Sig=254,4 Ref=off

RT [min]	Type	Width [min]	Area	Height	Area%	Name
9.040	VB	0.84	21790.55	2866.18	99.51	
10.117	MM m	0.16	55.71	12.48	0.25	
11.062	MM m	0.18	34.76	7.55	0.16	
12.624	BV	0.46	15.95	2.07	0.07	
		**Sum**	21896.97		100.00	

Compound **PBITE-1**, ^{1}H NMR (600 MHz, DMSO-d_6)

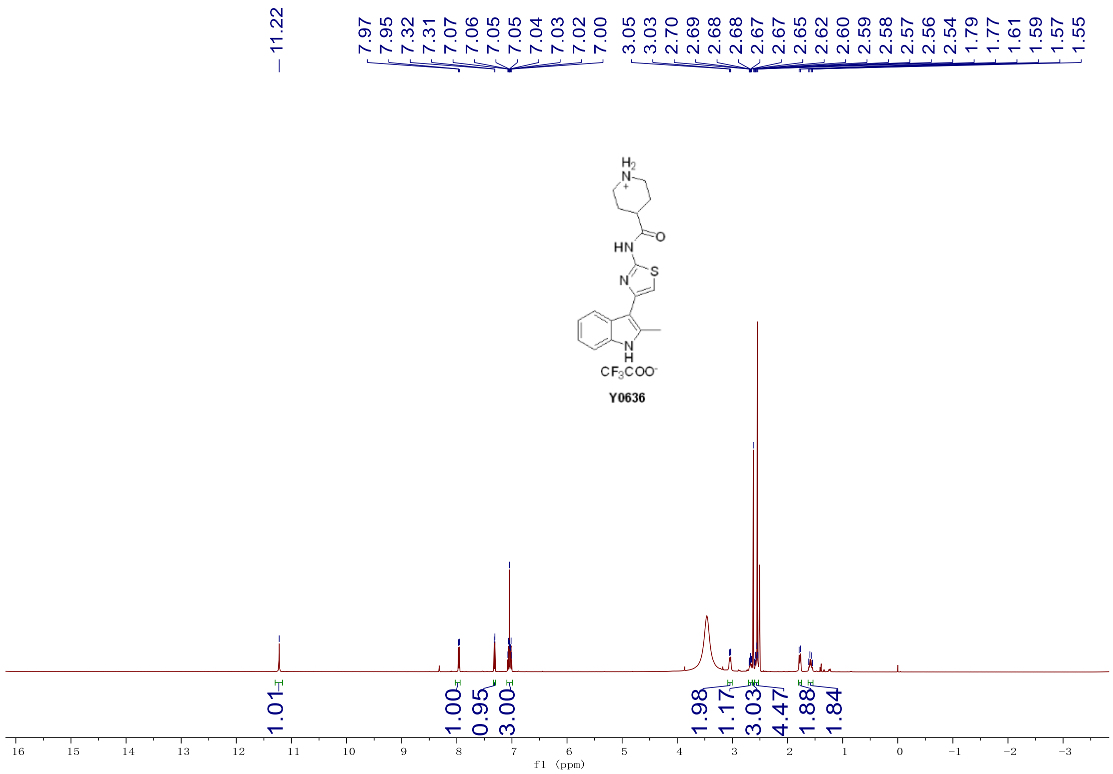

Compound **PBITE-1**, ^{13}C NMR (126 MHz, DMSO-d_6)

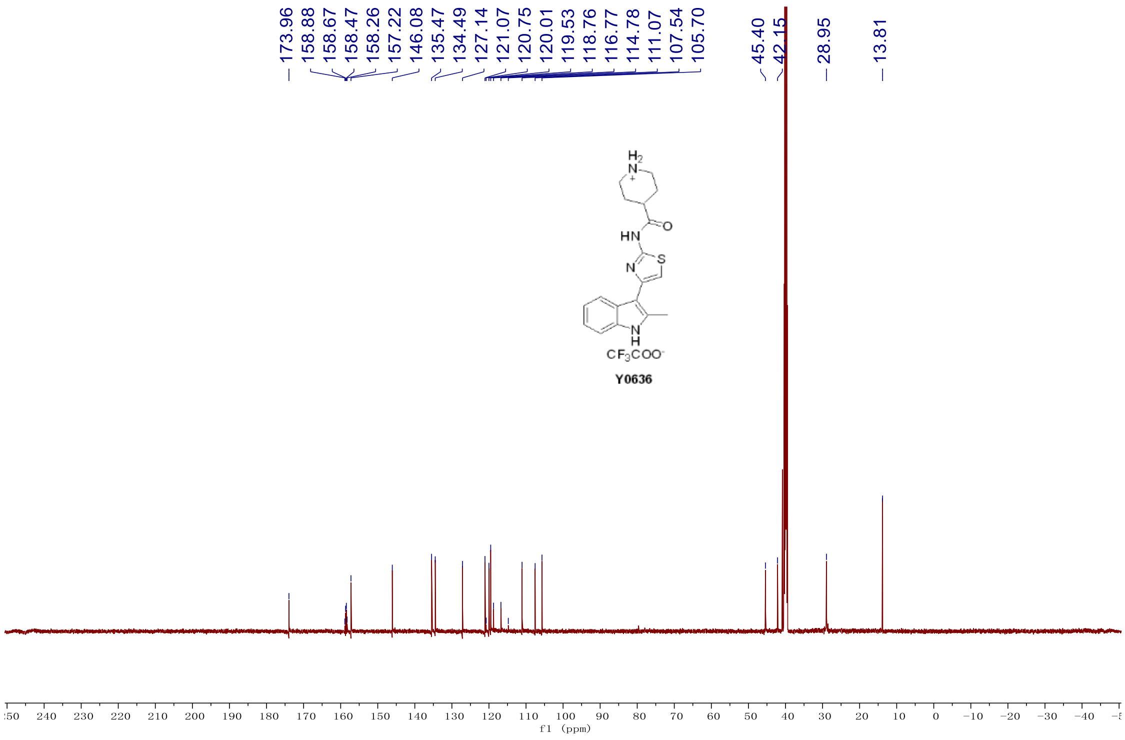

Compound **PBITE-1**, ^{19}F NMR (376 MHz, DMSO-d_6)

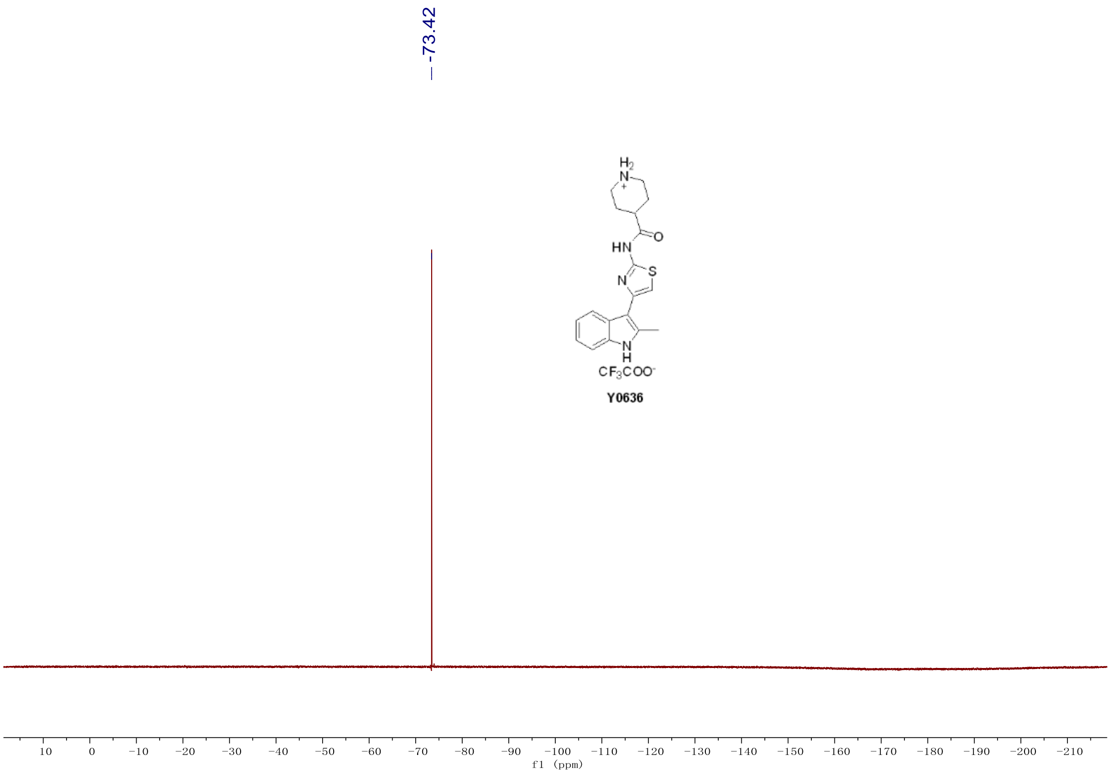

Compound **PBITE-1**, HRMS (ESI)

Mass Spectrum SmartFormula Report

Analysis Info

Analysis Name	D:\Data\SHUJVFENXI\DINGKE-GROUP\2021187-yjh-06-36-P_RE8_01_38636.d	Acquisition Date	10/18/2023 4:58:07 PM
Method	20150915.m	Operator	BDAL@DE
Sample Name	2021187-yjh-06-36-P	Instrument / Ser#	maXis 4G 21240
Comment			

Acquisition Parameter

Source Type	ESI	Ion Polarity	Positive	Set Nebulizer	1.0 Bar
Focus	Not active	Set Capillary	3000 V	Set Dry Heater	220 °C
Scan Begin	50 m/z	Set End Plate Offset	-500 V	Set Dry Gas	6.0 l/min
Scan End	1500 m/z	Set Collision Cell RF	600.0 Vpp	Set Divert Valve	Waste

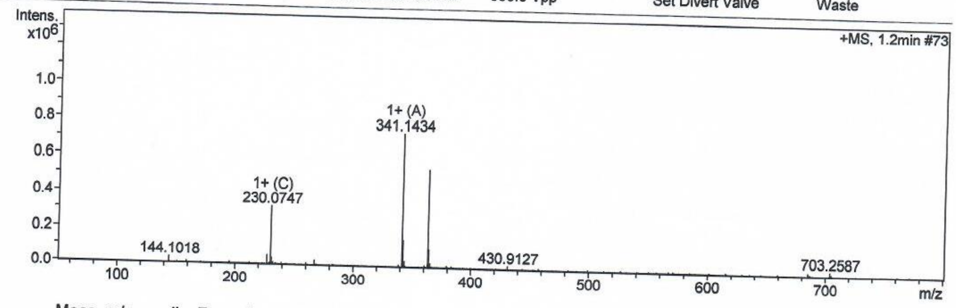

Meas. m/z	#	Formula	Score	m/z	err [ppm]	Mean err [ppm]	mSigma	rdb	e⁻ Conf	N-Rule
341.1434	1	C 18 H 21 N 4 O S	100.00	341.1431	-1.0	-0.8	13.5	10.5	even	ok
363.1251	1	C 18 H 20 N 4 Na O S	100.00	363.1250	-0.2	0.0	21.8	10.5	even	ok

Compound **PBITE-1**, HPLC

Single Injection Report

Agilent

**Data file:**	YJH-06-36-P.dx		
**Sequence Name:**	SingleSample	**Project Name:**	1260
**Sample name:**	YJH-06-36-P	**Operator:**	SYSTEM
**Instrument:**	1260	**Injection date:**	2023-09-03 20:41:18+08:00
**Inj. volume:**	15.000 µL	**Location:**	P1-C1
**Acq. method:**	normal.amx	**Type:**	Sample
**Processing method:**	INM.pmx	**Sample amount:**	0.00
**Manually modified:**	Manual Integration		

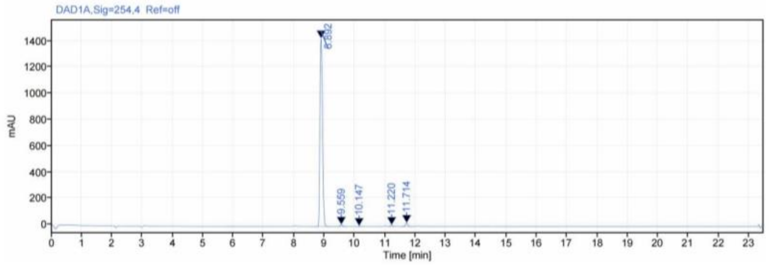

Signal: DAD1A,Sig=254,4 Ref=off

RT [min]	Type	Width [min]	Area	Height	Area%	Name
8.892	VB	0.54	8362.20	1434.63	96.40	
9.559	BB	0.59	84.08	12.92	0.97	
10.147	BB	0.33	18.37	3.43	0.21	
11.220	VV	0.22	62.99	10.71	0.73	
11.714	BV	0.39	146.94	26.58	1.69	
		**Sum**	8674.58		100.00	

Compound **Y0640**, ^{1}H NMR (600 MHz, DMSO-d_6)

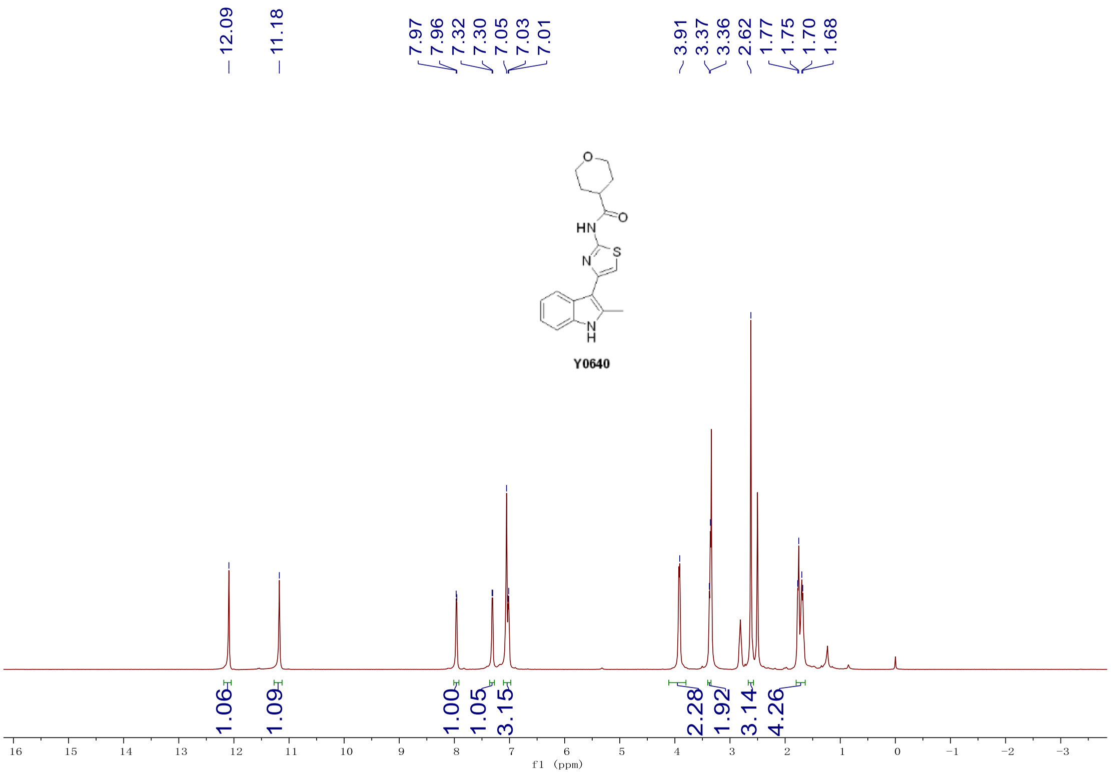

Compound **Y0640**, ^{13}C NMR (126 MHz, DMSO-d_6)

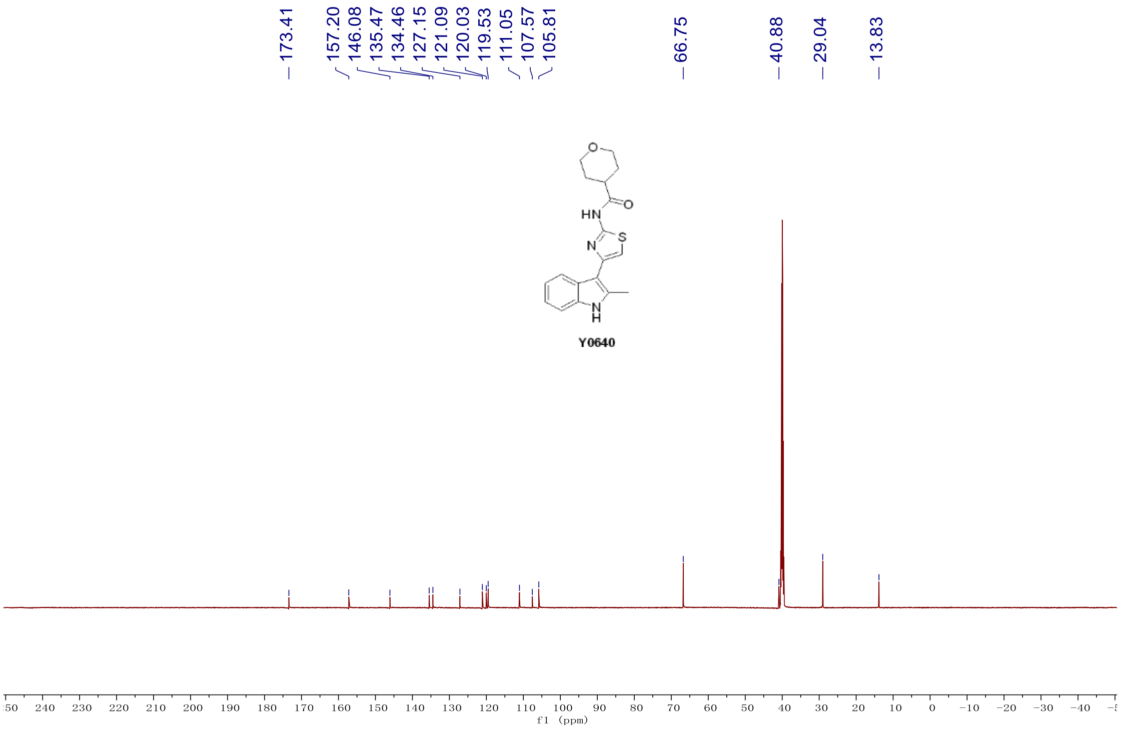

Compound **Y0640**, HRMS (ESI)

Mass Spectrum SmartFormula Report

Analysis Info

Analysis Name	D:\Data\SHUJVFENXI\DINGKE-GROUP\2021187-YJH-06-40-P_BB3_01_38745.d	Acquisition Date	10/23/2023 1:31:04 PM
Method	20150915.m	Operator	BDAL@DE
Sample Name	2021187-YJH-06-40-P	Instrument / Ser#	maXis 4G 21240
Comment			

Acquisition Parameter

Source Type	ESI	Ion Polarity	Positive	Set Nebulizer	1.0 Bar
Focus	Not active	Set Capillary	3000 V	Set Dry Heater	220 °C
Scan Begin	50 m/z	Set End Plate Offset	-500 V	Set Dry Gas	6.0 l/min
Scan End	1500 m/z	Set Collision Cell RF	600.0 Vpp	Set Divert Valve	Waste

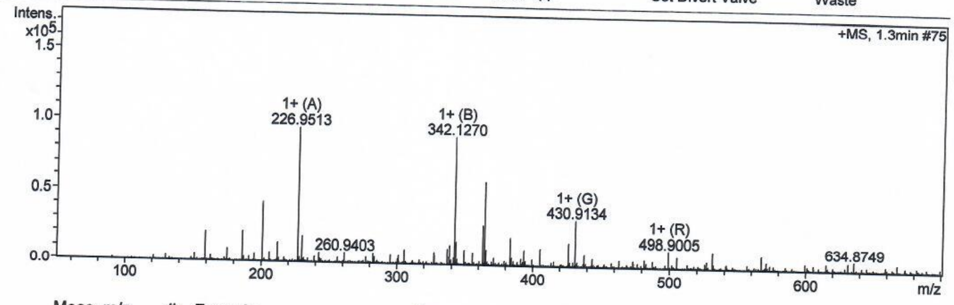

Meas. m/z	#	Formula	Score	m/z	err [ppm]	Mean err [ppm]	mSigma	rdb	e⁻ Conf	N-Rule
342.1270	1	C 18 H 20 N 3 O 2 S	100.00	342.1271	0.2	0.0	18.3	10.5	even	ok
364.1089	1	C 18 H 19 N 3 Na O 2 S	100.00	364.1090	0.2	-0.1	14.2	10.5	even	ok

Compound **Y0640**, HPLC

Single Injection Report

Agilent

**Data file:**	YJH-06-40.dx		
**Sequence Name:**	SingleSample	**Project Name:**	1260
**Sample name:**	YJH-06-40-P	**Operator:**	SYSTEM
**Instrument:**	1260	**Injection date:**	2023-09-05 21:51:13+08:00
**Inj. volume:**	15.000 µL	**Location:**	P1-B1
**Acq. method:**	normal.amx	**Type:**	Sample
**Processing method:**	INM.pmx	**Sample amount:**	0.00
**Manually modified:**	Manual Integration		

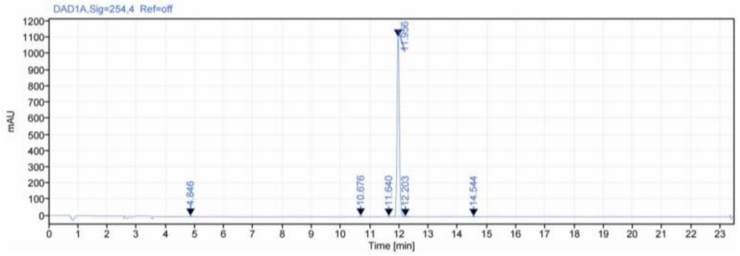

Signal: DAD1A,Sig=254,4 Ref=off

RT [min]	Type	Width [min]	Area	Height	Area%	Name
4.846	BB	0.47	27.77	4.84	0.44	
10.676	VV	0.21	37.47	6.30	0.59	
11.640	VB	0.24	23.99	3.96	0.38	
11.956	BV	0.36	6213.46	1111.85	97.90	
12.203	VV	0.20	19.81	2.26	0.31	
14.544	VV	0.31	24.55	2.92	0.39	
		**Sum**	6347.05		100.00	

Compound **Y0743**, ^{1}H NMR (600 MHz, DMSO-d_6)

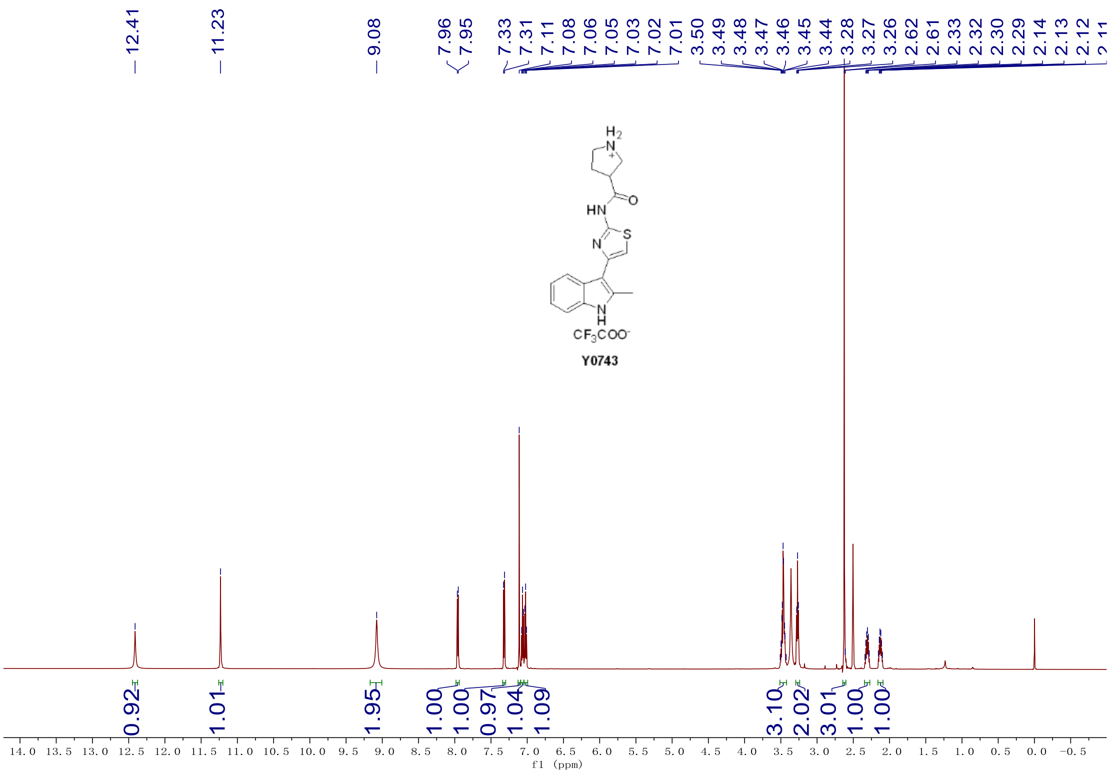

Compound **Y0743**, ^{13}C NMR (126 MHz, DMSO-d_6)

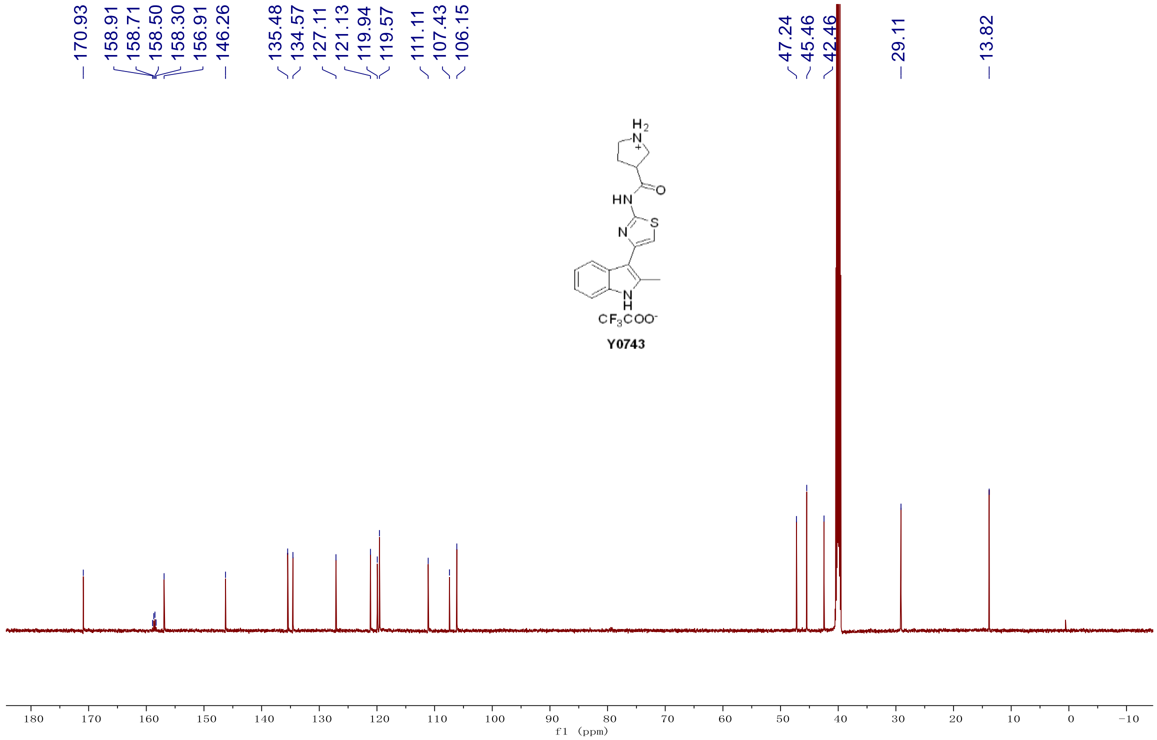

Compound **Y0743**, ^{19}F NMR (376 MHz, DMSO-d_6)

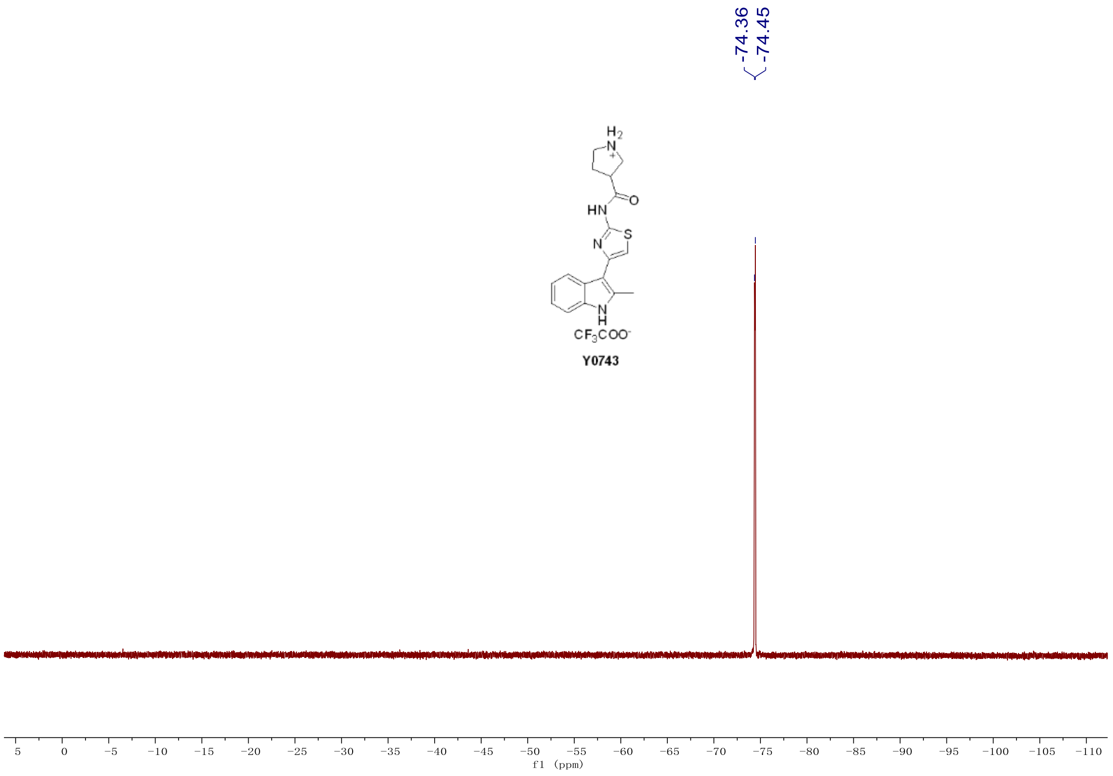

Compound **Y0743**, HRMS (ESI)

Mass Spectrum SmartFormula Report

Analysis Info

Analysis Name	D:\Data\SHUJVFENXI\DINGKE-GROUP\2021187-yjh-07-43-P_GB6_01_40648.d	Acquisition Date	1/5/2024 12:21:36 PM
Method	6min.m	Operator	BDAL@DE
Sample Name	2021187-yjh-07-43-P	Instrument / Ser#	maXis 4G 21240
Comment			

Acquisition Parameter

Source Type	ESI	Ion Polarity	Positive	Set Nebulizer	1.0 Bar
Focus	Not active	Set Capillary	3000 V	Set Dry Heater	220 °C
Scan Begin	50 m/z	Set End Plate Offset	-500 V	Set Dry Gas	6.0 l/min
Scan End	1500 m/z	Set Collision Cell RF	600.0 Vpp	Set Divert Valve	Waste

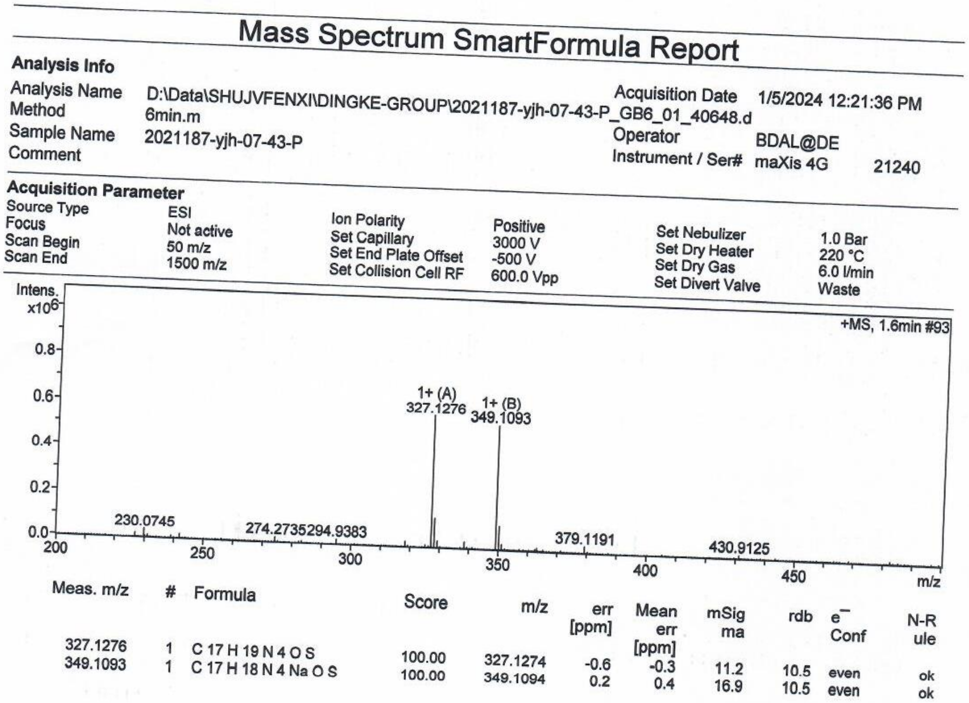

Meas. m/z	#	Formula	Score	m/z	err [ppm]	Mean err [ppm]	mSigma	rdb	e⁻ Conf	N-Rule
327.1276	1	C 17 H 19 N 4 O S	100.00	327.1274	-0.6	-0.3	11.2	10.5	even	ok
349.1093	1	C 17 H 18 N 4 Na O S	100.00	349.1094	0.2	0.4	16.9	10.5	even	ok

Compound **Y0743**, HPLC

Single Injection Report

Agilent

**Data file:**	YJH-07-43-P.dx		
**Sequence Name:**	SingleSample	**Project Name:**	1260
**Sample name:**	YJH-07-43-P	**Operator:**	SYSTEM
**Instrument:**	1260	**Injection date:**	2023-12-24 15:30:40+08:00
**Inj. volume:**	15.000 µL	**Location:**	P1-C1
**Acq. method:**	normal.amx	**Type:**	Sample
**Processing method:**	INM.pmx	**Sample amount:**	0.00
**Manually modified:**	Manual Integration		

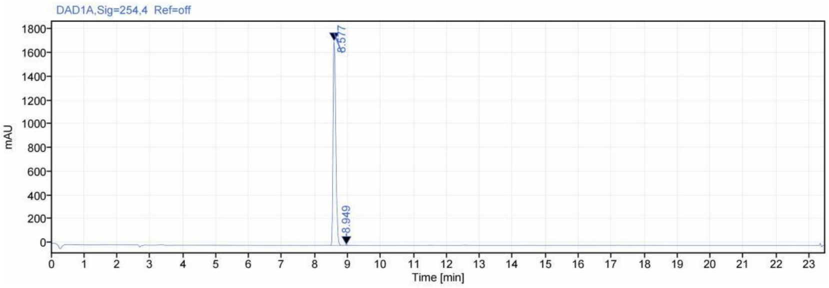

Signal: DAD1A,Sig=254,4 Ref=off

RT [min]	Type	Width [min]	Area	Height	Area%	Name
8.577	VV	0.42	9988.58	1722.38	99.82	
8.949	VB	0.40	18.39	3.56	0.18	
		**Sum**	10006.97		100.00	

Compound **Y0752**, ^{1}H NMR (600 MHz, DMSO-d_6)

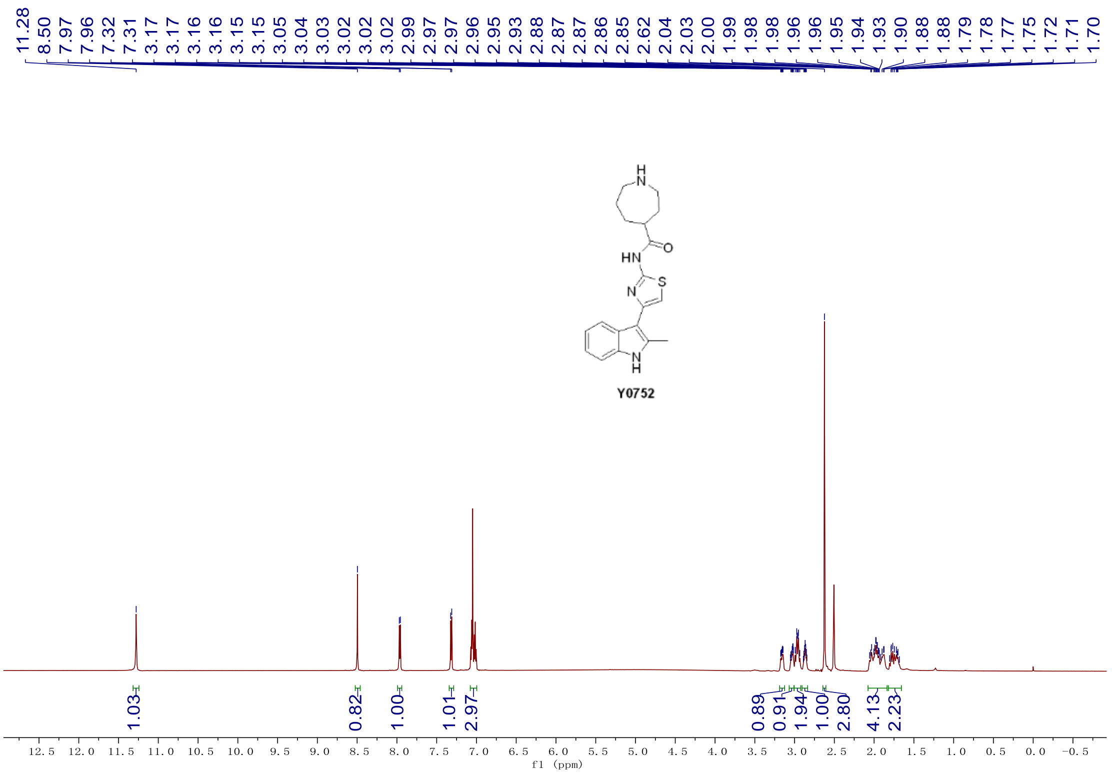

Compound **Y0752**, ^{13}C NMR (126 MHz, DMSO-d_6)

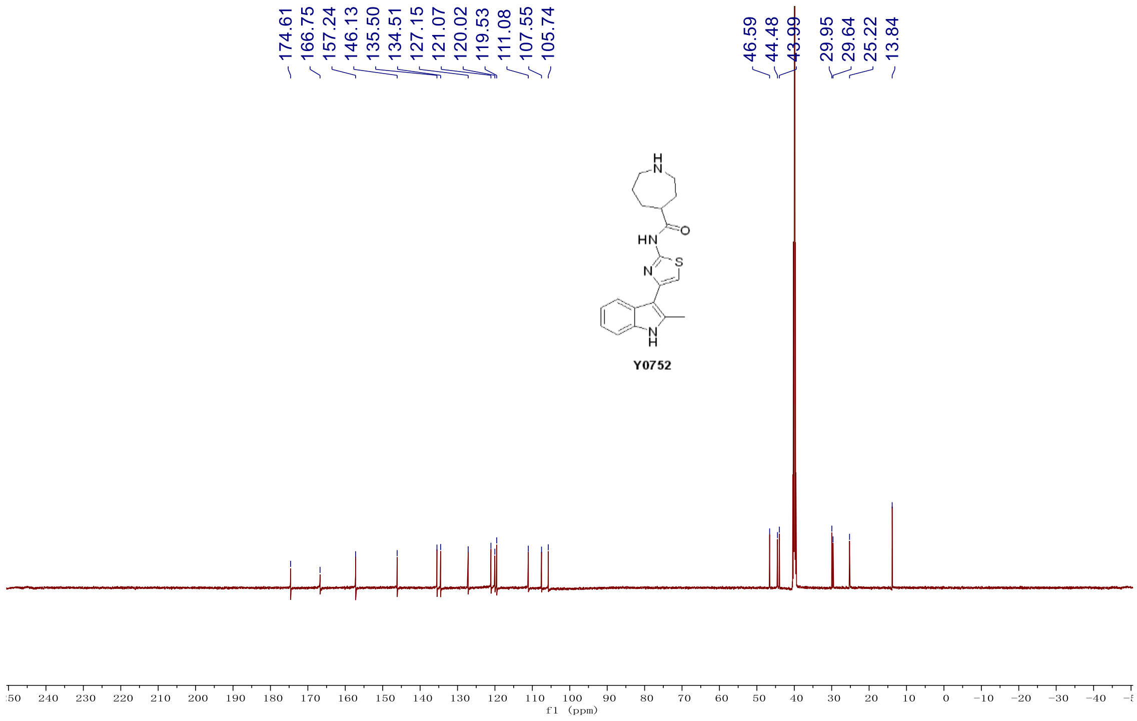

Compound **Y0752**, HRMS (ESI)

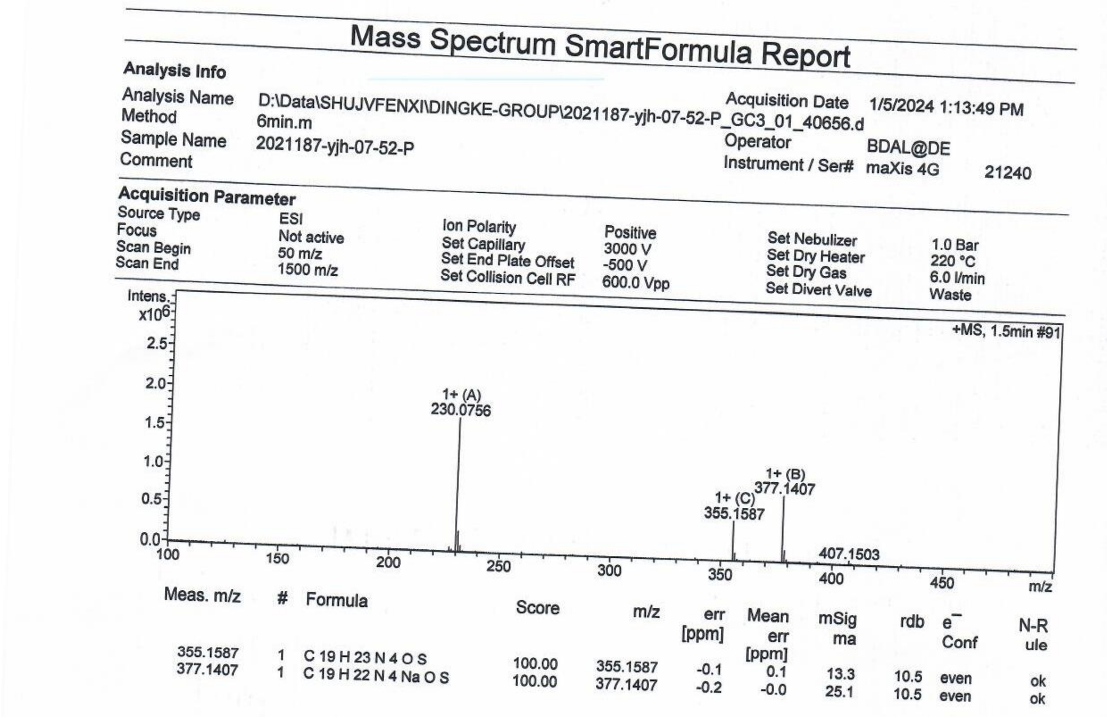

Mass Spectrum SmartFormula Report

Analysis Info

Analysis Name	D:\Data\SHUJVFENXI\DINGKE-GROUP\2021187-yjh-07-52-P_GC3_01_40656.d	Acquisition Date	1/5/2024 1:13:49 PM
Method	6min.m	Operator	BDAL@DE
Sample Name	2021187-yjh-07-52-P	Instrument / Ser#	maXis 4G 21240
Comment			

Acquisition Parameter

Source Type	ESI	Ion Polarity	Positive	Set Nebulizer	1.0 Bar
Focus	Not active	Set Capillary	3000 V	Set Dry Heater	220 °C
Scan Begin	50 m/z	Set End Plate Offset	-500 V	Set Dry Gas	6.0 l/min
Scan End	1500 m/z	Set Collision Cell RF	600.0 Vpp	Set Divert Valve	Waste

Meas. m/z	#	Formula	Score	m/z	err [ppm]	Mean err [ppm]	mSigma	rdb	e⁻ Conf	N-Rule
355.1587	1	C 19 H 23 N 4 O S	100.00	355.1587	-0.1	0.1	13.3	10.5	even	ok
377.1407	1	C 19 H 22 N 4 Na O S	100.00	377.1407	-0.2	-0.0	25.1	10.5	even	ok

Compound **Y0752**, HPLC

Single Injection Report Agilent

**Data file:**	YJH-07-52-P.dx		
**Sequence Name:**	SingleSample	**Project Name:**	1260
**Sample name:**	YJH-07-52-P	**Operator:**	SYSTEM
**Instrument:**	1260	**Injection date:**	2023-12-29 11:31:46+08:00
**Inj. volume:**	15.000 µL	**Location:**	P1-C1
**Acq. method:**	normal.amx	**Type:**	Sample
**Processing method:**	INM.pmx	**Sample amount:**	0.00
**Manually modified:**	Manual Integration		

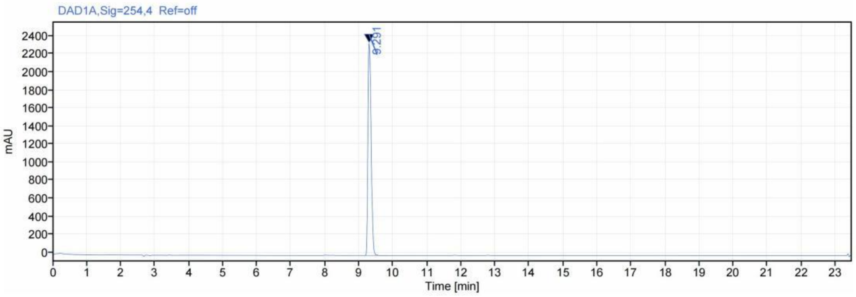

Signal: DAD1A,Sig=254,4 Ref=off

RT [min]	Type	Width [min]	Area	Height	Area%	Name
9.291	VB	0.63	15506.72	2355.17	100.00	
		**Sum**	15506.72		100.00	

Compound **Y0902 (Bio-PBITE-1)**, ^{1}H NMR (600 MHz, DMSO-d_6)

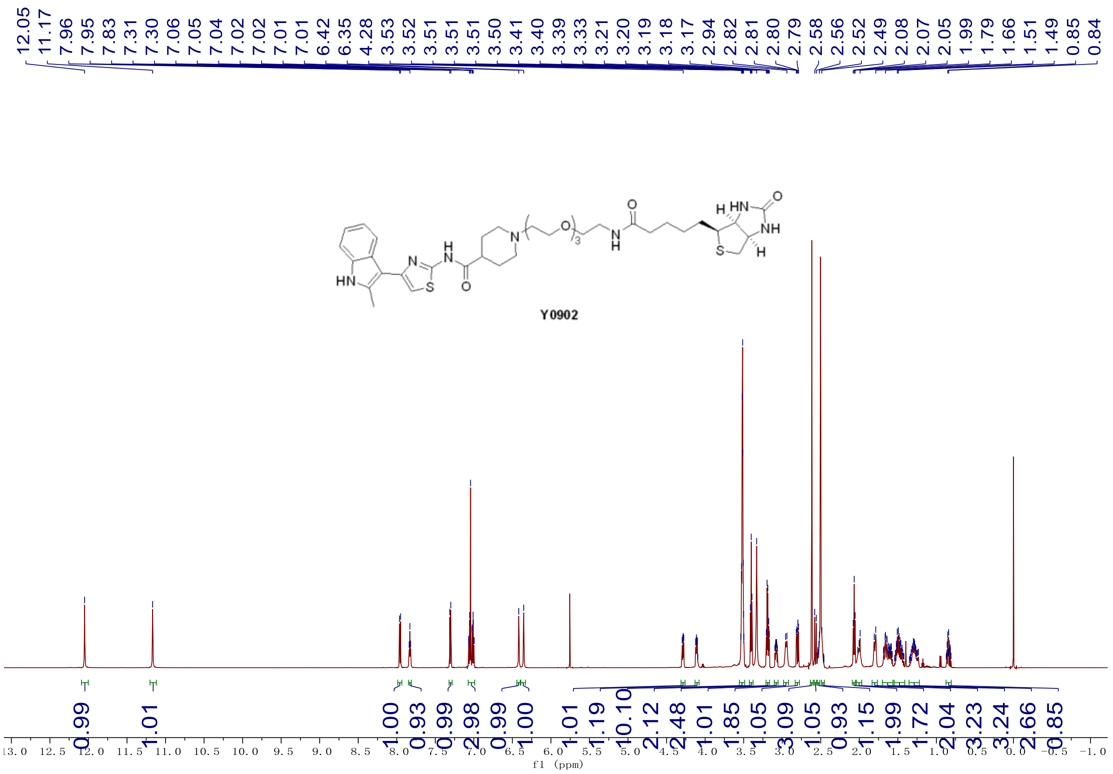

Compound **Y0902 (Bio-PBITE-1)**, ^{13}C NMR (126 MHz, DMSO-d_6)

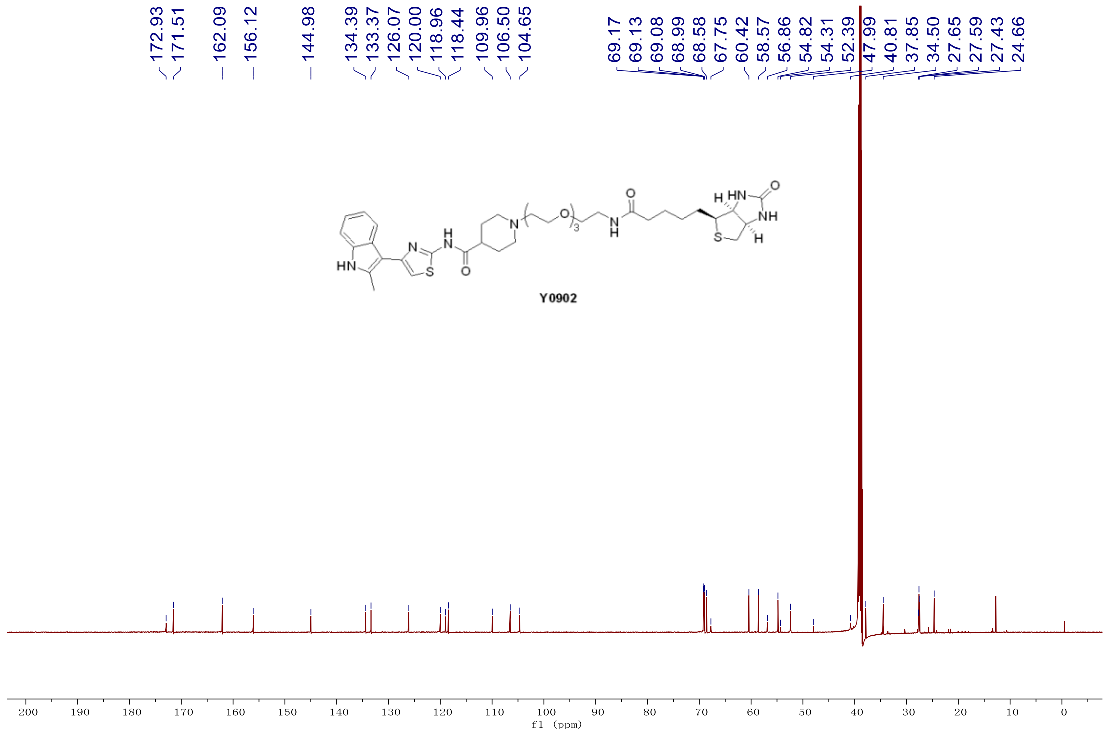

Compound **Y0902 (Bio-PBITE-1)**, HRMS (ESI)

Mass Spectrum SmartFormula Report

Analysis Info

Analysis Name	D:\Data\SHUJVFENXI\dingke-group\2024138-glh-09-02-P_RA3_01_59373.d	Acquisition Date	10/27/2025 11:53:15 AM
Method	6min.m	Operator	BDAL@DE
Sample Name	2024138-glh-09-02-P	Instrument / Ser#	maXis 4G 21240
Comment			

Acquisition Parameter

Source Type	ESI	Ion Polarity	Positive	Set Nebulizer	1.0 Bar
Focus	Not active	Set Capillary	4500 V	Set Dry Heater	220 °C
Scan Begin	50 m/z	Set End Plate Offset	-500 V	Set Dry Gas	6.0 l/min
Scan End	1500 m/z	Set Collision Cell RF	350.0 Vpp	Set Divert Valve	Waste

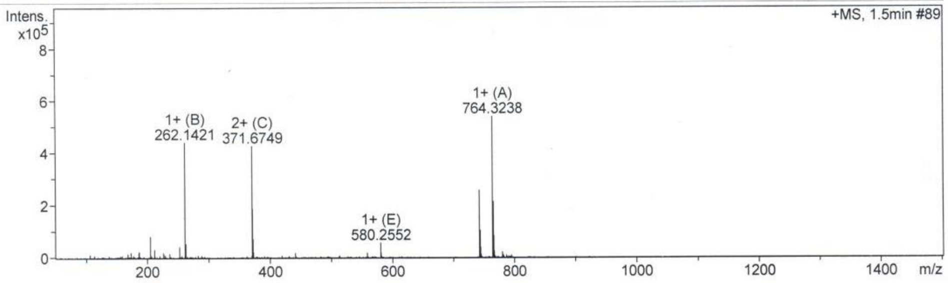

Meas. m/z	#	Formula	Score	m/z	err [ppm]	Mean err [ppm]	mSigma	rdb	e⁻ Conf	N-Rule
371.6749	1	C 36 H 53 N 7 O 6 S 2	100.00	371.6744	-1.4	-1.4	14.7	14.0	even	ok
742.3416	1	C 36 H 52 N 7 O 6 S 2	100.00	742.3415	-0.2	-0.3	23.2	14.5	even	ok
764.3238	1	C 36 H 51 N 7 Na O 6 S 2	100.00	764.3234	-0.5	-0.6	28.2	14.5	even	ok

Compound **Y0902 (Bio-PBITE-1)**, HPLC

Single Injection Report

Agilent

**Data file:**	YJH-09-02-P.dx		
**Sequence Name:**	SingleSample	**Project Name:**	1260
**Sample name:**	YJH-09-02-P	**Operator:**	SYSTEM
**Instrument:**	1260	**Injection date:**	2025-10-27 11:25:53+07:00
**Inj. volume:**	30.000 µL	**Location:**	P1-D1
**Acq. method:**	normal.amx	**Type:**	Sample
**Processing method:**	INM.pmx	**Sample amount:**	0.00
**Manually modified:**	Manual Integration		

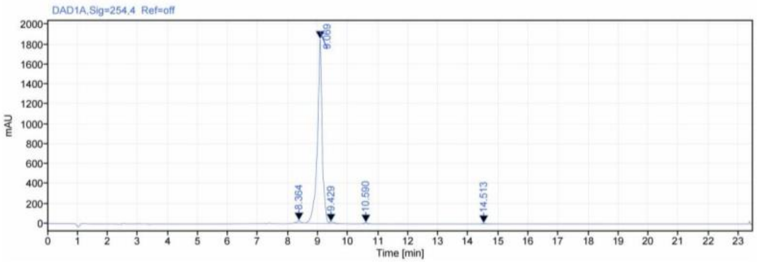

Signal: DAD1A,Sig=254,4 Ref=off

RT [min]	Type	Width [min]	Area	Height	Area%	Name
8.364	MB m	0.38	206.17	26.81	0.98	
9.069	BV	0.81	20393.43	1854.73	97.30	
9.429	VB	0.44	195.75	18.03	0.93	
10.590	MM m	0.41	106.59	12.92	0.51	
14.513	MM m	0.54	57.42	6.83	0.27	
		**Sum**	20959.36		100.00	

Compound **Y0145 (Bio-Y0747)**, ^{1}H NMR (600 MHz, DMSO-d_6)

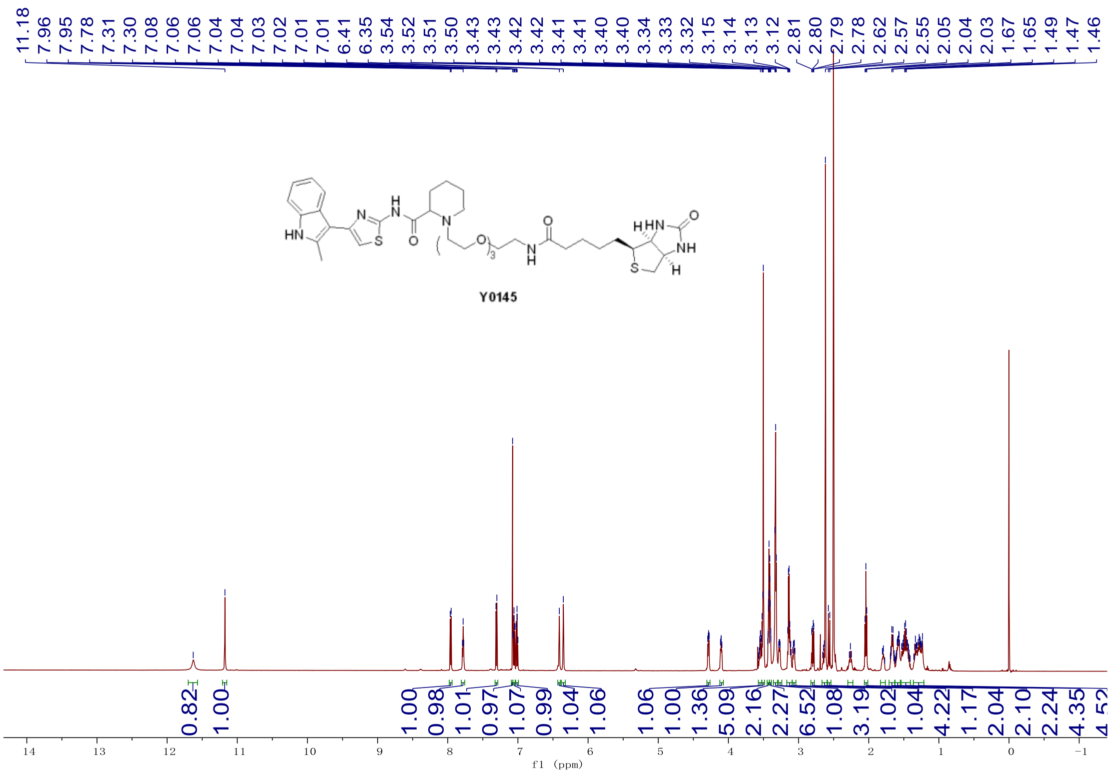

Compound **Y0145 (Bio-Y0747)**, ^{13}C NMR (126 MHz, DMSO-d_6)

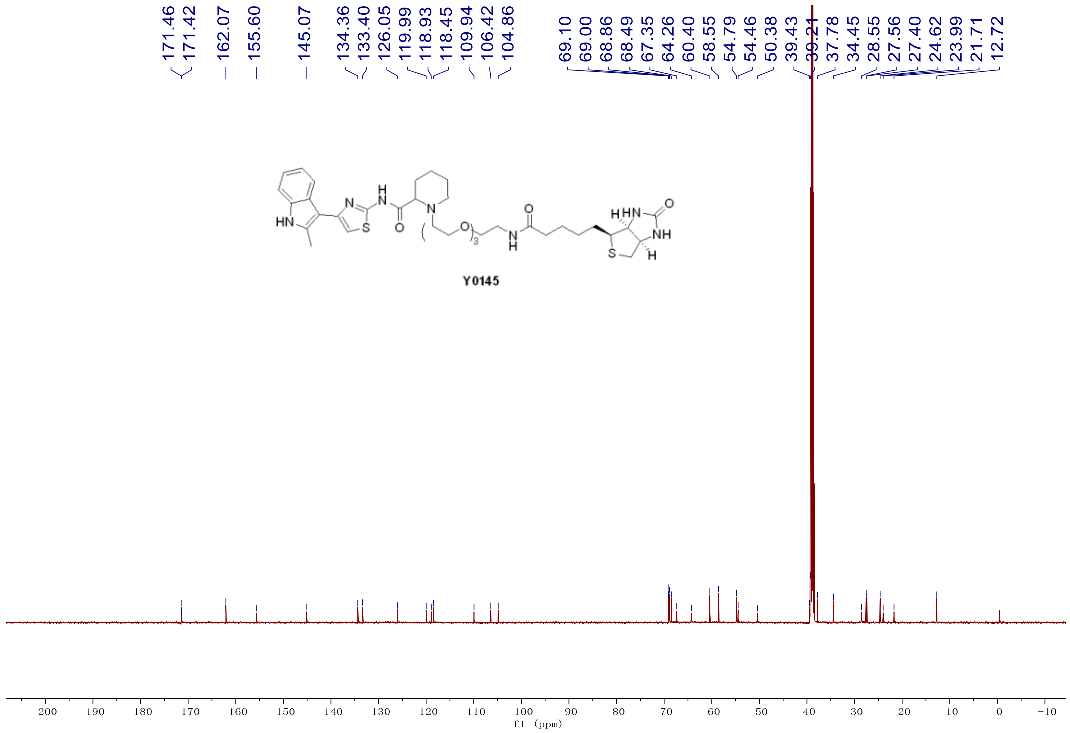

Compound **Y0145 (Bio-Y0747)**, HRMS (ESI)

Mass Spectrum SmartFormula Report

Analysis Info

Analysis Name	D:\Data\SHUJVFENXI\dingke-group\2024138-GLH-0145_RD3_01_59740.d	Acquisition Date	11/10/2025 4:11:48 PM
Method	6min.m	Operator	BDAL@DE
Sample Name	2024138-GLH-0145	Instrument / Ser#	maXis 4G 21240
Comment			

Acquisition Parameter

Source Type	ESI	Ion Polarity	Positive	Set Nebulizer	1.0 Bar
Focus	Not active	Set Capillary	4500 V	Set Dry Heater	220 °C
Scan Begin	50 m/z	Set End Plate Offset	-500 V	Set Dry Gas	6.0 l/min
Scan End	1500 m/z	Set Collision Cell RF	350.0 Vpp	Set Divert Valve	Waste

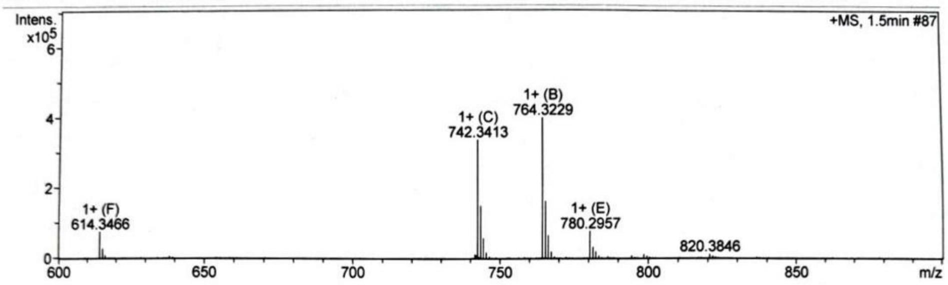

Meas. m/z	#	Formula	Score	m/z	err [ppm]	Mean err [ppm]	mSigma	rdb	e⁻ Conf	N-Rule
742.3413	1	C 36 H 52 N 7 O 6 S 2	100.00	742.3415	0.2	0.1	13.5	14.5	even	ok
764.3229	1	C 36 H 51 N 7 Na O 6 S 2	100.00	764.3234	0.7	0.5	22.8	14.5	even	ok

Compound **Y0145 (Bio-Y0747)**, HPLC

Single Injection Report Agilent

**Data file:**	GLH-0145-2.dx		
**Sequence Name:**	SingleSample	**Project Name:**	1260
**Sample name:**	GLH-0145-2	**Operator:**	SYSTEM
**Instrument:**	1260	**Injection date:**	2025-11-11 12:36:59+07:00
**Inj. volume:**	30.000 µL	**Location:**	P1-F1
**Acq. method:**	normal.amx	**Type:**	Sample
**Processing method:**	INM.pmx	**Sample amount:**	0.00
**Manually modified:**	Manual Integration		

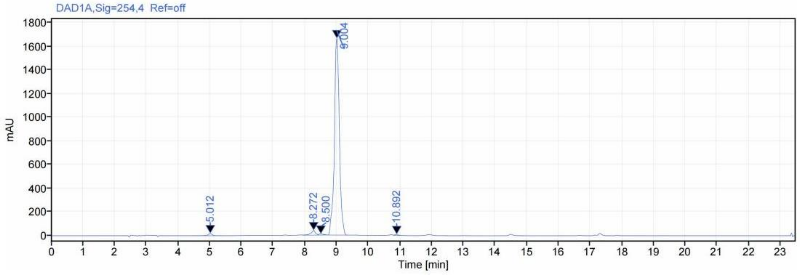

Signal: DAD1A,Sig=254,4 Ref=off

RT [min]	Type	Width [min]	Area	Height	Area%	Name
5.012	MB m	0.93	194.58	19.39	1.02	
8.272	VV	0.48	397.58	39.62	2.09	
8.500	VB	0.31	98.79	9.80	0.52	
9.004	BB	0.62	18249.76	1663.77	95.98	
10.892	VB	0.27	73.55	9.74	0.39	
		**Sum**	19014.26		100.00	

References

1. S. A. Tomlins, *et al.*, Recurrent fusion of TMPRSS2 and ETS transcription factor genes in prostate cancer. *Science* **310**, 644–648 (2005).

2. S. A. Tomlins, *et al.*, Role of the TMPRSS2-ERG gene fusion in prostate cancer. *Neoplasia* **10**, 177–188 (2008).

3. J. Luo, *et al.*, Targeting histone H2B acetylated enhanceosomes via p300/CBP degradation in prostate cancer. *Nat. Genet.* **57**, 2468–2481 (2025).

4. C. Pacini, *et al.*, A comprehensive clinically informed map of dependencies in cancer cells and framework for target prioritization. *Cancer Cell* **42**, 301-316.e9 (2024).

5. W. Lee, M. Tonelli, J. L. Markley, NMRFAM-SPARKY: enhanced software for biomolecular NMR spectroscopy. *Bioinformatics* **31**, 1325–1327 (2015).

6. B. T. Farmer 2nd, *et al.*, Localizing the NADP+ binding site on the MurB enzyme by NMR. *Nat. Struct. Biol.* **3**, 995–997 (1996).

7. H. Najem, *et al.*, Protocol to quantify immune cell distribution from the vasculature to the glioma microenvironment on sequential immunofluorescence multiplex images. *STAR Protoc.* **5**, 103079 (2024).

8. N. Cliff, *Ordinal methods for behavioral data analysis* (2014).

9. G. M. Sullivan, R. Feinn, Using effect size-or why the P value is not enough. *J. Grad. Med. Educ.* **4**, 279–282 (2012).

10. R. Mannan, *et al.*, Characterization of intercalated cell markers KIT and LINC01187 in chromophobe renal cell carcinoma and other renal neoplasms. *Int. J. Surg. Pathol.* **31**, 1027–1040 (2023).

11. R. Mannan, *et al.*, Expression of L1 cell adhesion molecule, a nephronal principal cell marker, in nephrogenic adenoma. *Mod. Pathol.* **37**, 100540 (2024).

12. R. Mannan, *et al.*, TRIM63 Overexpression in FISH-negative MiTF family altered renal cell carcinoma (MiTF RCC). *Mod. Pathol.* **38**, 100873 (2025).

13. S. Eyunni, *et al.*, Divergent FOXA1 mutations drive prostate tumorigenesis and therapy-resistant cellular plasticity. *Science* **389**, eadv2367 (2025).

14. *AJCC Cancer Staging Manual* (Springer International Publishing).
